# Spontaneously separated intermetallic Co_3_Mo from nanoporous copper as versatile electrocatalysts for highly efficient water splitting

**DOI:** 10.1038/s41467-020-16769-6

**Published:** 2020-06-10

**Authors:** Hang Shi, Yi-Tong Zhou, Rui-Qi Yao, Wu-Bin Wan, Xin Ge, Wei Zhang, Zi Wen, Xing-You Lang, Wei-Tao Zheng, Qing Jiang

**Affiliations:** Key Laboratory of Automobile Materials (Jilin University), Ministry of Education, School of Materials Science and Engineering, and Electron Microscopy Center, Jilin University, Changchun, 130022 China

**Keywords:** Catalyst synthesis, Renewable energy, Electrocatalysis

## Abstract

Developing robust nonprecious electrocatalysts towards hydrogen/oxygen evolution reactions is crucial for widespread use of electrochemical water splitting in hydrogen production. Here, we report that intermetallic Co_3_Mo spontaneously separated from hierarchical nanoporous copper skeleton shows genuine potential as highly efficient electrocatalysts for alkaline hydrogen/oxygen evolution reactions in virtue of in-situ hydroxylation and electro-oxidation, respectively. The hydroxylated intermetallic Co_3_Mo has an optimal hydrogen-binding energy to facilitate adsorption/desorption of hydrogen intermediates for hydrogen molecules. Associated with high electron/ion transport of bicontinuous nanoporous skeleton, nanoporous copper supported Co_3_Mo electrodes exhibit impressive hydrogen evolution reaction catalysis, with negligible onset overpotential and low Tafel slope (~40 mV dec^−1^) in 1 M KOH, realizing current density of −400 mA cm^−2^ at overpotential of as low as 96 mV. When coupled to its electro-oxidized derivative that mediates efficiently oxygen evolution reaction, their alkaline electrolyzer operates with a superior overall water-splitting output, outperforming the one assembled with noble-metal-based catalysts.

## Abstract (web summary)

Electrochemical water splitting is an attractive energy conversion technology, but it usually suffers from low efficiency. Here, the authors report intermetallic Co_3_Mo integrated on porous Cu as highly efficient electrocatalysts for alkaline HER/OER due to in-situ hydroxylation and electro-oxidation.

## Introduction

Electrochemical water splitting powered by electricity from renewable energy sources (e.g., solar or wind) is an attractive energy-conversion technology that makes use of molecular hydrogen as a clean energy carrier in the energy framework of the water cycle^1–3^. It promises a sustainable scenario to store/deliver renewable energy without any greenhouse-gas emission for meeting future energy demands^3–6^. However, its widespread implementation is persistently plagued by the low efficiency of water electrolysis^1–6^, particularly in water–alkali electrolyzers with nickel and/or stainless steel as electrodes or electrocatalysts^5–12^, due to their high operation overpotentials and poor durability for the electrocatalytic hydrogen/oxygen evolution reactions (HER/OER)^13–16^. Such dilemma urgently calls for developing more efficient HER/OER electrocatalytic materials to lower the overall-splitting overpotential^6,9,12^, with the versatile requirements of amply accessible surface, highly reactive sites and fast electron-transfer and mass-transport pathways^15–20^. To date, nanostructured noble-metal platinum (Pt)^9,21–23^ and iridium/ruthenium oxides (IrO_2_ or RuO_2_)^8,24^ are the benchmark HER and OER catalysts with the highest intrinsic activities, respectively, but the scarcity and high cost substantially hinder their widespread utilization^6–9,13^. Moreover, nanostructured Pt, typically carbon-supported Pt nanoparticles (Pt/C), not only suffers from poor durability but encounters 2 or 3 orders of magnitude lower HER activity in alkaline media than in acidic electrolytes^8–12,22^. With an aim to replace these noble-metal catalysts by Earth-abundant and cost-effective catalysts^9,13,15,25^, many non-noble transition-metal compounds (such as Mo, W, Fe, Co, and Ni carbides^25–27^, phosphides^28,29^, and sulfides^30,31^) and their derivatives have been developed for the HER/OER in alkaline environments^32–34^. Nevertheless, few of these materials satisfy the practical requirements^9,13,20^, i.e., high current density at low working overpotential, due to either poor electron transfer and/or insufficient catalytic activity^24–35^. Despite nanostructuring as a general strategy could improve their HER/OER catalysis by increasing active sites^12,15,16,18,25^, most nanocatalysts usually have to be immobilized on a conductive substrate using insulative polymer binders, which inevitably leads to supplementary interfaces linked to conductivity issues.

Here, we report intermetallic Co_3_Mo nanoparticles that are seamlessly integrated on the surface of hierarchical nanoporous Cu skeleton (Co_3_Mo/Cu) via a spontaneous phase separation during a chemical dealloying process as high-performance HER/OER electrocatalytic materials in alkaline media as a consequence of in situ hydroxylation and electro-oxidation. Owing to the hydroxylated Co_3_Mo having an optimal hydrogen-binding energy (HBE) associated with the fast HER reaction rate and the hierarchical nanoporous Cu skeleton facilitating electron transfer and HO^−^/H_2_O mass transport, the self-supported nanoporous Co_3_Mo/Cu electrodes exhibit negligible onset overpotential and low Tafel slope of 40 mV dec^−1^ in 1 M KOH. They reach a current density of −400 mA cm^−2^ at the iR-corrected overpotential of as low as 96 mV. When triggered by electro-oxidation, there in-situ forms Mo-doped Co_3_O_4_ nanoflakes on the nanoporous CuO/Cu skeleton to accelerate the OER. The alkaline electrolyzer assembled with the nanoporous Co_3_Mo/Cu as the cathode and its derivative oxide as the anode only takes 1.65 V to achieve ~145 mA cm^−2^, along with good stability, in a brine electrolyte containing 1 M KOH and 0.5 M NaCl. These electrochemical properties outperform some of the best electrocatalysts previously reported and make them promising candidates for overall electrochemical water splitting.

## Results

### Preparation and structural characterization

**Fig. 1:**
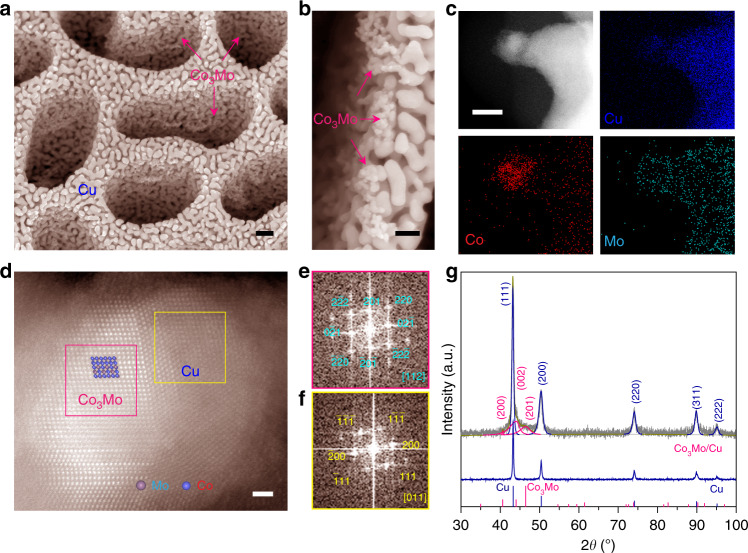
Microstructures and chemical properties. **a** Typical cross-sectional SEM image of Co_3_Mo/Cu electrode prepared by chemically dealloying Cu_8_Co_3_Mo_1_Al_88_ alloy precursor, displaying a three-dimensional bicontinuous and bimodal nanoporous architecture. Scale bar, 200 nm. **b** High-resolution cross-sectional SEM image of Co_3_Mo/Cu electrodes with a number of intermetallic Co_3_Mo nanoparticles in-situ integrated on Cu ligaments. Scale bar, 100 nm. **c** STEM image and the corresponding STEM-EDS elemental mappings of Cu, Co and Mo, demonstrating the Co_3_Mo nanoparticles grown on Cu ligaments. Scale bar, 10 nm. **d** Atomic resolution HAADF-STEM image of intermetallic Co_3_Mo nanoparticles grown on Cu ligament with a coherent interface structure. Inset: atomic structure of intermetallic Co_3_Mo. Scale bar, 1 nm. **e**, **f** FFT patterns of Co_3_Mo (**e**) and Cu (**f**) in the selected areas in (**d**). **g** XRD patterns of nanoporous Co_3_Mo/Cu hybrid electrode and bare nanoporous Cu electrode. The line patterns show reference cards 04-0836 and 29-0488 for Cu and Co_3_Mo phases according to JCPDS, respectively.

To develop electrocatalytic materials for practical application, self-supported monolithic nanoporous Co_3_Mo/Cu electrodes are fabricated by a facial and scalable dealloying technology associated with a spontaneous phase separation, which can be extended to develop various monolithic nanoporous alloy/metal hybrid electrode materials. Therein, both the hierarchical structures and components are controlled by making use of quasi-eutectic Cu_12−*x*−*y*_Co_*x*_Mo_*y*_Al_88_ (*x* = 0 or 3, *y* = 0 or 1 at%) alloy precursors (Supplementary Fig. 1), which are primarily composed of immiscible multiphase α-Al and CuAl_2_ without/with additional intermetallic Co_3_Mo^36,37^, Co_2_Al_9_, or MoAl_12_ (Supplementary Fig. 2). As illustrated by representative scanning electron microscopy (SEM) backscattered electron image and transmission electron microscope (TEM) image for the Cu_8_Co_3_Mo_1_Al_88_ alloy (Supplementary Fig. 3a, b), there are two distinctly contrasted phases with ~300 nm width. One is α-Al phase and the other corresponds to Cu-rich mixture of intermetallic CuAl_2_, Co_3_Mo, Co_2_Al_9_, and MoAl_12_, attested by their XRD patterns (Supplementary Fig. 2b) and energy-dispersive X-ray spectroscopy (EDS) elemental mappings (Supplementary Fig. 3a). The quasi-eutectic multiphase structure enables a bimodal nanoporous Cu skeleton with large channels and small nanopores via a two-step etching process^38–41^, i.e., the fast dissolution of sub-micrometer-sized α-Al phase and then the slow dealloying of less-noble Al component in the intermetallic CuAl_2_ phase. Figure 1a shows a typical cross-sectional SEM image for nanoporous Co_3_Mo/Cu electrode that is prepared by chemically dealloying the Cu_8_Co_3_Mo_1_Al_88_ alloy in KOH electrolyte. It displays a hierarchical nanoporous Cu skeleton with ~300 nm large channels and ~25 nm small nanopores. Owing to the poor miscibility of intermetallic Co_3_Mo with Cu matrix^37,42^, ultrasmall intermetallic Co_3_Mo nanoparticles with diameter of ~10 nm are spontaneously separated and in situ integrated on the interconnective Cu ligaments as a result of the Cu surface diffusion during the etching processes (Fig. 1b). X-ray photoelectron spectroscopy (XPS) analysis demonstrates the presence of Co and Mo with an atomic ratio of 25/3, beside Cu skeleton and residual Al (Supplementary Fig. 4). This is consistent with EDS elemental mapping (Supplementary Fig. 5) and inductively coupled plasma optical emission spectroscopy (ICP-OES) measurements. Scanning TEM (STEM) EDS mapping of nanoporous Co_3_Mo/Cu electrode attests the formation of Co_3_Mo nanoparticles with diameter of ~10 nm on the protuberant Cu ligament (Fig. 1c), in addition to some Co and Mo atoms with some *OH ligands due to selectively removing the Al component in the Co_2_Al_9_ and MoAl_12_. While for the residual Al atoms, STEM-EDS mapping and low-energy ion scattering (LEIS) spectrum illustrate that they are mainly in the interior of Cu skeleton (Supplementary Fig. 6). The atomic structure of nanoporous Cu supported Co_3_Mo nanoparticles is further characterized by high-angle annual-dark-field STEM (HAADF-STEM). Figure 1d shows a representative HAADF-STEM image of intermetallic Co_3_Mo nanoparticles in-situ grown on Cu ligament, which are evidenced by their corresponding fast Fourier transform (FFT) patterns (Fig. 1e, f). There is no sharp Co_3_Mo/Cu interface to be observed, implying that the Co_3_Mo nanoparticles are highly coherent with the Cu substrate. Such unique architecture not only enables electron transfer but also maintains exceptional stability. X-ray diffraction (XRD) characterization of nanoporous Co_3_Mo/Cu electrode verifies the hybrid structure, with two sets of diffraction patterns (Fig. 1g): the evident diffraction peaks at 2*θ* = ~40.6°, ~44.0°, and ~46.4° corresponding to the (200), (002), and (201) planes of intermetallic Co_3_Mo in space group P 63/mmc (JCPDS 29-0488)^37,42,43^, and the ones else assigned to Cu (JCPDS 04-0836). This is in contrast with the XRD patterns of nanoporous Cu with/without Mo or Co incorporation, wherein they cannot be detected due to either too little or too small clusters on the Cu ligaments despite their similar geometric structure of nanoporous electrodes (Supplementary Figs. 7–9).

**Fig. 2:**
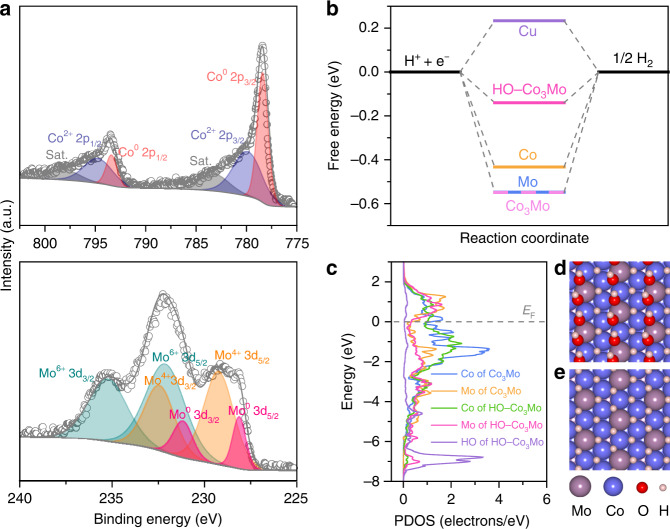
XPS characterization and DFT simulation. **a** High-resolution Co 2p (top) and Mo 3d (bottom) XPS spectra of nanoporous Co_3_Mo/Cu electrode. **b** Free-energy diagram for hydrogen (H*) adsorption on 1/2 ML HO*-adsorbed Co_3_Mo surface and pure Cu, Co, Mo, and Co_3_Mo surfaces. **c** Partial density of states (PDOS) of Co, Mo in Co_3_Mo and Co, Mo, HO in the 1/2 ML HO*-adsorbed Co_3_Mo. **d**, **e** Schematic structures of Co_3_Mo with/without the 1/2 ML HO* adsorption.

Figure 2a shows Co 2p and Mo 3d XPS spectra in the nanoporous Co_3_Mo/Cu electrode. In addition to their metallic states that correspond to the internal Co and Mo atoms in the intermetallic Co_3_Mo, the surface Co atoms exhibit Co^2+^ state^44,45^ due to the surface adsorption of hydroxyl groups while surface Mo atoms are in Mo^4+^ and Mo^6+^ states (Supplementary Fig. 10)^46^. Nevertheless, these oxidized Co and Mo surface atoms thermodynamically prefer to be electro-reduced to form partially hydroxylated Co_3_Mo surface in the HER potential region at pH = 14, as verified by initial two linear scanning voltammetry curves in 1 M KOH electrolyte (Supplementary Fig. 11)^47^. As demonstrated by density functional theory (DFT) calculations based on the meta-generalized gradient approximation (meta-GGA) with strongly constrained appropriately normed (SCAN) functional (Supplementary Fig. 12a), the coverage of hydroxyl groups on the Co_3_Mo(002) surface decreases as the potential decreases from 0.6 to −0.6 V vs. the reversible hydrogen electrode (RHE). At the potential of 0 V vs. RHE, DFT simulations demonstrate that the hydroxyl groups tend to be adsorbed on the Co–Co–Mo hollow sites with a coverage of 1/2 (Supplementary Fig. 12b). Considering that the practical catalyst surface is always in co-adsorption with HO^−^ ions in alkaline environments^5,7,9,48,49^, the free energy for atomic hydrogen adsorption (Δ*G*_H*_) for nonprecious metal catalysts is calculated on the basis of such atomic configuration. The Δ*G*_H*_ for the partially hydroxylated Co_3_Mo(002) becomes almost thermoneutral, suggesting that it is more favorable for hydrogen adsorption/desorption than bare ones, as well as the monometallic Co and Mo (Fig. 2b). This is due to the adsorption of hydroxyl groups remarkably weakening the HBE (−0.36 eV) of their adjacent Co-Co-Mo hollow sites in comparison with the value of bare Co_3_Mo(002) surface (−0.77 eV), as illustrated in their partial density of states (PDOS) (Fig. 2c) and schematic atomic structures (Fig. 2d, e).

### Electrochemical characterizations

**Fig. 3:**
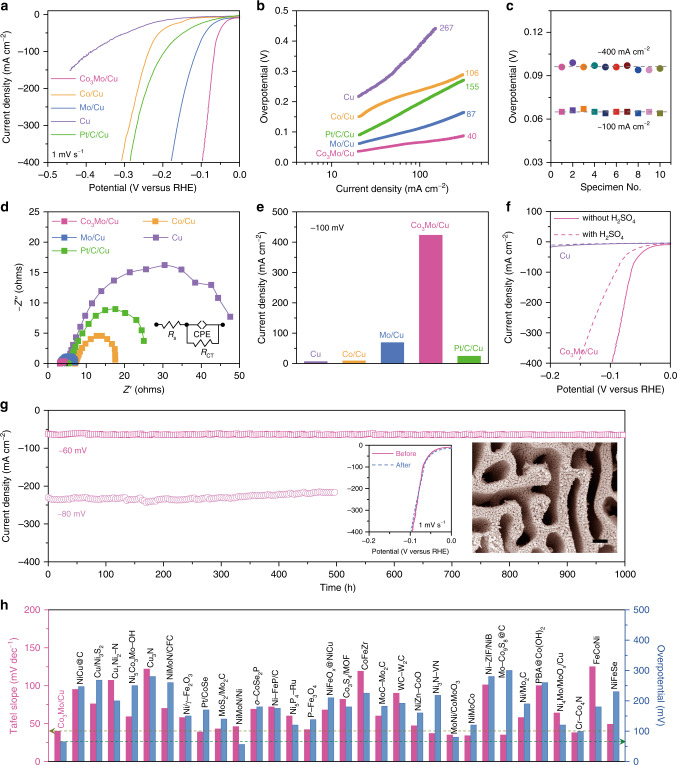
Electrochemical characterization on HER properties. **a** iR-corrected HER polarization curves for self-supported nanoporous Co_3_Mo/Cu, Co/Cu, Mo/Cu, and Cu electrodes, as well as commercially available Pt/C immobilized on nanoporous Cu (Pt/C/Cu) in 1 M KOH. Scan rate: 1 mV s^−1^. **b** Comparison of the Tafel plots of different electrocatalytic materials obtained from the HER polarization curves in panel (**a**). **c** Overpotentials at current densities of 100 and 400 mA cm^−2^ for ten nanoporous Co_3_Mo/Cu electrodes that are prepared by the same alloying/dealloying procedure. **d** EIS spectra of nanoporous Co_3_Mo/Cu, Co/Cu, Mo/Cu, and bare Cu electrodes and nanoporous Pt/C/Cu. **e** Comparison of current density at −100 mV for nanoporous Co_3_Mo/Cu electrode with the values of nanoporous Cu, Co/Cu, Mo/Cu electrodes, and nanoporous Pt/C/Cu. **f** iR-corrected HER polarization curves of nanoporous Co_3_Mo/Cu and bare Cu electrodes before and after H_2_SO_4_ treatment. **g** Long-term stability measurement of nanoporous Co_3_Mo/Cu electrode at the overpotentials of 60 and 80 mV for more than 1000 and 500 h, respectively. The negligible current fluctuation is due to the depletion/replenishment of electrolyte. Insets: SEM image after the durability measurement at 60 mV for 1000 h and polarization curves before and after the durability measurement. Scale bar: 300 nm. **h** Tafel slope and overpotential at 100 mA cm^−2^ of nanoporous Co_3_Mo/Cu electrode, comparing with the values of representative HER catalysts reported previously.

To evaluate the electrocatalysis, all nanoporous electrode materials are directly used as working electrodes for electrochemical measurements, which are performed in N_2_-saturated 1 M KOH electrolyte in a classic three-electrode configuration with a graphite counter electrode and an Ag/AgCl reference electrode. According to the calibration experiment (Supplementary Fig. 13), all potentials are iR corrected and calibrated with respect to the RHE. Figure 3a compares typical HER polarization curve of nanoporous Co_3_Mo/Cu electrode with those of nanoporous Mo/Cu, Co/Cu, and bare Cu electrodes, as well as that of commercially available Pt/C catalyst immobilized on the nanoporous Cu electrode by dint of Nafion as polymer binder (Pt/C/Cu). Because of the presence of hydroxylated intermetallic Co_3_Mo nanoparticles, the nanoporous Co_3_Mo/Cu electrode exhibits an overpotential of as low as ~12 mV at current density of 10 mA cm^−2^, in sharp contrast with nanoporous Mo/Cu (~25 mV), Co/Cu (~116 mV), and Cu electrodes (~151 mV) with either too high or too low HBE^6,11,18^. Furthermore, this value is ~38 mV lower than that of nanoporous Pt/C/Cu despite the constituent Pt is usually expected as a benchmark catalyst to efficiently boost the HER. The superior electrocatalytic activity of nanoporous Co_3_Mo/Cu is also manifested by the low Tafel slope of ~40 mV dec^−1^ that corresponds to the Volmer–Heyrovsky mechanism (Fig. 3b)^5,6,10,12,13,25^. As a result, the nanoporous Co_3_Mo/Cu only takes the iR-corrected overpotential of ~96 mV to reach a current density of −400 mA cm^−2^, much lower than nanoporous Mo/Cu (~178 mV) and Co/Cu electrodes (~308 mV). The overpotential of the nanoporous Pt/C/Cu at −400 mA cm^−2^ is as high as ~286 mV probably due to the polymer binder that covers some electroactive sites. To demonstrate the reproducibility, ten nanoporous Co_3_Mo/Cu electrodes are produced by the same dealloying procedure. Supplementary Fig. 14 shows their HER polarization curves with almost the same overpotentials of ~65 and ~96 mV at −100 and −400 mA cm^−2^ (Fig. 3c), respectively, indicating the excellent reproducibility. The substantially boosted reaction kinetics of the HER on the nanoporous Co_3_Mo/Cu is further revealed by electrochemical impedance spectroscopy (EIS) analysis. As shown in the Nyquist plots (Fig. 3d), the EIS spectrum of nanoporous Co_3_Mo/Cu displays a characteristic semicircle with a very small diameter that represent a low charge transfer resistance (*R*_CT_). Based on the general descriptors in the equivalent circuit (inset of Fig. 3d), the *R*_CT_ value is determined to be ~1 Ω for the nanoporous Cu/Co_3_Mo, much lower than those of nanoporous Cu/Mo (~3 Ω), Cu/Co (~10 Ω), Cu (~39 Ω) electrodes (Supplementary Fig. 15).

Double-layer capacitance measurements demonstrate that the electrochemical surface area (ECSA) cannot account for ~6- or 46-fold enhancement in geometric current density of nanoporous Co_3_Mo/Cu electrode in view that its ECSA is only 0.7- or 1.1-fold that of nanoporous Mo/Cu or Co/Cu (Fig. 3e, Supplementary Fig. 16). This fact indicates the significant role of intermetallic Co_3_Mo in the exceptional HER activity of nanoporous Co_3_Mo/Cu, justified by H_2_SO_4_ treatment that partially removes the electroactive Co_3_Mo nanoparticles (Supplementary Fig. 17). As shown in Fig. 3f, the H_2_SO_4_-treated nanoporous Co_3_Mo/Cu exhibits evident degradation of HER catalysis, with about threefold reduction in current density at the overpotential of 100 mV, relative to the pristine one. With an assumption that the dramatic reduction in geometric current density results from the ECSA change due to the loss of both Co_3_Mo nanoparticles and Cu kinks or edges on ligament surface (Supplementary Fig. 18), the intrinsic activity of Co_3_Mo is evaluated to be 0.48 mA cm^−2^_ECSA_, ~830-fold higher than that of bare Cu (0.58 μA cm^−2^_ECSA_) (Supplementary Fig. 19a). The outstanding electrocatalytic activity of Co_3_Mo is also confirmed by the turnover frequency that is estimated to be 0.775 s^−1^ at the overpotential of 100 mV (Supplementary Fig. 19b, Supplementary Note 1), higher than some of the best nonprecious HER catalysts reported previously in 1 M KOH (Supplementary Table 1).

The durability of nanoporous Co_3_Mo/Cu electrode is tested at −60 and −80 mV vs. RHE for 1000 and 500 h in 1 M KOH, respectively. As shown in Fig. 3g, there is not evident fluctuation in their corresponding current densities of ~60 and ~210 mA cm^−2^ because of the steady nanoporous structure, in which the constituent intermetallic Co_3_Mo nanoparticles are seamlessly integrated on the firm metal skeleton. In the tested electrolyte, there are no Co and Mo ions to be detected by ICP-OES except for some detectable Cu and Al ions (Supplementary Table 2). Nevertheless, after the long-term durability measurements, the nanoporous Co_3_Mo/Cu electrode still keeps the original structure and exhibits almost the same polarization curve as the initial one (insets of Fig. 3g). All these electrochemical properties indicate that the nanoporous Co_3_Mo/Cu electrode can exhibit impressive electrocatalytic performance at mild conditions, i.e., in 1 M KOH electrolyte at room temperature, in contrast with Ni–Mo catalysts supported by stainless steel or Ni substrates even under higher temperatures or/and KOH concentrations^50–53^. Compared with state-of-the-art transition-metal compounds and alloys, the nanoporous Co_3_Mo/Cu electrode is one of the best nonprecious electrocatalytic materials (Fig. 3h, Supplementary Tables 3 and 4)^6,10,13,15,26–31^.

**Fig. 4:**
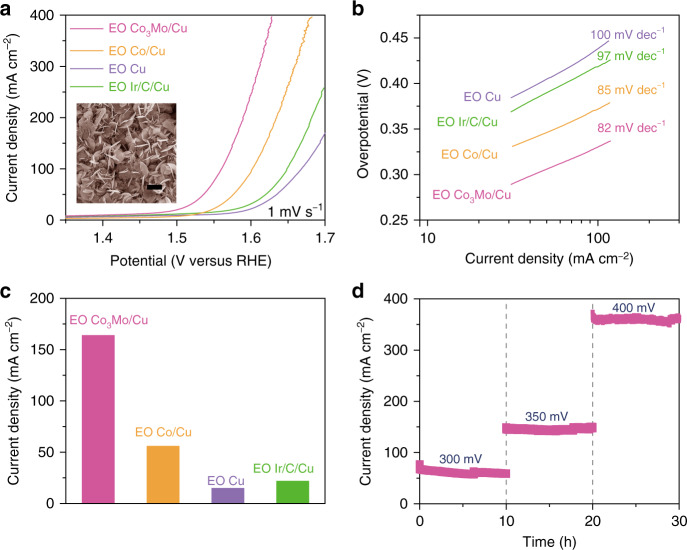
Electrochemical characterization of OER properties. **a** iR-corrected OER polarization curves of the electro-oxidized nanoporous Co_3_Mo/Cu (EO Co_3_Mo/Cu), Co/Cu (EO Co/Cu), and bare Cu (EO Cu) in 1 M KOH electrolyte, along with commercially available Ir/C supported by nanoporous Cu (Ir/C/Cu). Inset: typical SEM image of electro-oxidized nanoporous Co_3_Mo/Cu. Scale bar: 1 μm. **b** Tafel plots comparing the Tafel slopes of different OER electrocatalysts according to the polarization curves in panel (**a**). **c** Comparison of current density of the EO Co_3_Mo/Cu electrode at the overpotential 350 mV with those of EO Co/Cu, EO Cu and nanoporous Ir/C/Cu. **d** Stability measurements of the electro-oxidized nanoporous Co_3_Mo/Cu at overpotential of 300, 350, and 400 mV for 30 h.

Besides directly working as the outstanding HER electrocatalyst, the nanoporous Co_3_Mo/Cu can also serve as a good pre-electrocatalyst for the OER. After a facile electro-oxidation procedure at a potential of 1.57 V vs. RHE (Supplementary Fig. 20a), there form a large number of Mo-doped Co_3_O_4_ nanoflakes to be integrated with partially oxidized CuO/Cu skeleton (EO Co_3_Mo/Cu), as illustrated by SEM (inset of Fig. 4a) and high-resolution TEM images (Supplementary Fig. 20b). Distinguished from the CuO signals in the EO Co_3_Mo/Cu electrode, which keep the same Raman characteristics as the ones in the EO Cu, the characteristic Raman bands at 183, 460, 502, and 658 cm^−1^ remarkably blue shift relative to the ones of pristine Co_3_O_4_ (Supplementary Fig. 20c)^54^, implying the incorporation of Mo into the Co_3_O_4_ nanoflakes^55^. This is further verified by XPS analysis and SEM-EDS elemental mappings of Co, Mo, and O. As a result of uniform incorporation of Mo in the Co_3_O_4_ (Supplementary Fig. 21c–e), the XPS characteristic peaks of the Co 2p in the EO Co_3_Mo/Cu electrode shift to lower binding energy compared with the ones in the EO Co/Cu (Supplementary Fig. 20d), suggesting the strong electron interactions between Mo and Co_3_O_4_^56^. On account of the enhanced electrocatalytic activity of the Mo-doped Co_3_O_4_, the EO Co_3_Mo/Cu electrode with a hierarchical nanoporous architecture shows a superior OER activity in 1 M KOH electrolyte, compared with the electro-oxidized nanoporous Co/Cu, Cu electrodes (EO Co/Cu, EO Cu) (Fig. 4a). The OER current density of EO Co_3_Mo/Cu electrode increases dramatically from the onset overpotential of ~261 mV, ~86 mV lower than that of Ir/C supported by nanoporous Cu (Ir/C/Cu) (~347 mV). As a result of low Tafel slope of ~82 mV dec^−1^ (Fig. 4b), the EO Co_3_Mo/Cu can deliver a geometric current density of ~164 mA cm^−2^ only at the overpotential of 350 mV (Fig. 4c). Owing to the steady architecture, the EO Co_3_Mo/Cu electrode exhibits a good electrochemical durability at the overpotentials of 300, 350, and 400 mV for each 10 h in 1 M KOH electrolyte (Fig. 4d). As shown in Supplementary Fig. 22, the EO Co_3_Mo/Cu electrode keeps the initial morphology and elemental distributions of Co, Mo, and O after the durability test. Furthermore, there are not evident changes in Raman spectra and Co 2p, Mo 3d, and O 1s XPS spectra (Supplementary Figs. 23 and 24), demonstrating the stability of surface oxides. For comparison, nanoporous NiFe/Cu electrode and its electro-oxidized derivative (EO NiFe/Cu) are also prepared by the same methods (Supplementary Fig. 25a, c). Whereas the EO NiFe/Cu exhibits slightly higher OER activity than the EO Co_3_Mo/Cu^57^, the HER activity of nanoporous NiFe/Cu electrode is much lower than that of nanoporous Co_3_Mo/Cu electrode (Supplementary Fig. 25b, d).

### Electrochemical performance of alkaline electrolyzers

**Fig. 5:**
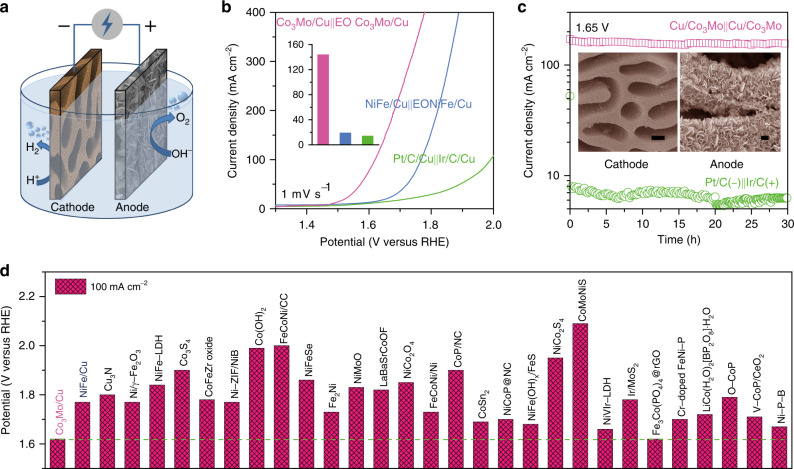
Electrochemical performance in water splitting. **a** Schematic illustration of water splitting powered by electric energy, where an alkaline electrolyzer is constructed with nanoporous Co_3_Mo/Cu and EO Co_3_Mo/Cu electrodes as cathode and anode, respectively. **b** Polarization curves for electrocatalytic overall water splitting of the devices constructed with nanoporous Co_3_Mo/Cu and EO Co_3_Mo/Cu electrodes, nanoporous NiFe/Cu and EO NiFe/Cu electrodes, or Pt/C/Cu and Ir/C/Cu, in the aqueous electrolyte of 1 M KOH and 0.5 M NaCl. Inset: Comparison of current density at 1.65 V. **c**, Durability test of the electrolyzer constructed with nanoporous Co_3_Mo/Cu and EO Co_3_Mo/Cu electrodes at 1.65 V, comparing with that of the device assembled with Pt/C/Cu and Ir/C/Cu. Inset: Typical SEM images of nanoporous Co_3_Mo/Cu cathode and EO Co_3_Mo/Cu anode after durability test. Scale bar: 400 nm. **d** Comparison of working voltage of nanoporous Co_3_Mo/Cu-based alkaline electrolyzer at the current density of 100 mA cm^−2^ with the values of the ones reported previously.

The outstanding electrocatalytic properties of nanoporous Co_3_Mo/Cu and its electro-oxidized derivative for the HER and OER demonstrate that they show genuine potential as cathode and anode materials for practical water electrolysis (Fig. 5a). Figure 5b presents a typical polarization curve of an alkaline electrolyzer that is constructed with the nanoporous Co_3_Mo/Cu as the cathode and the EO Co_3_Mo/Cu as the anode. Within the brine electrolyte containing 1 M KOH and 0.5 M NaCl, the onset overpotential of this electrolyzer is as low as ~230 mV, distinguished from most electrocatalysts that tend to be corroded and/or deactivated in such environments^28,58^. Notably, the nanoporous Co_3_Mo/Cu-based electrolyzer delivers the current density of 100 mA cm^−2^ at a low potential of 1.62 V for the overall water splitting, outperforming the ones assembled with nonprecious electrode materials previously reported (Fig. 5d, Supplementary Table 5)^33,35,46^. At the overpotential of 420 mV, the water-splitting output of this electrolyzer reaches ~145 mA cm^−2^, more than 7- or 10-fold higher than the one constructed with nanoporous NiFe/Cu and EO NiFe/Cu, or Pt/C/Cu and Ir/C/Cu, respectively (inset of Fig. 5b). During the stability measurement at the voltage of 1.65 V, the output of this alkaline electrolyzer is highly stable at ~145 mA cm^−2^, in contrast with the poor behavior of Pt/C/Cu-Ir/C/Cu electrolyzer, which is performed at the same voltage but with much lower current densities (Fig. 5c). Moreover, both nanoporous Co_3_Mo/Cu and EO Co_3_Mo/Cu electrodes still maintain their initial microstructures after the stability measurement (insets of Fig. 5c).

## Discussion

In the water electrolysis, the HER/OER taking place at the cathode/anode is always associated with mass transports of ions/molecules toward/from the electrochemical surface in the electrolyte, electron transfer from current collector to active sites in the solid electrode, and redox reactions at the active site/electrolyte interface^59^. These indispensable processes require a versatile electrode design in both architecture and active site levels for highly efficient HER/OER electrocatalysis^17,59^. In contrast with low-dimensional nanocatalysts that are usually immobilized on conductive substrate by polymer binder, our monolithic nanoporous Co_3_Mo/Cu electrode not only simultaneously facilitates the electron transfer and the HO^−^/H_2_O mass transports of along the interconnective Cu ligaments and the interpenetrating channels, respectively, but raises the accessibility of intermetallic Co_3_Mo nanoparticles by making use of small nanopores^60^. Meanwhile, the seamless integration between the electroactive Co_3_Mo nanoparticles and the conductive Cu ligaments with a coherently interfacial structure minimizes their contact resistance and improves the electrochemical durability. The multifunctional electrode architecture enlists the constituent intermetallic Co_3_Mo nanoparticles to exhibit exceptional electrocatalysis towards the alkaline HER. Because of the ordered crystal structure, the intermetallic Co_3_Mo nanoparticles have the precise surfaces composed of regular Co and Mo atoms, wherein their dissimilarity results in both ligand effect and strain effect to regulate the adsorption energies of HO* and H* intermediates^16^. In view that Mo is more oxophilic than Co, the HO* thermodynamically prefers to be adsorbed at the Co-Co-Mo hollow sites with a coverage of 1/2. This unique surface structure benefits for the subsequent adsorption/desorption of H* intermediates at the hollow sites nearby the hydroxylated Co–Co–Mo ones as a result of their optimum HBE and thermoneutral Δ*G*_H*_^16,25^. While triggered by electro-oxidation, the hydroxylated Co_3_Mo evolves into the Co_3_O_4_ nanoflakes with the in-situ incorporation of Mo, which improves the electronic conductivity of Co_3_O_4_ and adjusts the adsorption energy of HO*, remarkably boosting the OER electrocatalysis. The versatile properties of nanoporous Co_3_Mo/Cu electrode demonstrate the feasibility of dealloying quasi-eutectic multiphase alloys as a facile and scalable strategy to develop high-performance intermetallic compound-based electrocatalysts for the HER/OER.

In summary, we have developed nanoporous Cu supported intermetallic Co_3_Mo as self-supported monolithic electrocatalytic materials for highly efficient electrochemical splitting water. As a consequence of the intermetallic Co_3_Mo having exceptionally high intrinsic activity and the hierarchical nanoporous Cu skeleton offering fast electron-transfer and mass-transport pathways, the nanoporous Co_3_Mo/Cu electrodes exhibit negligible onset overpotential, and low Tafel slope of 40 mV dec^−1^, with an excellent durability. They only take the overpotential of as low as 96 mV to reach −400 mA cm^−2^. As a high-performance HER catalysts, the nanoporous Co_3_Mo/Cu electrode is coupled with its electro-oxidized derivative, i.e., EO Co_3_Mo/Cu, to construct an alkaline electrolyzer for the overall water splitting reaction. This alkaline electrolyzer delivers ~145 mA cm^−2^ at a low overpotential of 420 mV, along with long-term stability in the electrolyte of 1 M KOH and 0.5 M NaCl. The outstanding electrochemical performance enlists them to be promising candidates as low-cost anode and cathode materials for wide applications in water splitting.

## Methods

### Preparation of nanoporous electrocatalysts

All nanoporous hybrid electrodes were prepared by a scalable alloying/dealloying procedure. Precursor alloys, i.e., Cu_12−*x*−*y*_Co_*x*_Mo_*y*_Al_88_ (*x* = 0 or 3, *y* = 0 or 1) (at%) were firstly produced by arc melting pure Cu, Al metals with/without the addition of Co and Mo in an argon atmosphere. Nanoporous hybrid electrodes were fabricated by chemically etching their precursor alloy sheets with thickness of ∼400 μm in a N_2_-purged 6 M KOH electrolyte at 70 °C for 3 h, followed by a thorough rinse in ultrapure water (18 MΩ). To certify the electroactive sites, the nanoporous Co_3_Mo/Cu and bare Cu electrodes were contrastively treated in a N_2_-purged 0.5 M H_2_SO_4_ electrolyte at 70 °C for 30 min. The OER electrodes were prepared by electrochemically oxidizing the as-dealloyed nanoporous Cu-based electrodes in 1 M KOH at 1.57 V vs. RHE for 20 min. Pt/C and Ir/C catalyst inks were prepared by mixing commercially available Pt/C (20 wt%, Johnson Matthey) and Ir/C (5 wt%, Macklin) in a Nafion (0.05 wt%, Sigma Aldrich) solution containing isopropanol (20 %) and water (80 %) under rigorous sonication, respectively. Total 100 μL Pt/C and Ir/C inks were drop-cast onto nanoporous Cu electrodes (2 mm × 5 mm × 0.4 mm) to prepare the Pt/C/Cu and Ir/C/Cu electrodes for electrochemical measurements.

### Structural characterizations

Microstructure characterizations and chemical component analysis of nanoporous electrocatalyst electrodes were performed on a field-emission scanning electron microscope (JSM-6700F, JEOL, 7 keV) equipped with X-ray energy-dispersive spectroscopy (EDS), and a field-emission transition electron microscope (JEM-ARM300F, JEOL) equipped with double spherical-aberration correctors for both condenser and objective lens, respectively. XRD measurements of nanoporous electrocatalyst electrodes were conducted on a D/max2500pc diffractometer with a monochromated Cu *K*_α_ radiation. Chemical states and distribution of surface elements were analyzed using XPS and LEIS on a Thermo ECSALAB 250 with an Al anode. Charging effects were compensated by shifting binding energies according to the C 1 s peak (284.8 eV). Inductively coupled plasma optical emission spectrometry (ICP-OES, Thermo electron) analysis was conducted to determine the concentrations of metal ions. Raman spectra were measured on a micro-Raman spectrometer (Renishaw) equipped with 532 nm-wavelength laser at a power of 0.5 mW.

### Electrochemical measurements

Electrochemical measurements were performed in a classic three-electrode system with a graphite rod as the counter electrode, an Ag/AgCl electrode as the reference electrode, and self-supported HER and OER electrocatalytic materials as the working electrodes. The HER and OER polarization curves of nanoporous electrocatalytic materials were measured in a N_2_-saturated and O_2_-saturated 1 M KOH aqueous solution (pH = 13.8) at 25 °C, respectively. The scan rate is 1 mV s^−1^. The potential calibration experiment was carried out using a Pt wire as the working electrode in a H_2_-saturated electrolyte. Therein, the current-potential curve was collected at a scan rate of 1 mV s^−1^, and the potential with *i* = 0 was considered as the thermodynamic potential for the hydrogen electrode reaction. All the potentials were calibrated with respect to RHE and iR-corrected according to the equations, *E*_RHE_ = *E*_Ag/AgCl_ + 1.016 V, and *E*_RHE_ = *E*_Ag/AgCl_ + 1.016 V – iR, respectively. EIS analysis was conducted at overpotential of 100 mV with frequency ranging from 10 mHz to 100 kHz. Cyclic voltammogram (CV) curves in the voltage window from −0.95 to −0.85 V (vs. Ag/AgCl) were collected for all nanoporous electrocatalytic materials at various scan rates, according to which their double-layer capacitance (*C*_dl_) values were determined and used to evaluate the ECSAs. The HER durability tests of nanoporous Co_3_Mo/Cu electrode were performed at −60 and −80 mV vs. RHE in 1 M KOH for 1000 and 500 h, respectively. While the OER durability measurement of EO Co_3_Mo/Cu electrode was carried out at the overpotential of 300, 350, and 400 mV in 1 M KOH, respectively, for 10 h. As for the overall-splitting water, a two-electrode system was used to assess the electrochemical performance in a brine electrolyte (1 M KOH and 0.5 M NaCl), wherein the stability test was carried out at 1.65 V for 30 h.

### DFT simulation

DFT calculations were performed on five-layer slabs of Co_3_Mo(002), Cu(111), Mo(110), and Co(001), using the Vienna ab initio simulation package (VASP)^61,62^. The exchange-correlation potential was determined by the meta-GGA with SCAN functional^63^, according to which the geometries and energies of various materials and molecules can be accurately predicted^64^. The interactions between electrons and ions were described by the scalar relativistic all-electron Blöchl's projector augmented-wave method^65^. To consider the effect of the aqueous solvent environment on the energies, the solvent model that treats the electrolyte at the solid-liquid interface as a polarizable continuum and places point counter-charges via the linearized Poisson–Boltzmann equation was employed in all DFT calculations by using VASPsol code^66^. During the geometry optimization, the atoms of the bottom two layers were fixed, and all other atoms were fully relaxed. In our prudently convergence tests, the plane wave-basis expansion cutoff energy was set to 400 eV, and the Brillouin Zone was sampled with 7 × 7 × 1 Monkhorst–Pack K-point mesh. The convergence criterions of electronic structure and atomic geometry structure were 1 × 10^−4^ eV, 0.02 eV/Å, respectively.

## Supplementary information

Supporting Information

Peer Review File

## Data Availability

All relevant data are available from the corresponding authors upon request.
