# Supplementary material for: Spontaneously separated intermetallic Co3Mo from nanoporous copper as versatile electrocatalysts for highly efficient water splitting
Source: Nat Commun. 2020 Jun 10;11:2940. doi: 10.1038/s41467-020-16769-6 (PMC7287083; doi:10.1038/s41467-020-16769-6)
Supplement: Supplementary file 1 — Supporting Information [file 41467_2020_16769_MOESM1_ESM.pdf]

Supplementary information

Spontaneously separated intermetallic Co_3Mo from nanoporous copper as versatile electrocatalysts for highly efficient water splitting

Shi et al

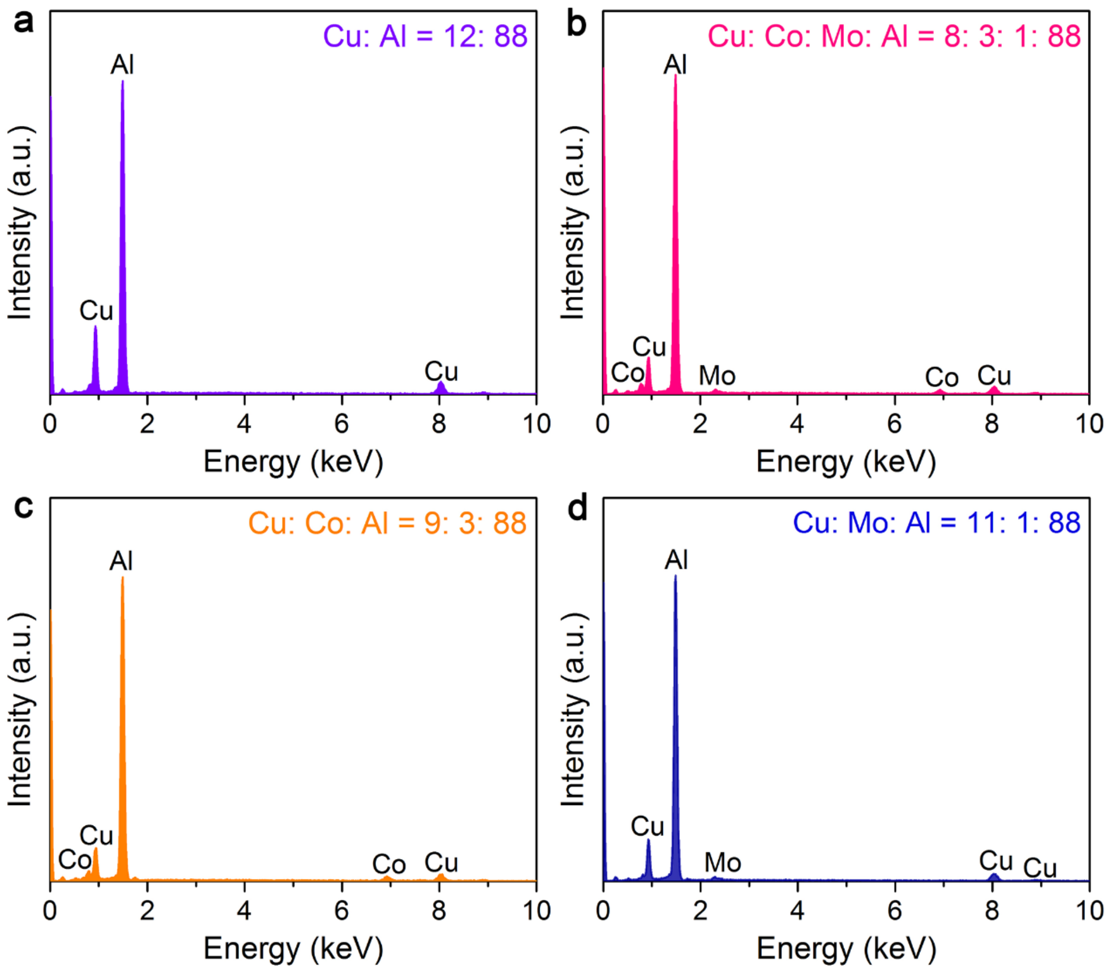

Supplementary Figure 1. **EDS elemental characterizations of precursor alloys.** EDS spectra of precursor alloys of $Cu_{12}Al_{88}$ (a), $Cu_8Co_3Mo_1Al_{88}$ (b), $Cu_{11}Mo_1Al_{88}$ (c) and $Cu_9Co_3Al_{88}$ (d) alloys.

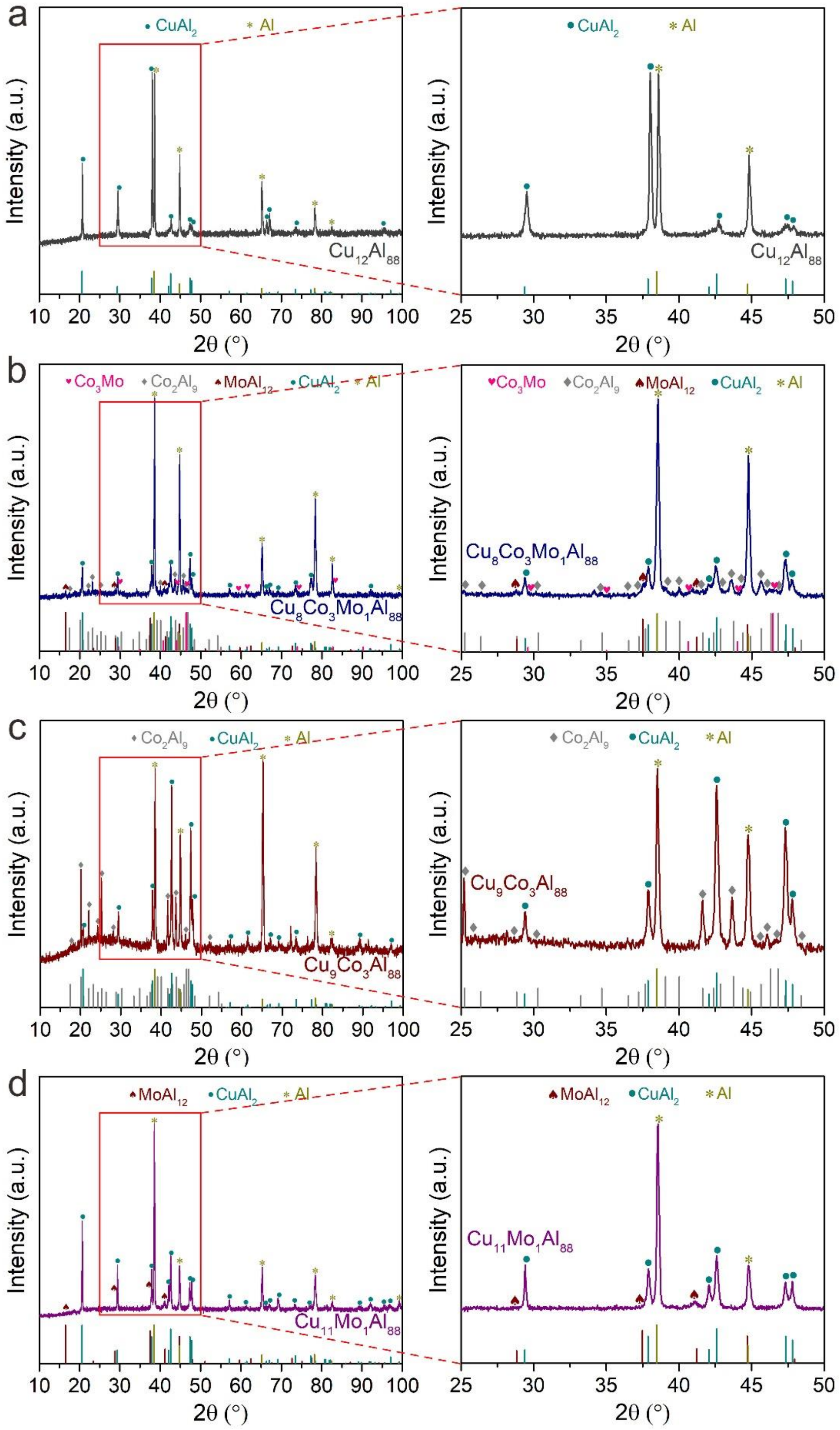

Supplementary Figure 2. **XRD characterizations of precursor alloys. a-d,** XRD patterns of precursor $Cu_{12}Al_{88}$ (a), $Cu_8Co_3Mo_1Al_{88}$ (b), $Cu_9Co_3Al_{88}$ (c) and $Cu_{11}Mo_1Al_{88}$ (d) alloys. The line patterns show reference cards 25-0012 for $CuAl_2$

(dark cyan line), 04-0787 for α-Al (dark yellow line), 25-0488 for Co_3Mo (pink line), 06-0699 for Co_2Al_9 (gray line), and 29-0052 for $MoAl_{12}$ (wine line) according to JCPDS.[1]

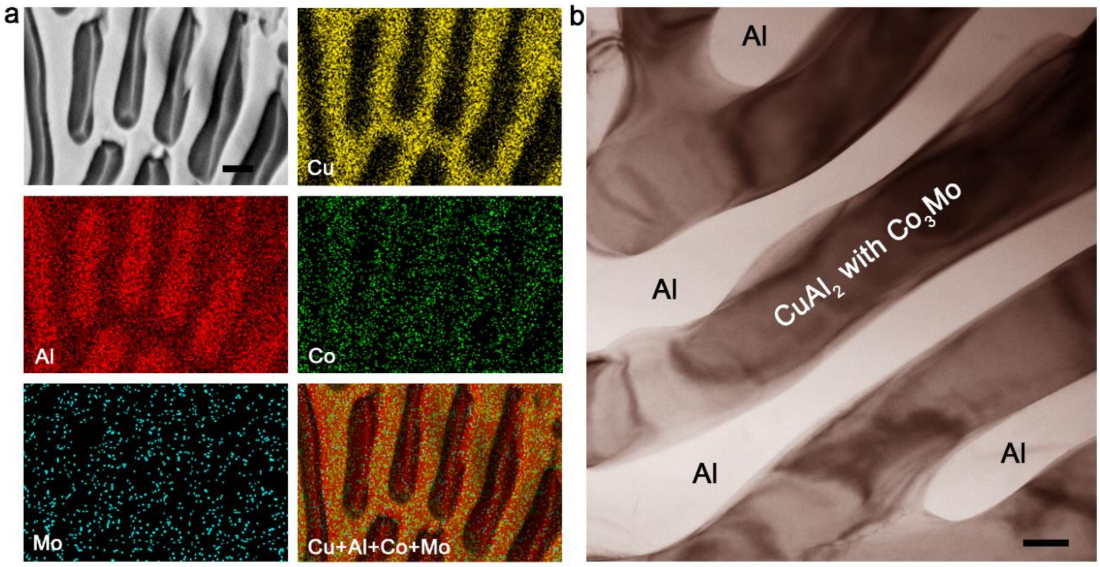

Supplementary Figure 3. **Microstructure characterization of representative precursor $Cu_8Co_3Mo_1Al_{88}$ alloy. a**, SEM backscattered electron image of $Cu_8Co_3Mo_1Al_{88}$ alloy and the corresponding SEM-EDS mapping of Cu, Al, Co, Mo elements, which are in yellow, red, green and blue, respectively. Scale bar: 500 nm. **b**, TEM image of $Cu_8Co_3Mo_1Al_{88}$ alloy. Scale bar: 200 nm.

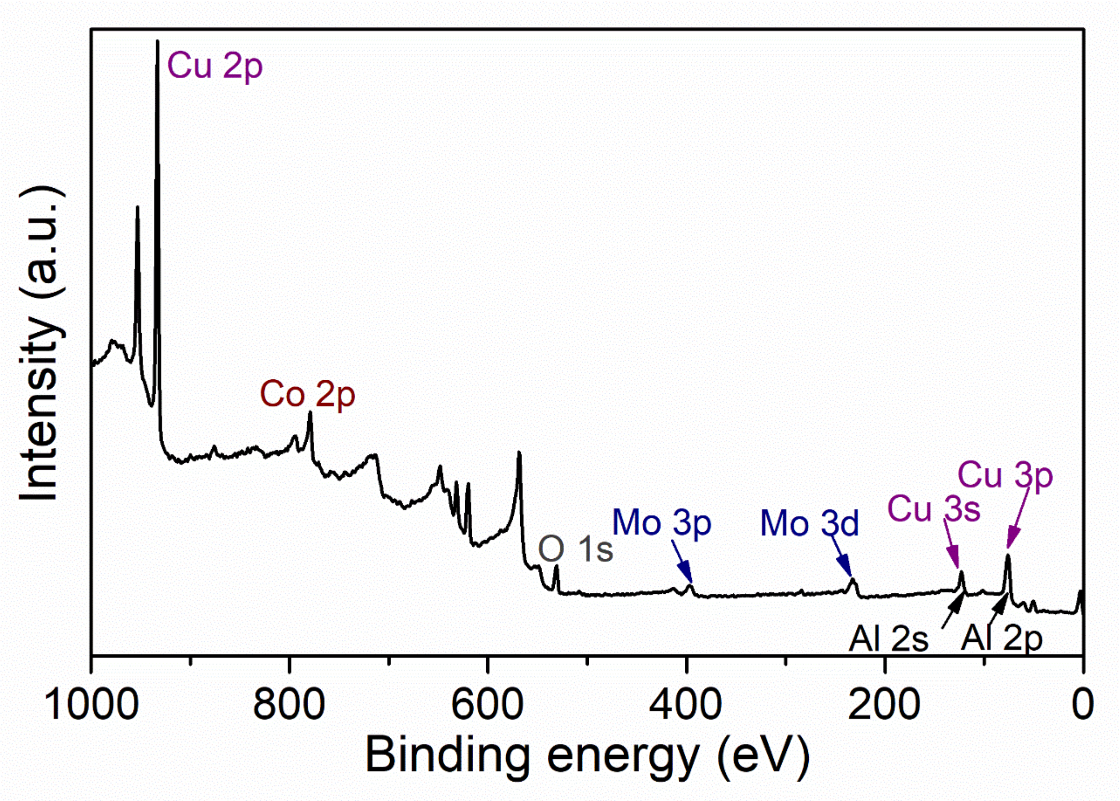

Supplementary Figure 4. XPS survey of as-dealloyed nanoporous Co_3Mo/Cu electrode.

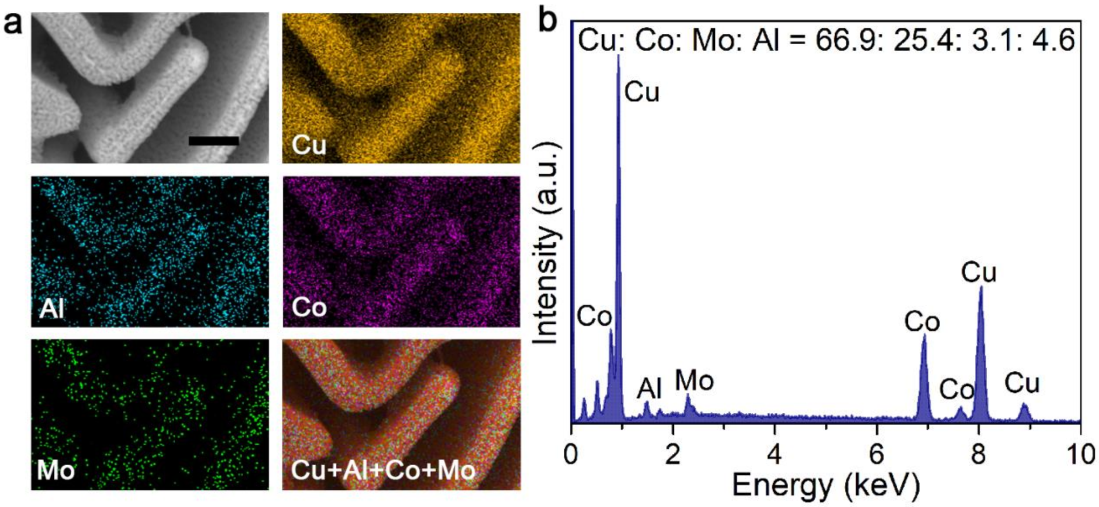

Supplementary Figure 5. Chemical characterization of nanoporous Co_3Mo/Cu. a, Representative SEM backscattered electron image and the corresponding EDS mapping of Cu, Al, Co and Mo elements for the as-prepared nanoporous Co_3Mo/Cu electrode from the $Cu_8Co_3Mo_1Al_{88}$ alloy precursor. Scale bar: 500 nm. **b**, EDS spectrum of the as-prepared nanoporous Co_3Mo/Cu electrode from the $Cu_8Co_3Mo_1Al_{88}$ alloy precursor.

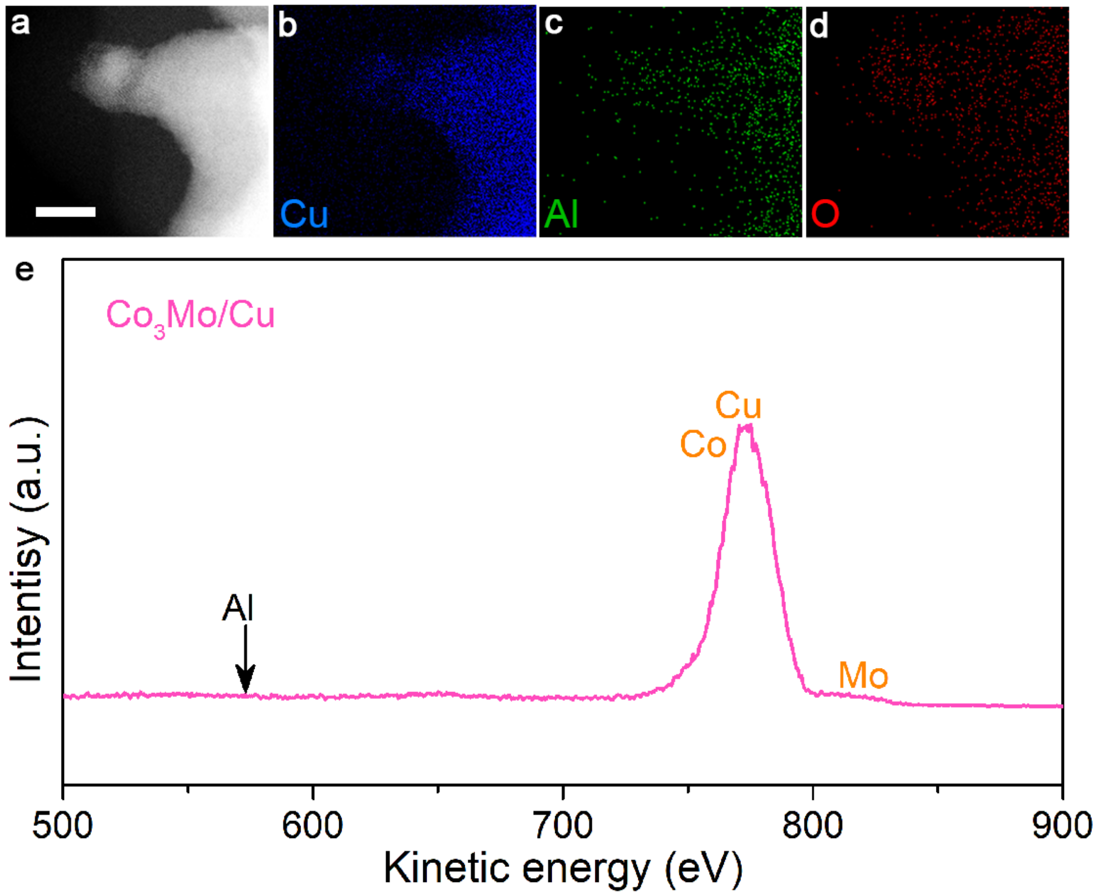

Supplementary Figure 6. Chemical characterization of nanoporous Co_3Mo/Cu. a, Representative STEM image of the as-prepared nanoporous Co_3Mo/Cu electrode. **b,c,d**, The corresponding EDS mapping of Cu (b), Al (c) and O (d) elements. Scale bar: 10 nm. **e**, Low-energy ion scattering (LEIS) spectrum of nanoporous Co_3Mo/Cu taken with He ions of 1 keV. The absence of Al demonstrates that there are only Co and Mo, Cu on the surface.

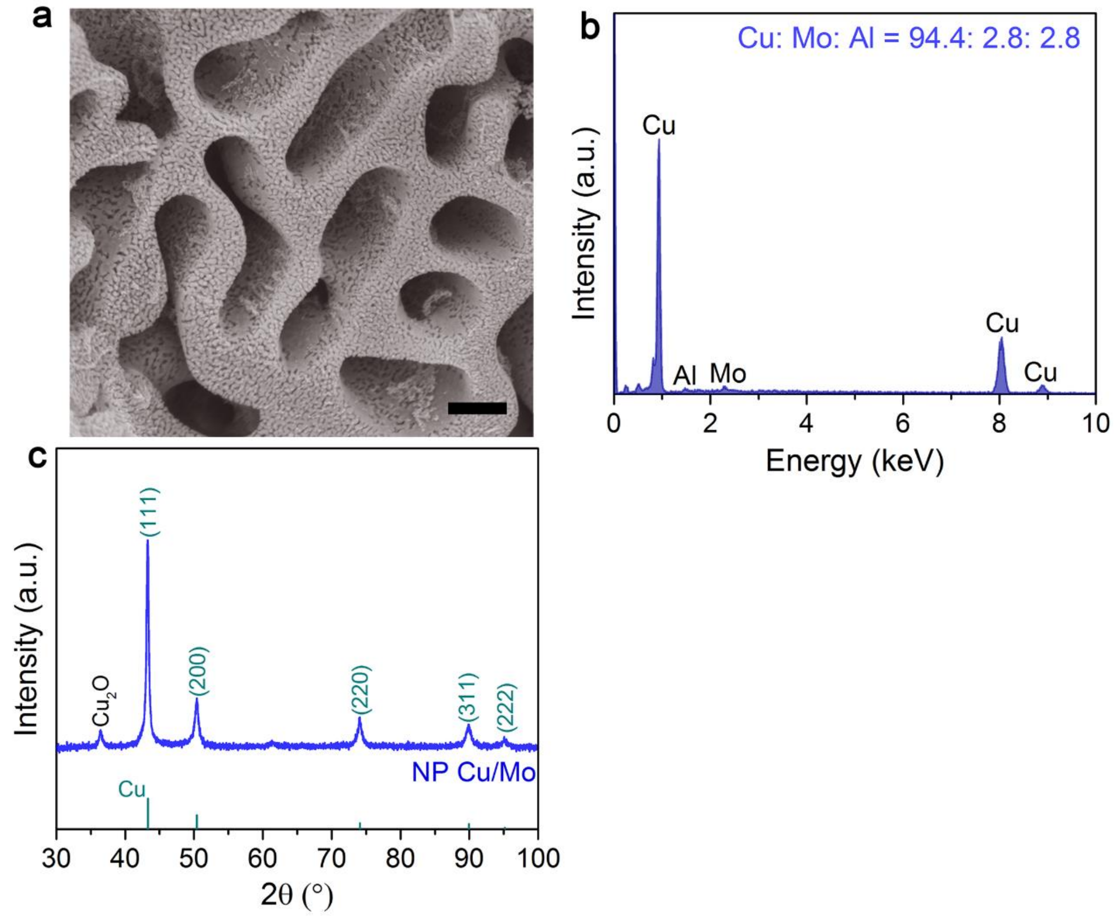

Supplementary Figure 7. **Structure characterization of nanoporous Mo/Cu. a**, Typical cross-sectional SEM image of nanoporous Mo/Cu. Scale bar: 500 nm. **b**, EDS spectrum of nanoporous Mo/Cu. **c**, XRD patterns of nanoporous Mo/Cu. The line patterns show reference card 04-0836 for Cu according to JCPDS.

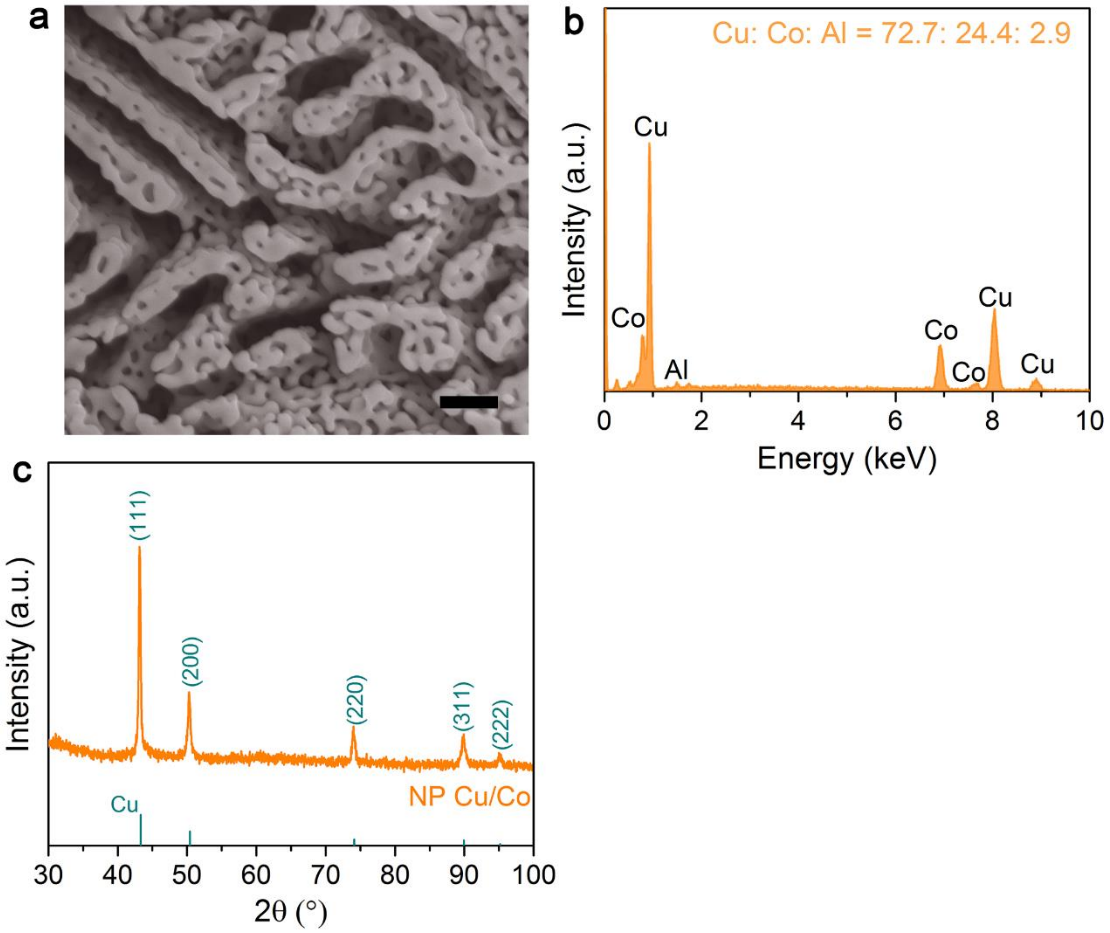

Supplementary Figure 8. **Structure characterization of nanoporous Co/Cu. a**, Typical cross-sectional SEM image of nanoporous Co/Cu. Scale bar: 500 nm. **b**, EDS spectrum of nanoporous Co/Cu. **c**, XRD patterns of nanoporous Co/Cu. The line patterns show reference card 04-0836 for Cu according to JCPDS.

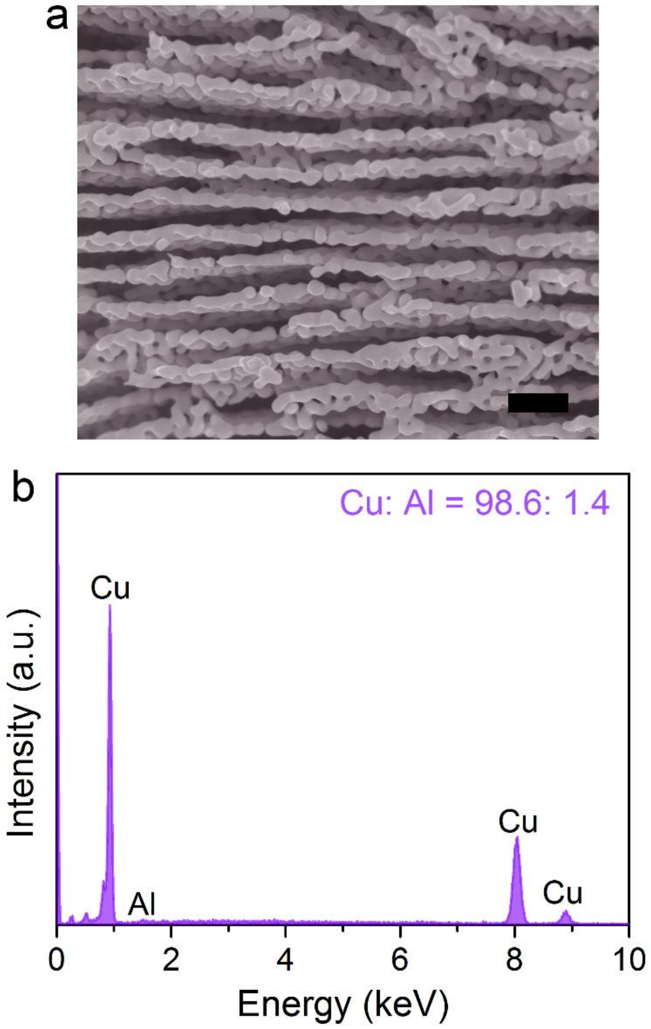

Supplementary Figure 9. **Structure characterization of nanoporous Cu. a**, Typical cross-sectional SEM image of nanoporous Cu. Scale bar: 500 nm. **b**, EDS spectrum of nanoporous Cu.

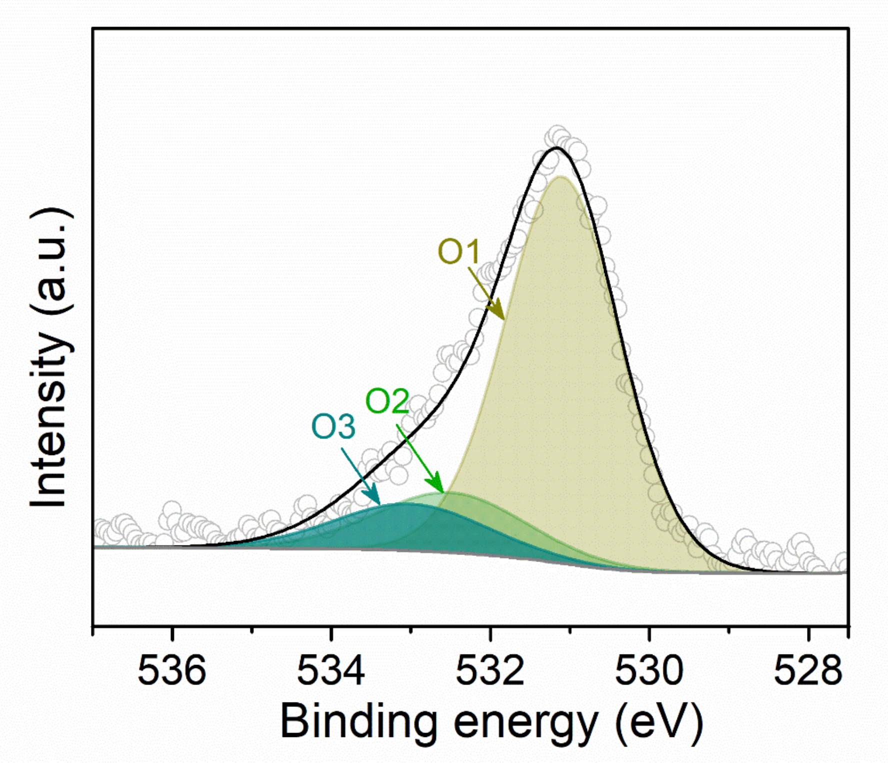

Supplementary Figure 10. High-resolution XPS spectrum of O 1s in the nanoporous Co_3Mo/Cu electrode. Therein, the O 1s spectrum can be deconvolated into three characteristic peaks of oxygen atoms in H-O bond (531.2 eV, O1), surface-adsorbed oxygen (532.6 eV, O2) and adsorbed molecular water (533.4 eV, O3).

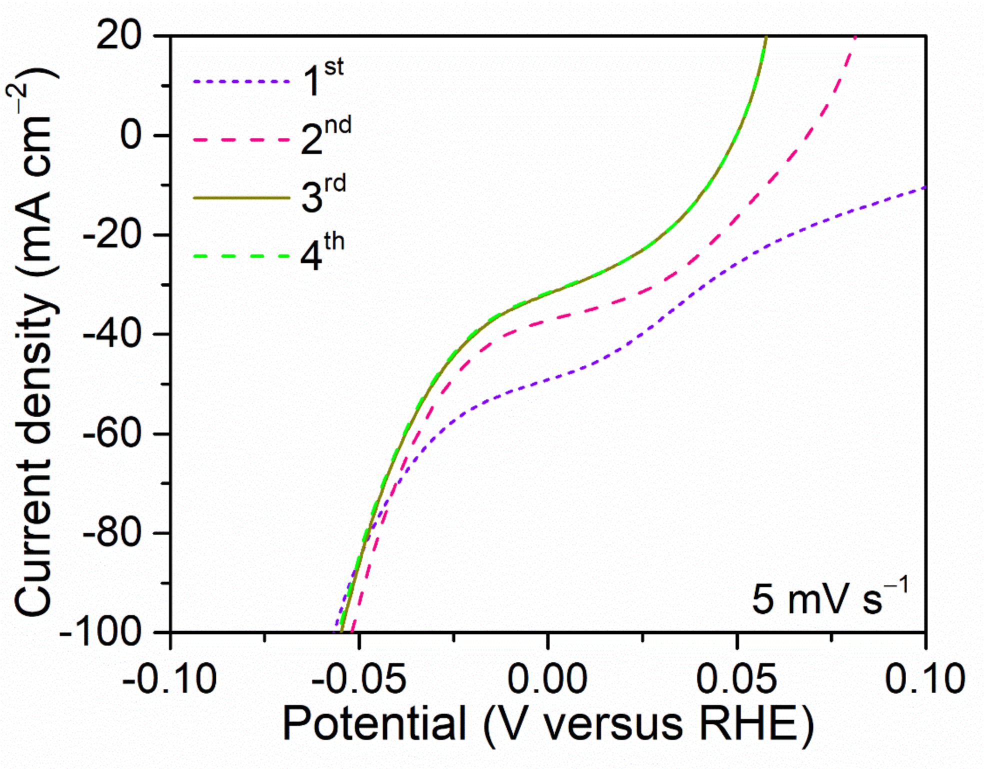

Supplementary Figure 11. Initial four LSV polarization curves of nanoporous Co_3Mo/Cu electrode. Scan rate: 5 mV s^{-1}; Electrolyte: 1 M KOH.

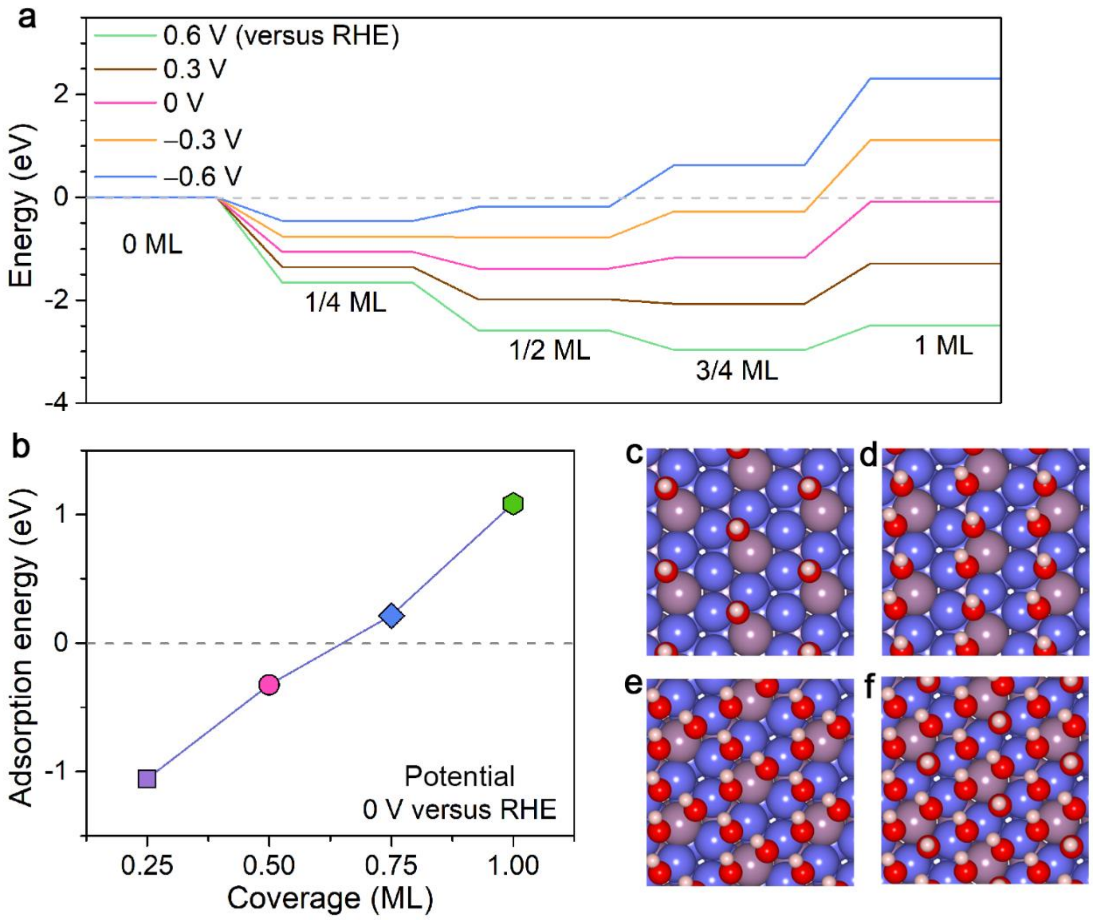

Supplementary Figure 12. **a**, Relative energies of *OH adsorption on Co_3Mo(002) at different voltages of −0.6, −0.3, 0, 0.3 and 0.6 V versus RHE. **b**, Adsorption energy of *OH on the Co_3Mo(002) surface at the potential of 0 V versus RHE. **c-f**, The corresponding atomic structures of Co_3Mo(002) surface with the *OH adsorption coverage of 1/4 ML (b), 1/2 ML (c), 3/4 ML (d) and 1 ML (e), respectively.

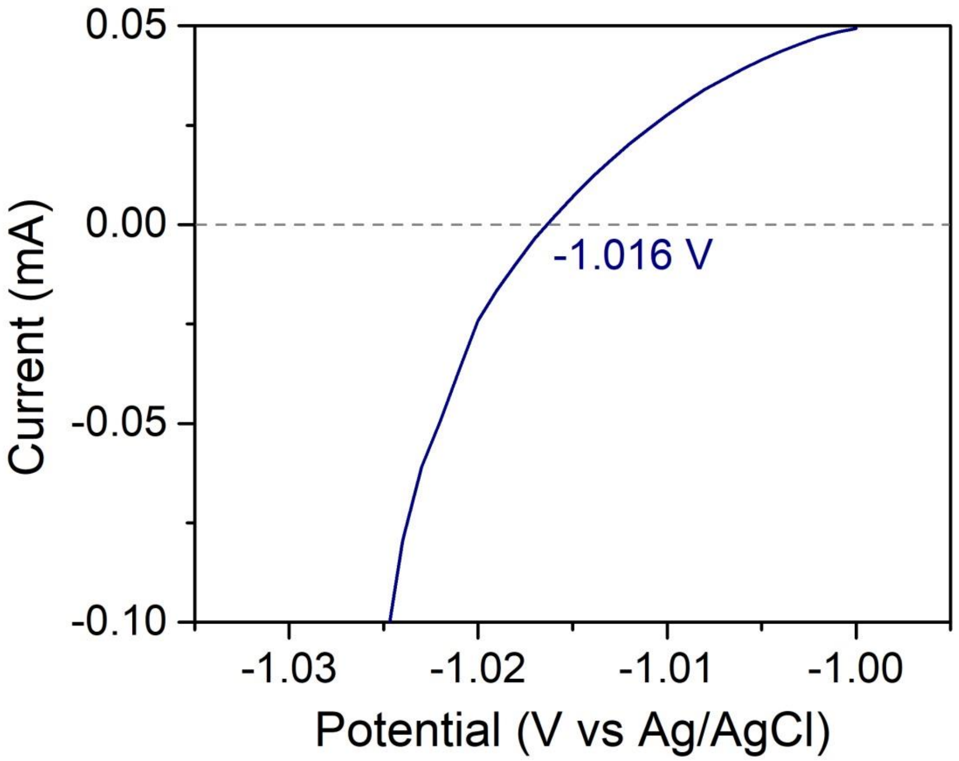

Supplementary Figure 13. Current-potential curve of Pt wire as the working electrode in H_2-saturated 1 M KOH aqueous solution to calibrate the Ag/AgCl electrode with respect to RHE, $E_{RHE} = E_{Ag/AgCl} + 1.016$ V. Scan rate: 1 mV s^{-1}.

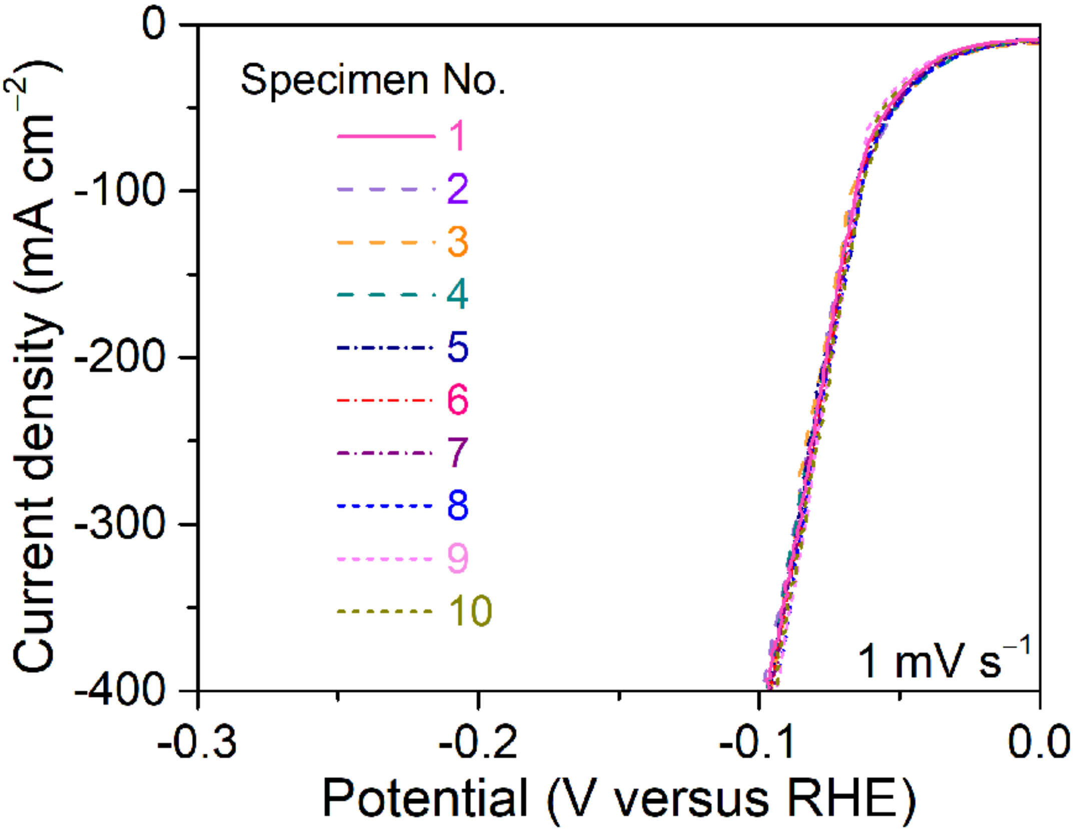

Supplementary Figure 14. Reproducibility. The HER polarization curves for ten nanoporous Co_3Mo/Cu electrodes, which are prepared by chemically dealloying the precursor $Cu_8Co_3Mo_1Al_{88}$ alloy. The aqueous electrolyte of HER measurements: 1 M KOH; scan rate: 1 mV s^{-1}.

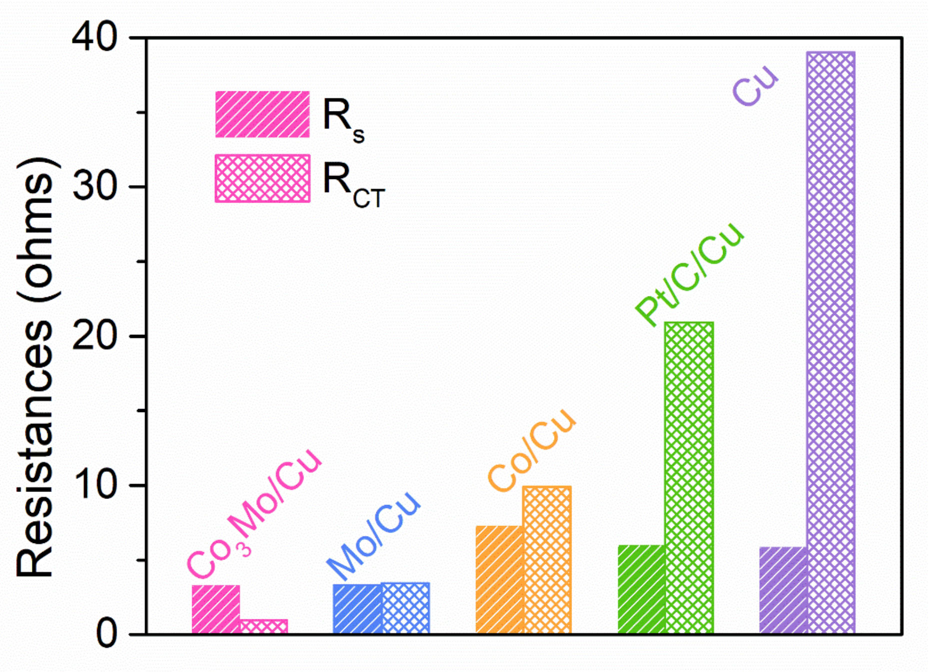

Supplementary Figure 15. Comparison of intrinsic resistance and charge transfer resistance of nanoporous Co_3Mo/Cu, Mo/Cu, Co/Cu, Cu and nanoporous Pt/C/Cu according to EIS analysis in 1 M KOH.

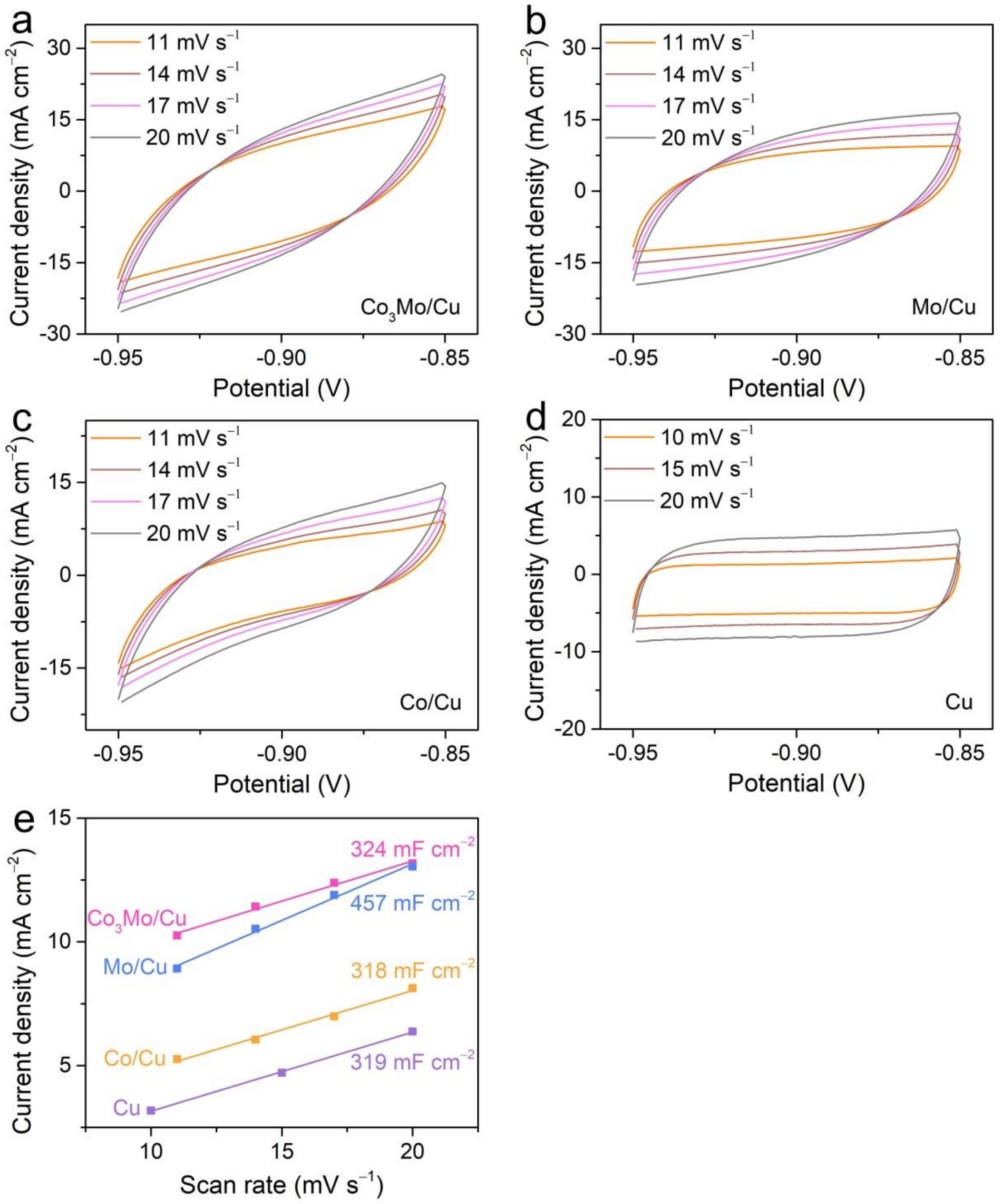

Supplementary Figure 16. Electrochemical capacitance measurements. a-d, Cyclic voltammograms in the potential windows ranging from -0.95 V to 0.85 V (vs Ag/AgCl) for nanoporous Co_3Mo/Cu (a), nanoporous Mo/Cu (b), nanoporous Co/Cu (c) and nanoporous Cu (d) at various scan rates. **e**, The double-layer capacitances (C_{dl}) calculated by the differences in current densities at -0.90V (vs Ag/AgCl) as a linear function of scan rates.

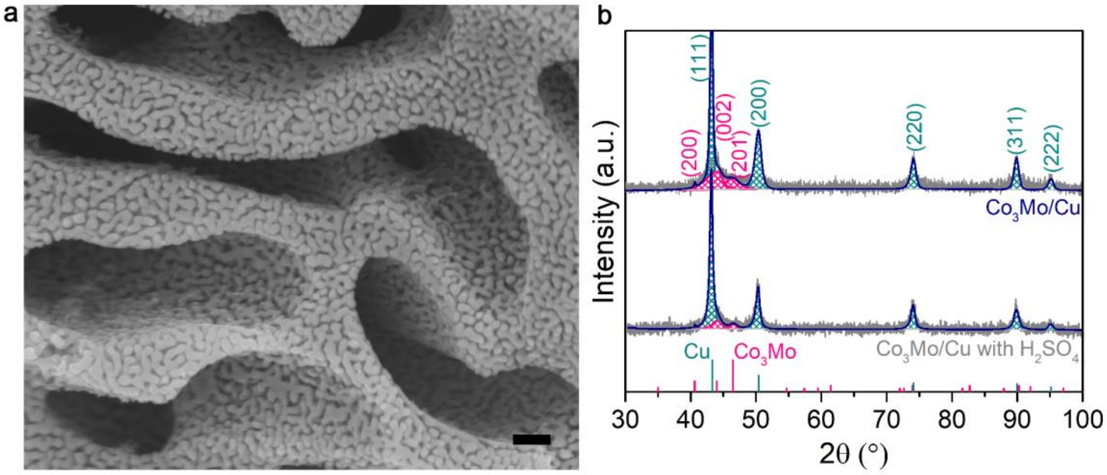

Supplementary Figure 17. Structure characterization of H_2SO_4-treated nanoporous Co_3Mo/Cu. a, Typical cross-sectional SEM image of H_2SO_4-treated nanoporous Co_3Mo/Cu. Scale bar: 200 nm. **b,** XRD patterns of H_2SO_4-treated nanoporous Co_3Mo/Cu, comparing with the ones of the pristine nanoporous Co_3Mo/Cu.

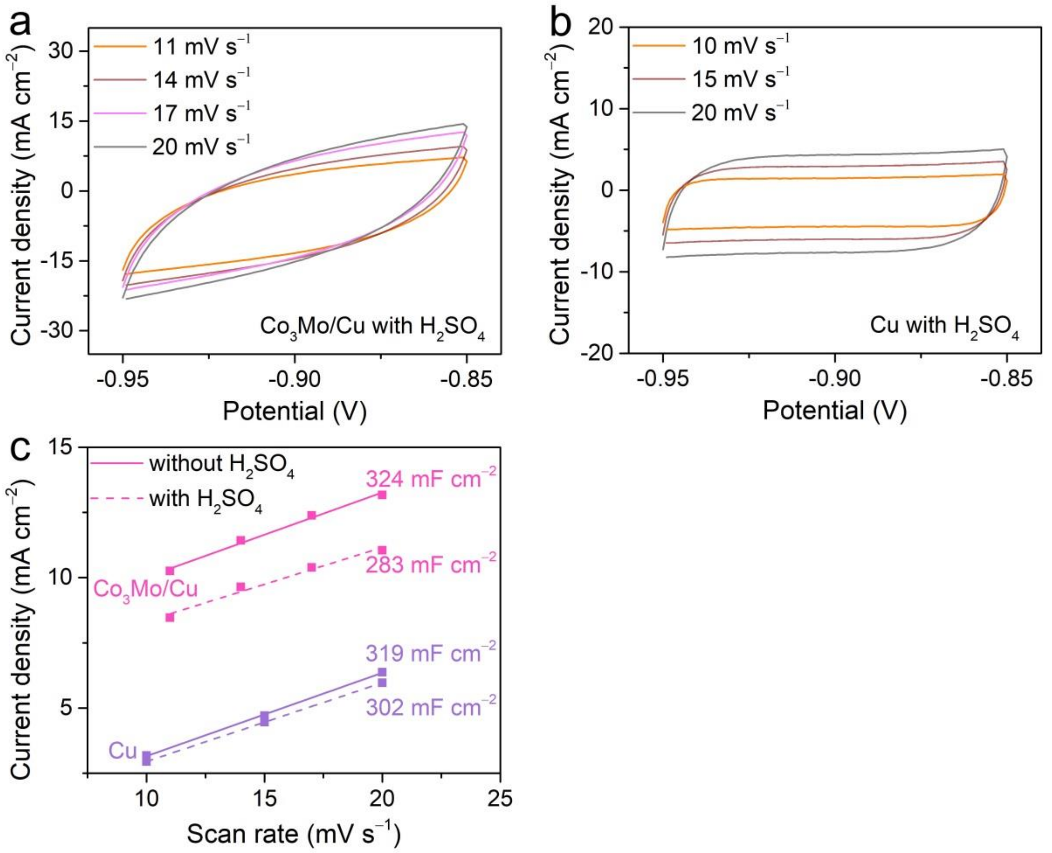

Supplementary Figure 18. Electrochemical capacitance measurements. a,b, CV curves in the potential windows ranging from -0.95 V to 0.85 V (vs Ag/AgCl) for H_2SO_4-treated Co_3Mo/Cu (a) and Cu (b) at various scan rates. **c**, The double-layer capacitances (C_{dl}) calculated by the differences in current densities at -0.90V (vs Ag/AgCl) as a linear function of scan rates.

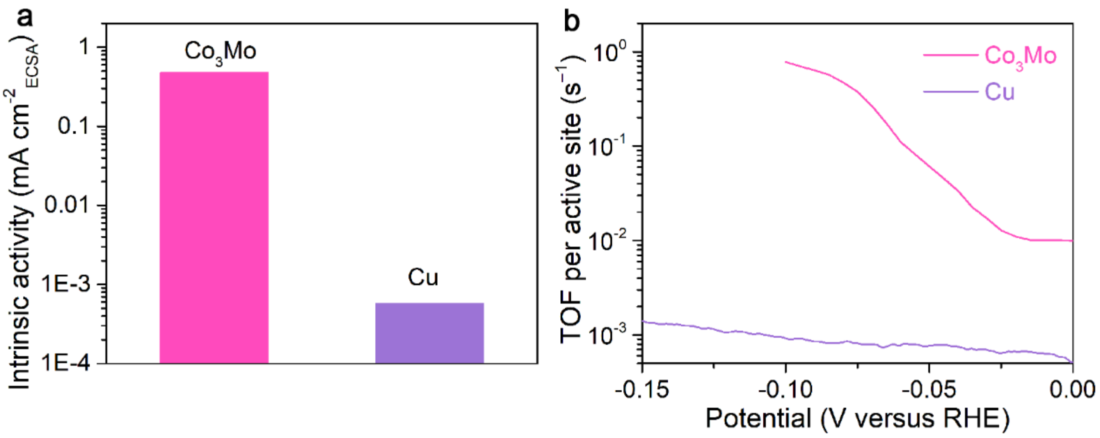

Supplementary Figure 19. Intrinsic activity and TOF value. a, Intrinsic activities of Co_3Mo and bare Cu at the overpotential of 100 mV. **b**, TOF values as a function of potential for Co_3Mo and bare Cu.

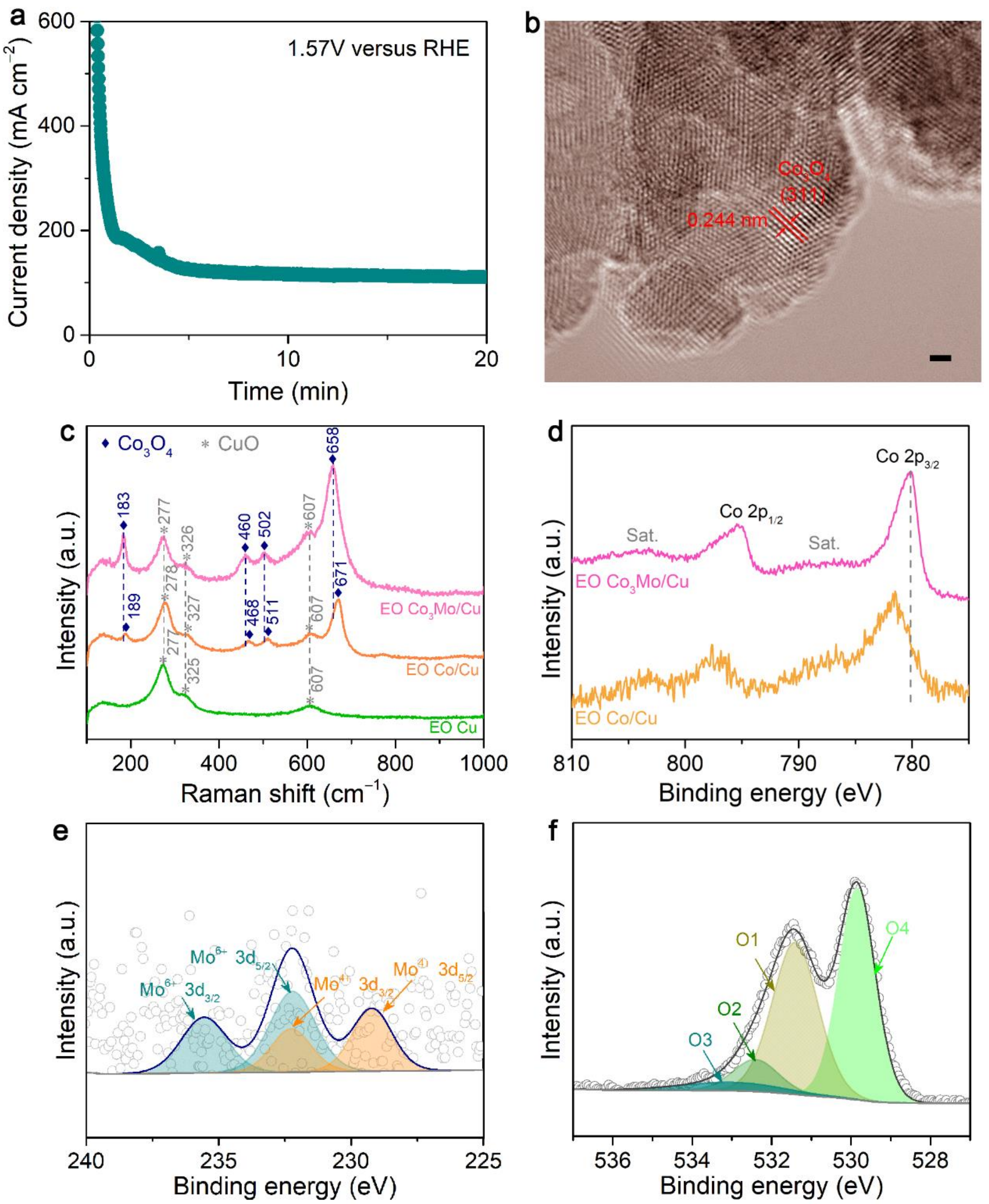

Supplementary Figure 20. Preparation and characterization of electro-oxidized nanoporous Co_3Mo/Cu electrode. a, A typical *I-t* curve at a potential of 1.57 V versus RHE in 1 M KOH electrolyte, illustrating the fabrication process of electro-oxidation of nanoporous Co_3Mo/Cu. **b**, Typical HRTEM image of Mo-Co_3O_4 nanoflakes that grow on CuO/Cu skeleton during the electro-oxidation of nanoporous Co_3Mo/Cu. Scale bar: 1 nm. **c**, Raman spectra of electro-oxidized nanoporous Cu (EO Cu), nanoporous Co_3Mo/Cu (EO Co_3Mo/Cu) and nanoporous Co/Cu (EO Co/Cu). **d**, High-resolution Co 2p XPS spectra of EO Co_3Mo/Cu and EO Co/Cu, which are

prepared by electro-oxidation of nanoporous Co_3Mo/Cu and Co/Cu electrodes, respectively. **e**, **f**, High-resolution Mo 3d (e) and O 1s (f) XPS spectra of EO Co_3Mo/Cu electrode. Therein, the O 1s spectrum can be deconvoluted into three characteristic peaks of oxygen atoms in H-O bond (531.2 eV, O1), surface-adsorbed oxygen (532.6 eV, O2), adsorbed molecular water (533.4 eV, O3) and oxygen atoms bound to metals (529.8 eV, O4).

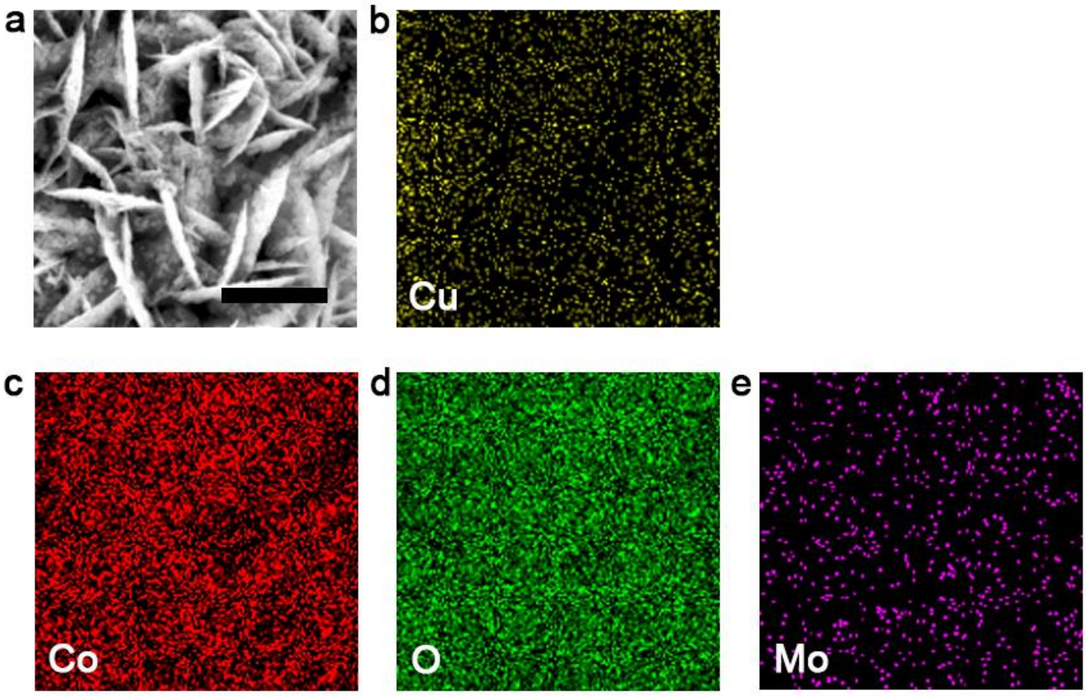

Supplementary Figure 21. SEM-EDS mapping of electro-oxidized nanoporous Co_3Mo/Cu electrode before electrochemical measurement. a, Typical SEM image of electro-oxidized nanoporous Co_3Mo/Cu electrode. Scan bar: 1 μm. **b-e**, The corresponding element distribution of Cu (b), Co (c), O (d) and Mo (e).

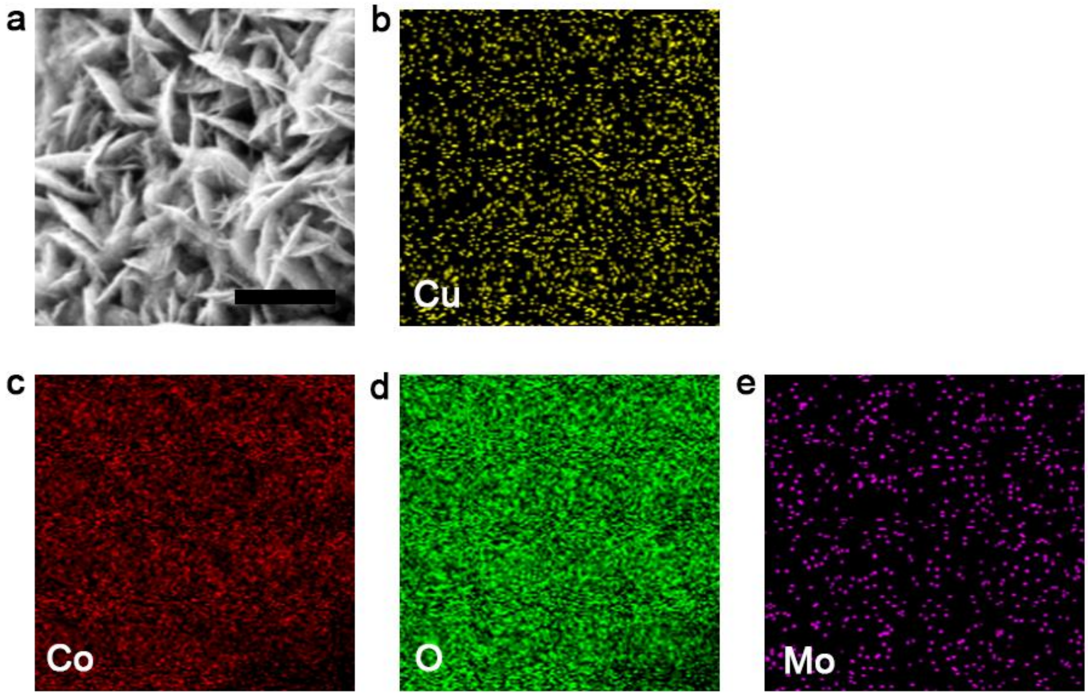

Supplementary Figure 22. SEM-EDS mapping of electro-oxidized nanoporous Co_3Mo/Cu electrode after electrochemical measurement. a, Typical SEM image of electro-oxidized nanoporous Co_3Mo/Cu electrode after electrochemical measurement. Scan bar: 1 μm. **b-e**, The corresponding element distribution of Cu (b), Co (c), O (d) and Mo (e).

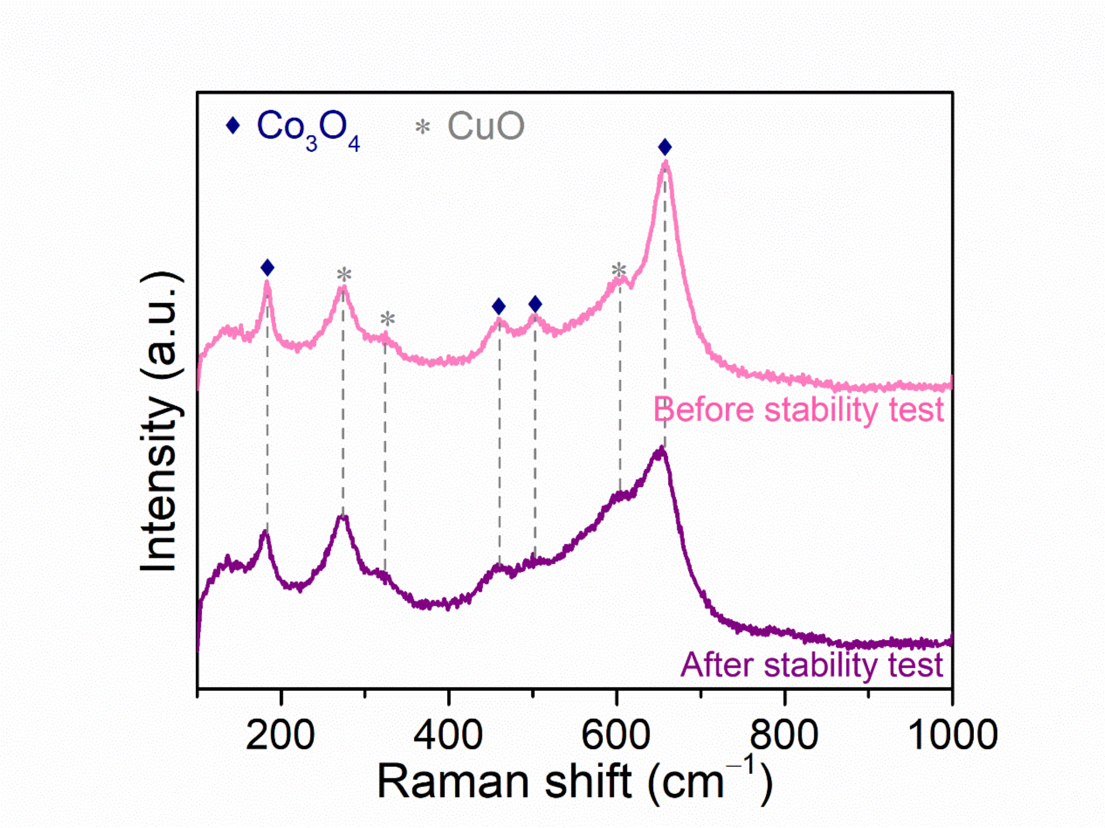

Supplementary Figure 23. Raman spectra of EO Co_3Mo/Cu electrode before and after durability test.

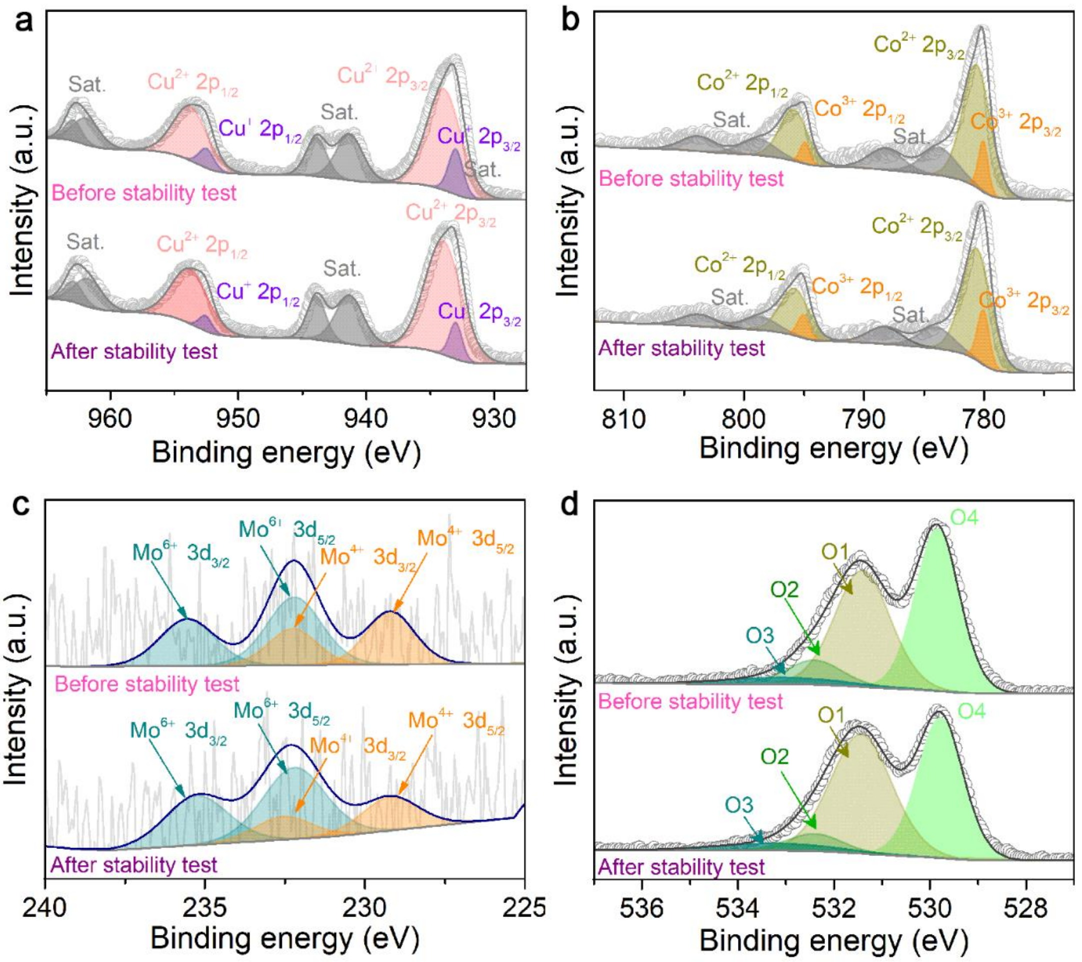

Supplementary Figure 24. XPS characterization of EO Co_3Mo/Cu electrode before and after durability test. a-d, Cu 2p (a), Co 2p (b), Mo 3d (c) and O 1s (d) XPS spectra of EO Co_3Mo/Cu electrode before and after durability test.

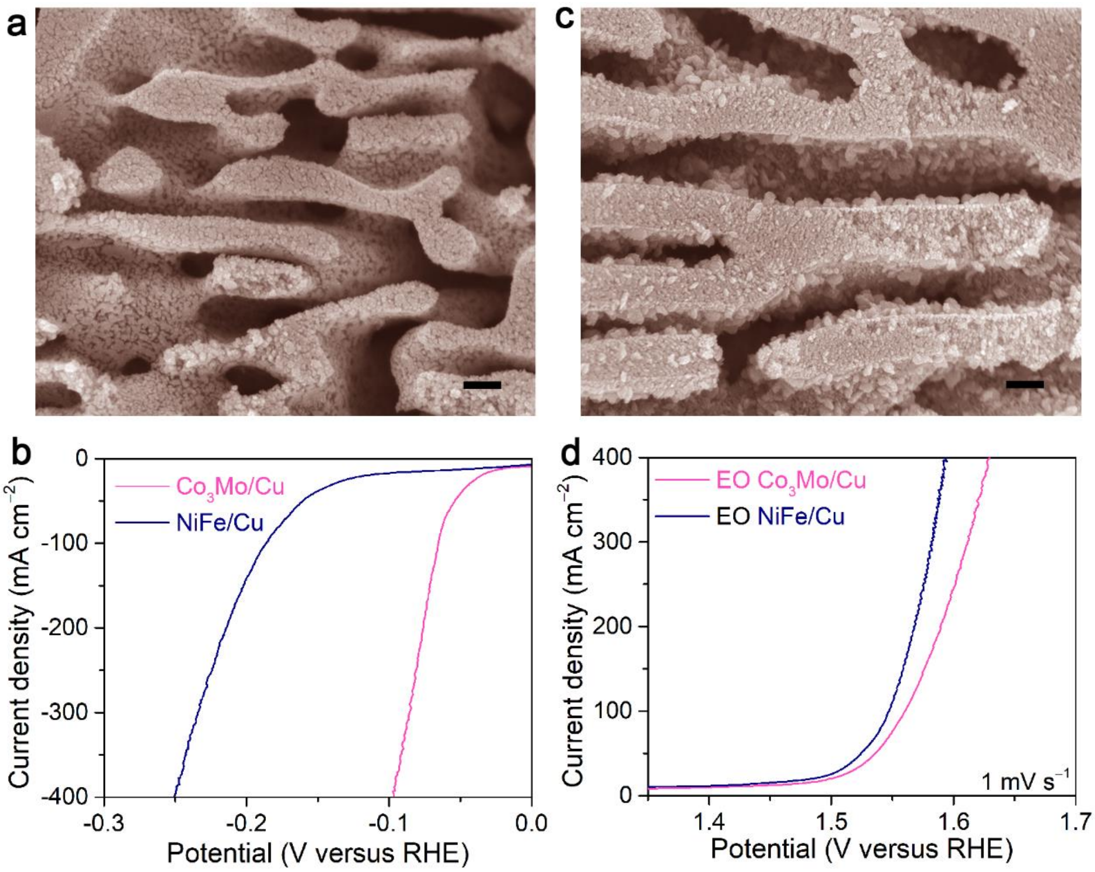

Supplementary Figure 25. Microstructure and electrochemical properties of nanoporous NiFe/Cu and electro-oxidized nanoporous NiFe/Cu electrodes. a, Typical SEM image of nanoporous NiFe/Cu electrode. Scale bar: 200 nm. **b** Comparison of HER polarization for nanoporous Co_3Mo/Cu and NiFe/Cu electrodes in 1 M KOH. Scale bar: 200 nm. **c**, Typical SEM image of electro-oxidized nanoporous NiFe/Cu electrode. **d**, Comparison of OER polarization for EO Co_3Mo/Cu and EO NiFe/Cu electrodes in 1 M KOH.

Supplementary Table 1. Comparisons of the TOF of electroactive Co_3Mo at the overpotential of 100 mV in 1 M KOH electrolyte with representative electrocatalysts reported previously.

Electrocatalysts	TOF (s^{-1})	Refs.
Co_3Mo/Cu	0.775	This work
$NiCo_2P_x$	0.056	2
Amidated carbon fiber	0.41	3
S-CoO	0.41	4
FeP/Ni_2P	0.163	5
N-$NiCo_2S_4$	0.35	6
MoS_2/NiCo-LDH	0.22	7
V-Co_4N	0.05	8

Supplementary Table 2. ICP-OES results of the electrolyte after the long-term durability measurements of nanoporous Co_3Mo/Cu electrodes at the overpotential of 60 and 80 mV in 1M KOH electrolyte for 1000 and 500 h.

	60 mV	80 mV
	Concentration (mg/L)	Concentration (mg/L)
Co	0	0
Mo	0	0
Cu	0.0090	0
Al	0.0485	0.0773

Supplementary Table 3. Comparisons of the HER catalytic activity of nanoporous Co_3Mo/Cu electrode in 1 M KOH electrolyte with representative electrocatalysts reported previously.

Electrocatalysts	Overpotentials (mV)	Current density (mA cm^{-2})	Tafel slopes (mV/dec)	Refs.
Co_3Mo/Cu	12	10	40	This work
	54	50		
	65	100		
	76	200		
	86	300		
	96	400		
NiCu@C	94	10	95	10
	123	50		
	247	100		
Cu/Ni_3S_2	128	10	76	11
	210	50		
	268	100		
Cu_1Ni_2-N	71	10	107	12
	200	100		
	255	200		
Ni_5Co_3Mo-OH	52	10	59	13
	250	100		
	290	200		
Cu_3N	118	10	122	14
	280	100		
	335	200		
	375	300		
NiMoN/CFC	40	10	70	15
	260	100		
	400	200		
Ni/γ-Fe_2O_3	46	10	58	16
	150	100		
Pt/CoSe	58	10	39	17
	170	100		
Co_3Mo/MoO_x/Ni	68	10	61	18
	170	100		
	200	200		
	225	300		
MoS_2/Mo_2C	120	10	43	19
	140	100		
	155	200		

	180	400		
NiMoN/Ni	-	10	46	20
	56	100		
	78	200		
	115	400		
o-$CoSe_2P$	104	10	69	21
	180	100		
	210	300		
Ni-FeP/C	95	10	72	22
	175	100		
Ni_5P_4-Ru	54	10	60	23
	120	100		
P-Fe_3O_4	-	10	42	24
	138	100		
	175	200		
	210	400		
$NiFeO_x$@NiCu	70	10	68	25
	210	100		
Co_3S_4/MOF	80	10	82	26
	180	100		
CoFeZr oxides	104	10	119	27
	225	100		
	275	200		
	300	300		
MoC-Mo_2C	121	10	60	28
	182	100		
WC-W_2C	101	10	90	28
	192	100		
NiZn-CoO	53	10	47	29
	160	100		
	200	200		
	220	300		
Ni_3N-VN	64	10	37	30
	218	100		
	380	200		
MoNi/$CoMoO_3$	18	10	35	31
	80	100		
	120	200		
NiMoCo	22	10	34	32
	120	100		
	175	200		
Ni-ZIF/NiB	67	10	101	33
	280	100		
Mo-Co_9S_8@C	113	10	68	34

	320	100		
Ni/Mo_2C	24<br>190	10<br>100	58	35
$PBA@Co(OH)_2$	140<br>260<br>290<br>320	10<br>100<br>200<br>400	100	36
$Ni_4Mo/MoO_x/Cu$	16<br>120	10<br>100	64	37
$Cr\text{-}Co_4N$	21<br>99<br>155<br>185	10<br>100<br>200<br>300	38	38
FeCoNi	64<br>180	10<br>100	125	39
NiFeSe	50<br>230<br>325	10<br>100<br>200	49	40
Pd,Ru-$MoS_{2-x}OH_y$	48<br>131	10<br>50	45	41
C-NiO	27<br>72	10<br>50	36	42
Sr_2RuO_4	61<br>140	10<br>50	51	43
$C\text{-}MoS_2$	45<br>115	10<br>50	46	44
$La_{0.5}Sr_{0.5}CoO_{3-\delta}\&MoSe_2$	250<br>265	10<br>50	34	45
$Ni/Ni(OH)_2$	77<br>125	10<br>50	53	46
W_2N/WC	149<br>250	10<br>50	47	47
RhCu	78	10	40	48

Supplementary Table 4. The HER performance of earth-abundant alloy electrocatalysts in alkaline electrolyte.

Electrocatalysts	Electrolyte	Overpotentials (mV)	Current density (mA cm^{-2})	Tafel slopes (mV/dec)	Refs.
Co_3Mo/Cu	1 M KOH	12	10	40	This work
		54	50		
		65	100		
		76	200		
		86	300		
		96	400		
	0.1 M KOH	62	30	50	
NiMo nanopower	1 M KOH	80	10	-	49
NiMo nanopower	2 M KOH	70	20	-	49
NiMo nanorod	1 M KOH	60	10	76	50
		200	100		
NiMo on stainless steel skeleton (40°C)	1 M KOH	70	10	-	51
		110	100		
NiMo on Ni (70°C)	30 wt% KOH	60	500	-	52
$Fe_{80}Mo_{20}$	1 M NaOH	200	10	115	53
NiZn	1 M NaOH	115	10	119	54
$MoNi_4$	1 M KOH	30	10	36	55
MoNi	1 M NaOH	290	10	132	56
		460	100		
$MoNi_2$	1 M NaOH	275	10	142	56
		410	100		
$MoNi_3$	1 M NaOH	380	10	148	56
		490	100		
Fe_2Ni	1 M KOH	87	10	35.2	57
		240	100		
		280	400		
CuTi	0.1 M KOH	87	30	110	58

Supplementary Table 5. Comparisons of the overall splitting water performance of NP Cu/Co_3Mo in alkaline electrolyte with representative electrocatalysts reported previously.

Electrocatalysts	Overpotentials (V)	Current density (mA cm^{-2})	Refs.
Co_3Mo/Cu||Co_3Mo/Cu	1.48	10	This work
	1.57	50	
	1.62	100	
	1.68	200	
	1.73	300	
	1.78	400	
NiFe/Cu||NiFe/Cu	1.53	10	This work
	1.72	50	
	1.77	100	
	1.82	200	
	1.89	400	
Cu_3N||Cu_3N	1.60	10	14
	1.75	50	
	1.80	100	
Ni/γ-Fe_2O_3||Ni/γ-Fe_2O_3	1.47	10	16
	1.70	50	
	1.77	100	
NiFe-LDH@NiCu||$NiFeO_x$@NiCu	1.53	10	25
	1.73	50	
	1.84	100	
Co_3S_4/MOF||Co_3S_4/MOF	1.55	10	26
	1.79	50	
	1.90	100	
CoFeZr oxides||CoFeZr oxides	1.63	10	27
	1.74	50	
	1.78	100	
	1.82	200	
	1.84	300	
Ni-ZIF/NiB||Ni-ZIF/NiB	1.54	10	33
	1.73	50	
	1.77	100	
PBA@$Co(OH)_2$||PBA@$Co(OH)_2$	1.65	10	36
	1.84	50	
	1.99	100	
FeCoNi nanosheets/CC|| FeCoNi nanosheets/CC	1.55	10	39
	1.86	50	

	2.00	100	
	2.13	200	
	2.35	400	
NiFeSe‖NiFeSe	1.56	10	40
	1.76	50	
	1.86	100	
Fe_2Ni‖Fe_2Ni	-	10	57
	1.67	50	
	1.73	100	
	1.81	200	
	1.92	400	
NiMoO‖NiMoO	1.54	10	59
	1.71	50	
	1.83	100	
LaBaSrCoOF‖LaBaSrCoOF	1.67	10	60
	1.76	50	
	1.82	100	
$NiCo_2O_4$‖$NiCo_2O_4$	1.61	10	61
	1.77	50	
	1.85	100	
FeCoNi nanotubes/Ni‖	1.43	10	62
FeCoNi nanotubes/Ni	1.60	50	
	1.73	100	
CoP/NC‖CoP/NC	1.64	10	63
	1.83	50	
	1.90	100	
	1.95	200	
$CoSn_2$‖$CoSn_2$	1.55	10	64
	1.65	50	
	1.69	100	
NiCoP@NC‖NiCoP@NC	1.58	10	65
	1.63	50	
	1.70	100	
	1.76	200	
$NiFe(OH)_x/FeS$‖$MoNi_4/MoO_2$	1.50	10	66
	1.60	50	
	1.68	100	
$NiCo_2S_4$‖$NiCo_2S_4$	1.58	10	67
	1.78	50	
	1.95	100	
CoMoNiS‖CoMoNiS	1.54	10	68
	1.86	50	
	2.09	100	
NiVIr-LDH‖NiVIr-LDH	1.50	10	69

	1.59	50	
	1.66	100	
	1.76	200	
Ir/MoS_2‖Ir/MoS_2	1.57	10	70
	1.71	50	
	1.78	100	
$Fe_3Co(PO_4)_4$@rGO‖Pt/C	1.45	10	71
	1.56	50	
	1.62	100	
Cr-doped FeNi-P‖Cr-doped FeNi-P	1.54	10	72
	1.65	50	
	1.70	100	
$LiCo(H_2O)_2[BP_2O_8]\cdot H_2O$‖ $LiCo(H_2O)_2[BP_2O_8]\cdot H_2O$	1.53	10	73
	1.67	50	
	1.72	100	
	1.77	200	
	1.80	400	
O-CoP‖O-CoP	1.60	10	74
	1.74	50	
	1.79	100	
V-CoP/CeO_2‖V-CoP/CeO_2	1.56	10	75
	1.67	50	
	1.71	100	
Ni-P-B‖Ni-P-B	-	10	76
	1.64	50	
	1.67	100	
	1.72	200	
	1.76	300	
NiMoN/CFC‖NiMoN/CFC	1.64	10	15
	1.84	50	
Ni_3N-VN‖Ni_3P-VP_2	1.51	10	30
	1.72	50	
Mo-Co_9S_8@C‖Mo-Co_9S_8@C	1.56	10	34
	1.95	50	
Ni/Mo_2C‖Ni/Mo_2C	1.64	10	35
	1.80	50	
Ni/$Ni(OH)_2$‖Ni/$Ni(OH)_2$	1.60	10	46
	1.68	50	
W_2N/WC‖W_2N/WC	1.58	10	47
	1.80	50	
RhCu‖RhCu	1.59	10	48
	1.88	50	

Supplementary Note 1

TOF values were calculated according to previous method.[2-8]

$$\mathrm{TOF} = N_{\mathrm{TH}}/N_{\mathrm{AS}} \qquad \text{Supplementary Equation 1}$$

where N_{TH} is the number of total hydrogen turnovers per geometric area, and N_{AS} is the number of active sites per geometric area. The values of N_{TH} and N_{AS} are calculated according to the equations:

$$N_{\mathrm{TH}} = (j\,\frac{\mathrm{mA}}{\mathrm{cm}^2})(\frac{1\ \mathrm{Cs}^{-1}}{1000\ \mathrm{mA}})(\frac{1\ \mathrm{mol\ e}^-}{96485.3\ \mathrm{C}})(\frac{1\ \mathrm{mol\ H_2}}{2\ \mathrm{mol\ e}^-})(\frac{6.022*10^{23}\ \mathrm{H_2\ molecules}}{1\ \mathrm{mol\ H_2}})$$

Supplementary Equation 2

$$N_{\mathrm{AS}} = (\frac{\mathrm{No.of\ atoms/unit\ cell}}{\mathrm{Volume/unit\ cell}})^{\frac{2}{3}}$$

Supplementary Equation 3

For the FCC Cu with $a = b = c = 3.615$ Å, the volume of unit cell containing 4 atoms is 47.232 Å^3. Therefore, the $N_{\mathrm{AS,Cu}} = 1.928\times10^{15}$ atoms cm^{-2}. On the basis of double-layer capacitance, the ECSA is calculated to be 11.4×10^3 according to

$$A_{\mathrm{ECSA}}\ (\mathrm{Cu}) = \mathrm{C_{dl}/C_s} \qquad \text{Supplementary Equation 4}$$

Here, C_s refers to specific capacitance of Cu, which is 28 μF cm^{-2}.[76]

As a result of the current density of 6.58 mA at the overpotential of 100 mV, TOF (Cu) = 0.000935 s^{-1}.

While for the intermetallic Co_3Mo, the volume of unit cell is 93.854 Å^3, and each unit cell contains 6 Co atoms and 2 Mo atoms. The value of $N_{\mathrm{AS,Co3Mo}}$ is determined to be 1.937×10^{15} atoms cm^{-2}.

To prevent the effect of Cu skeleton with small TOF value, we calculate the TOF of Co_3Mo at 100 mV overpotential through current density j and A_{ECSA} of the Co_3Mo/Cu electrode with/without H_2SO_4 treatment. Because the C_{dl} of Cu electrode with H_2SO_4 treatment decreases by 17 mF cm^{-2}, we generally assume the C_{dl} of Co_3Mo/Cu electrode with H_2SO_4 treatment reduces by 17 mF cm^{-2} owing to the Cu skeleton becoming smoother. The specific capacitance for a flat surface is in the range of 20-60 μF cm^{-2}, and we use an average of 40 μF cm^{-2} here.[2,6] The current densities of the

Co_3Mo/Cu electrode with and without H_2SO_4 treatment are 134.8 mA cm^{-2} and 423.4 mA cm^{-2}, whose A_{ECSA} are 283 mF cm^{-2} and 324 mF cm^{-2}.

$$\mathrm{TOF\ (Co_3Mo)} = \frac{(3.12*10^{15}\ \frac{\frac{\mathrm{H_2}}{\mathrm{s}}}{\mathrm{cm^2}}\ \mathrm{per}\frac{\mathrm{mA}}{\mathrm{cm^2}})*(423.4-134.8)\ \mathrm{mA\ cm^{-2}}}{1.937\ \times\ 10^{15}\mathrm{atoms\ cm^{-2}}\ *\frac{(324-17-283)\ \mathrm{mF\ cm^{-2}}}{40\ \mathrm{\mu F\ cm^{-2}}}} = 0.775\ \mathrm{s^{-1}}$$

Calculation of specific current density (*j*$_s$)

$$j_{\mathrm{s}} = j_{\mathrm{geo}}/A_{\mathrm{ECSA}} \qquad \text{Supplementary Equation 5}$$

At the overpotential of 100 mV, $j_{\mathrm{s,Cu}}$ = 0.578 μA cm^{-2}.

$$j_{\mathrm{Co3Mo/Cu}} = j_{\mathrm{s,Co3Mo}} \times A_{\mathrm{Co3Mo}} + j_{\mathrm{s,Cu}} \times A_{\mathrm{Cu}} \qquad \text{Supplementary Equation 6}$$

$$j_{\mathrm{Co3Mo/Cu\text{–}H}} = j_{\mathrm{s,Co3Mo}} \times A_{\mathrm{Co3Mo\text{–}H}} + j_{\mathrm{s,Cu}} \times A_{\mathrm{Cu\text{–}H}} \qquad \text{Supplementary Equation 7}$$

Here, A_{Co3Mo} and A_{Cu} refer to ECSA of Co_3Mo and Cu of Co_3Mo/Cu electrode, $A_{\mathrm{Co3Mo\text{–}H}}$ and $A_{\mathrm{Cu\text{–}H}}$ refers to ECSA of Co_3Mo and Cu of Co_3Mo/Cu electrode after H_2SO_4 treatment, respectively. Besides, $A_{\mathrm{Cu}} - A_{\mathrm{Cu\,\text{–}H}} \approx$ 17 mF cm^{-2}/28 μF cm^{-2}, $A_{\mathrm{Co3Mo}} - A_{\mathrm{Co3Mo\text{–}H}} \approx$ (324−283−17) mF cm^{-2}/40 μF cm^{-2}.

As a result, $j_{\mathrm{s,Co3Mo}}$ = 0.475 mA cm^{-2}

Supplementary references

1. JCPDS-ICCD, *Powder Diffraction File*, Joint Committee On Powder Diffraction Standards, International Center for Diffraction Data, Pennsylvania, USA (2001).
2. Zhang, R., Wang, X.X., Yu, S.J., Wen, T., Zhu, X.W., Yang, F.X., Sun, X.N., Wang, X.K. & Hu, W.P. Ternary $NiCo_2P_x$ nanowires as pH-universal electrocatalysts for highly efficient hydrogen evolution reaction. *Adv. Mater.* **29**, 1605502 (2017).
3. Xue, Y.R., Hui, L., Yu, H.D., Liu, Y.X., Fang, Y., Huang, B.L., Zhao, Y.J., Li, Z.B. & Li, Y.L. Rationally engineered active sites for efficient and durable hydrogen generation. *Nat. Commun.* **10**, 2281 (2019).
4. Ling, T., Yan, D.Y., Wang, H., Jiao, Y., Hu, Z.P., Zheng, Y., Zheng, L.R., Mao, J., Liu, H., Du, X.W., Jaroniec, M. & Qiao, S.Z. Activating cobalt(II) oxide nanorods for efficient electrocatalysis by strain engineering. *Nat. Commun*. **8**, 1509 (2017).
5. Yu, F., Zhou, H.Q., Huang, Y.F., Sun, J.Y., Qin, F., Bao, J.M., Goddardlll, W.A., Chen, S. & Ren, Z.F. High-performance bifunctional porous non-noble metal phosphide catalyst for overall water splitting. *Nat. Commun*. **9**, 2551 (2018).
6. Wu, Y.S., Liu, X.J., Han, D.D., Song, X.Y., Shi, L., Song, Y., Niu, S.W., Xie, Y.F., Cai, J.Y., Wu, S.Y., Kang, J., Zhou, J.B., Chen, Z.Y., Zheng, X.S., Xiao, X.H. & Wang, G.M. Electron density modulation of $NiCo_2S_4$ nanowires by nitrogen incorporation for highly efficient hydrogen evolution catalysis. *Nat. Commun*. **9**, 1425 (2018).
7. Hu, J., Zhang, C.X., Jiang, L., Lin, H., An, Y.M., Zhou, D., Leung, M.K.H. & Yang, S.H. Nanohybridization of MoS_2 with layered double hydroxides efficiently synergizes the hydrogen evolution in alkaline media. *Joule* **1**, 383-393 (2017).
8. Chen, Z.Y., Song, Y., Cai, J.Y., Zheng, X.S., Han, D.D., Wu, Y.S., Zang, Y.P., Niu, S.W., Liu, Y., Zhu, J.F., Liu, X.J. & Wang, G.M. Tailoring the d-band centers enables Co_4N nanosheets to be highly active for hydrogen evolution catalysis. *Angew. Chem. Int. Ed.* **57**, 5076-5080 (2018).
9. Waszczuk, P., Zelenay, P. & Sobkowski, J. Surface interaction of benzoic acid with a copper electrode. *Electrochim. Acta* **40**, 1717-1721 (1995).
10. Shen, Y., Zhou, Y.F., Wang, D., Wu. X., Li, J. & Xi, J.Y. Nickel-copper alloy encapsulated in graphitic carbon shells as electrocatalysts for hydrogen evolution reaction. *Adv. Energy Mater*. **8**, 1701759 (2018).
11. Feng, J.X., Wu, J.Q., Tong, Y.X. & Li, G.R. Efficient hydrogen evolution on Cu nanodots-decorated Ni_3S_2 nanotubes by optimizing atomic hydrogen adsorption and desorption. *J. Am. Chem. Soc.* **140**, 610-617 (2018).
12. Wang, Z.Y., Xu, L., Huang, F.Z., Qu, L.B., Li, J.T., Owusu, K.A., Liu, Z.A., Lin, Z.F., Xiang, B.H., Liu, X., Zhao, K.N., Liao, X.B., Yang, W., Cheng, Y.B. & Mai,

L.Q. Copper-nickel nitride nanosheets as efficient bifunctional catalysts for hydrazine-assisted electrolytic hydrogen production. *Adv. Energy Mater.* **9**, 1900390 (2019).

13. Hao, S.Y., Chen, L.C., Yu, C.L., Yang, B., Li, Z.J., Hou, Y., Lei, L.C. & Zhang, X.W. NiCoMo hydroxide nanosheet arrays synthesized via chloride corrosion for overall water splitting. *ACS Energy Lett.* **4**, 952-959 (2019).
14. Panda, C., Menezes, P.W., Zheng, M., Orthmann, S. & Driess, M. In situ formation of nanostructured core-shell Cu_3N-CuO to promote alkaline water electrolysis. *ACS Energy Lett*. **4**, 747-754 (2019).
15. Li, Y., Wei, X.F., Chen, L.S., Shi, J.L. & He, M.Y. Nickel-molybdenum nitride nanoplate electrocatalysts for concurrent electrolytic hydrogen and formate productions. *Nat. Commun*. **10**, 5335 (2019).
16. Suryanto, B.H.R., Wang, Y., Hocking, R.K., Adamson, W. & Zhao, C. Overall electrochemical splitting of water at the heterogeneous interface of nickel and iron oxide. *Nat. Commun.* **10**, 5599 (2019).
17. Jiang, K., Liu, B.Y., Luo, M., Ning, S.C., Peng, M., Zhao, Y., Lu, Y.R., Chan, T.S., Groot, F.M.F. & Tan, Y.W. Single platinum atoms embedded in nanoporous cobalt selenide as electrocatalyst for accelerating hydrogen evolution reaction. *Nat. Commun.* **10**, 1743 (2019).
18. Chen, J.Y., Ge, Y.C., Feng, Q.Y., Zhuang, P.Y., Chu, H., Cao, Y.D., Smith, W.R., Dong, P., Ye, M.X., Shen, J.F. Nesting Co_3Mo binary alloy nanoparticles onto molybdenum oxide nanosheet arrays for superior hydrogen evolution reaction. *ACS Appl. Mater. Interface* **11**, 9002-9010 (2019).
19. Luo, Y.T., Tang, L., Khan, U., Yu, Q.M., Cheng, H.M., Zou, X.L. & Liu, B.L. Morphology and surface chemistry engineering toward pH-universal catalysts for hydrogen evolution at high current density. *Nat. Commun.* **10**, 269 (2019).
20. Yu, L., Zhu, Q., Song, S.W., McElhenny, B., Wang, D.Z., Wu, C.Z., Qin, Z.J., Bao, J.M., Yu, Y., Chen, S. & Ren, Z.F. Non-noble metal-nitride based electrocatalysts for high-performance alkaline seawater electrolysis. *Nat. Commun.* **10**, 5106 (2019).
21. Zheng, Y.R., Wu, P., Gao, M.R., Zhang, X.L., Gao, F.Y., Ju, H.X., Wu, R., Gao, Q., You, R., Huang, W.X., Liu, S.J., Hu, S.W., Zhu, J.F., Li, Z.Y. & Yu, S.H. Doping-induced structural phase transition in cobalt diselenide enables enhanced hydrogen evolution catalysis. *Nat. Commun*. **9**, 2533 (2018).
22. Lu, X.F., Yu, L. & Lou, X.W. Highly crystalline Ni-doped FeP/carbon hollow nanorods as all-pH efficient and durable hydrogen evolving electrocatalysts. *Sci. Adv*. **5**, eaav6009 (2019).

23. He, Q., Tian, D., Jiang, H.L., Cao, D.F., Wei, S.Q., Liu, D.B., Song, P., Lin, Y. & Song, L. Achieving efficient alkaline hydrogen evolution reaction over a Ni_5P_4 catalyst incorporating single-atomic Ru sites. *Adv. Mater*. **32**, 1906972 (2020).
24. Zhang, J.Q., Shang, X., Ren, H., Chi, J.Q., Fu, H., Dong, B., Liu, C.G. & Chai, Y.M. Modulation of inverse spinel Fe_3O_4 by phosphorus doping as an industrially promising electrocatalyst for hydrogen evolution. *Adv. Mater*. **31**, 1905107 (2019).
25. Zhou, Y.F., Wang, Z.X., Pan, Z.Y., Liu, L., Xi, J.Y., Luo, X.L. & Shen, Y. Exceptional performance of hierarchical Ni-Fe (hydr) oxide@NiCu electrocatalysts for water splitting. *Adv. Mater*. **31**, 1806769 (2019).
26. Liu, T., Li, P., Yao, N., Kong, T.G., Cheng, G.Z., Chen, S.L. & Luo, W. Self-sacrificial template-directed vapor-phase growth of MOF assemblies and surface vulcanization for efficient water splitting. *Adv. Mater*. **31**, 1806672 (2019).
27. Huang, L.L., Chen, D.W., Luo, G., Lu, Y.R., Chen, C., Zou, Y.Q., Dong, C.L., Li, Y.F. & Wang, S.Y. Zirconium-regulation-induced bifunctionality in 3D cobalt-iron oxide nanosheets for overall water splitting. *Adv. Mater*. **31**, 1901439 (2019).
28. Lu, X.F., Yu, L., Zhang, J.T. & Lou, X.W. Ultrafine dual-phased carbide nanocrystals confined in porous nitrogen-doped carbon dodecahedrons for efficient hydrogen evolution reaction. *Adv. Mater*. **31**, 1900699 (2019).
29. Ling, T., Zhang, T., Ge, B.H., Han, L.L., Zheng, L.R., Lin, F., Xu, Z.R., Hu, W.B., Du, X.W., Davey, K. & Qiao, S.Z. Well-dispersed nickel- and Zinc- tailored electronic structure of a transition metal oxide for highly active alkaline hydrogen evolution reaction. *Adv. Mater*. **31**, 1807771 (2019).
30. Yan, H.J., Xie, Y., Wu, A.P., Cai, Z.C., Wang, L., Tian, C.G., Zhang, X.M. & Fu, H.G. Anion-modulated HER and OER activities of 3D Ni-V-based interstitial compound heterojunctions for high-efficiency and stable over water splitting. *Adv. Mater*. **31**, 1901174 (2019).
31. Wang, D.W., Han, C., Xing, Z.C., Li, Q. & Yang, X.R. Pt-like catalytic behavior of MoNi decorated $CoMoO_3$ cuboid arrays for hydrogen evolution reaction. *J. Mater. Chem. A* **6**, 15558 (2018).
32. Hu, K.L., Wu, M.X., Hinokuma, S., Ohto, T., Wakisaka, M., Fujita, J. & Ito, Y. Boosting electrochemical water splitting via ternary NiMoCo hybrid nanowire arrays. *J. Mater. Chem. A* **7**, 2156 (2019).
33. Xu, H.B., Fei, B., Cai, G.H., Ha, Y., Liu, J., Jia, H.X., Zhang, J.C., Liu, M. & Wu, R.B. Boronization-induced ultrathin 2D nanosheets with abundant crystalline-amorphous phase boundary supported on nickel foam toward efficient water splitting. *Adv. Energy Mater*. **10**, 1902714 (2020).

34. Wang, L.G., Duan, X.X., Liu, X.J., Gu, J., Si, R., Qiu, Y., Qiu, Y.M., Shi, D.E., Chen, F.H., Sun, X.M., Lin, J.H. & Sun, J.L. Atomically dispersed Mo supported on metallic Co_9S_8 nanoflakes as an advanced noble-metal-free bifunctional water splitting catalyst working in universal pH conditions. *Adv. Energy Mater*. **10**, 1903137 (2020).
35. Li, M.X., Zhu, Y., Wang, H.Y., Wang, C., Pinna, N. & Lu, X.F. Ni strongly coupled with Mo_2C encapsulated in nitrogen-doped carbon nanofibers as robust bifunctional catalyst for overall water splitting. *Adv. Energy Mater*. **9**, 1803185 (2019).
36. Wang, Y.Q., Ma, J.Z., Wang, J., Chen, S., Wang, H.S. & Zhang, J.T. Interfacial scaffolding preparation of hierarchical PBA-based derivative electrocatalysts for efficient water splitting. *Adv. Energy Mater*. **9**, 1802939 (2019).
37. An, Y.M., Long, X., Ma, M., Hu, J., Lin, H., Zhou, D., Xing, Z., Huang, B.L. & Yang, S.H. One-step controllable synthesis of catalytic $Ni_4Mo/MoO_x/Cu$ nanointerfaces for highly efficient water reduction. *Adv. Energy Mater*. **9**, 1901454 (2019).
38. Yao, N., Li, P., Zhou, Z.R., Zhao, Y.M., Cheng, G.Z., Chen, S.L. & Luo, W. Synergistially tuning water and hydrogen binding abilities over Co_4N by Cr doping for exceptional alkaline hydrogen evolution electrocatalysis. *Adv. Energy Mater*. **9**, 1902449 (2019).
39. Zhang, Q.R., Bedford, N.M., Pan, J., Lu, X.Y. & Amal, R. A fully reversible water electrolyzer cell made up from FeCoNi (oxy)hydroxide atomic layers. *Adv. Energy Mater*. **9**, 1901312 (2019).
40. Yilmaz, G., Tan, C.F., Lim, Y.F. & Ho, G.W. Pseudomorphic transformation of interpenetrated Prussian blue analogs into defective nickel iron selenides for enhanced electrochemical and photo-electrochemical water splitting. *Adv. Energy Mater*. **9**, 1802983 (2019).
41. Luo, Z.Y., Zhang, H., Yang, Y.Q., Wang, X., Li, Y., Jin, Z., Jiang, Z., Liu, C.P., Xing, W. & Ge, J.J. Reactant friendly hydrogen evolution interface based on di-anionic MoS_2 surface. *Nat. Commun*. **11**, 1116 (2020).
42. Kou, T.Y., Chen, M.P., Wu, F., Smart, T.J., Wang, S.W., Wu, Y.S., Zhang, Y., Li, S.T., Lall, S., Zhang, Z.H., Liu, Y.S., Guo, J.H., Wang, G.M., Ping, Y. & Li, Y. Carbon doping switching on the hydrogen adsorption activity of NiO for hydrogen evolution reaction. *Nat. Commun*. **11**, 590 (2020).
43. Zhu, Y.L., Tahini, H.A., Hu, Z.W., Dai, J., Chen, Y.B., Sun, H.N., Zhou, W., Liu, M.L., Smith, S.C., Wang, H.T. & Shao, Z.P. Unusual synergistic effect in layered ruddlesden-popper oxide enables ultrafast hydrogen evolution. *Nat. Commun*. **10**, 149 (2019).

44. Zang, Y.P., Niu, S.W., Wu, Y.S., Zheng, X.S., Cai, J.Y., Ye, J., Xie, Y.F., Liu, Y., Zhou, J.B., Zhu, J.F., Liu, X.J., Wang, G.M. & Qian, Y.T. Tuning orbital orientation endows molybdenum disulfide with exceptional alkaline hydrogen evolution capability. *Nat. Commun.* **10**, 1217(2019).
45. Oh, N.K., Kin, C.M., Lee, J., Kwon, O., Choi, Y., Jung, G.Y., Lim, H.Y., Kwak, S.K., Kim, G. & Park, H. In-situ local phase-transitioned $MoSe_2$ in $La_{0.5}Sr_{0.5}CoO_{3-\delta}$ heterostructure and stable overall water electrolysis over 1000 hours. *Nat. Commun.* **10**, 1723(2019).
46. Dai, L., Chen, Z.N., Li, L.X., Yin, P.Q., Liu, Z.Q. & Zhang, H. Ultrathin Ni(0)-embedded $Ni(OH)_2$ heterostructured nanosheets with enhanced electrochemical overall water splitting. *Adv. Mater.* **32**, 1906915(2020).
47. Diao, J.X., Qiu, Y., Liu, S.Q., Wang, W.T., Chen, K., Li, H.L., Yuan, W.Y., Qu, Y.T. & Guo, X.H. Interfacial engineering of W_2N/WC heterostructures derived from solid-state synthesis: a highly efficient trifunctional electrocatalyst for ORR, OER, and HER. *Adv. Mater.* **32**, 1905679(2020).
48. Cao, D., Xu, H.X. & Cheng, D.J. Construction of defect-rich RhCu nanotubes with hughly active Rh_3Cu_1 alloy phase for overall water splitting in all pH values. *Adv. Energy Mater.* **10**, 1903038(2020).
49. McKone, J.R., Sadtler, B.F., Werlang, C.A., Lewis, N.S. & Gray, H.B. Ni-Mo nanopowders for efficient electrochemical hydrogen evolution. *ACS Catal.* **3**, 166-169 (2013).
50. Tian, J.Q., Cheng, N.Y., Liu, Q., Sun, X.P., He, Y.Q. & Asiri, A.M. Self-supported NiMo hollow nanorod array: an efficient 3D bifunctional catalytic electrode for overall water splitting. *J. Mater. Chem. A.* **3**, 20056 (2015).
51. Xiao, L., Zhang, S., Pan, J., Yang, C.X., He, M.L., Zhuang, L. & Lu, J.T. First implementation of alkaline polymer electrolyte water electrolysis working only with pure water. *Energy Environ. Sci.* **5**, 7869 (2012).
52. Brown, D.E., Mahmood, M.N., Man, M.C.M. & Turner, A.K. Preparation and characterization of low overvoltage transition metal alloy electrocatalysts for hydrogen evolution in alkaline solution. *Electrochim. Acta* **29**, 1551 (1984).
53. Rosalbino, F., Macciò, D., Saccone, A., Angelini, E., & Delfino, S. Fe-Mo-R (R = rare earth metal) crystalline alloys as a cathode material for hydrogen evolution reaction in alkaline solution. *International Journal of Hydrogen Energy* **36**, 1965 (2011).
54. Zheng, Z., Li, N., Wang, C.Q., Li, D.Y., Meng, F.Y. & Zhu, Y.M. Effects of CeO_2 on the microstructure and hydrogen evolution property of Ni-Zn coatings. *Journal of Power Sources* **222**, 88 (2013).

55. Jin, Y.S., Yue, X., Shu, C., Huang, S.L. & Shen, P.K. Three-dimensional porous $MoNi_4$ networks constructed by nanosheets as bifunctional electrocatalysts for overall water splitting. *J. Mater. Chem. A.* **5**, 2508 (2017).
56. Jakšić, J.M., Vojnović, M.V. & Krstajić, N.V. Kinetic analysis of hydrogen evolution at Ni-Mo alloy electrodes. *Electrochimica Acta* **45**, 4151 (2000).
57. Raja, D.S., Chuah, X. F. & Lu, S.Y. In situ grown bimetallic MOF-based composite as highly efficient bifunctional electrocatalyst for overall water splitting with ultrastability at high current densities. *Adv. Energy Mater*. **8**, 1801065 (2018).
58. Lu, Q., Hutchings, G.S., Yu, W.T., Zhou, Y., Forest, R.V., Tao, R.Z., Rosen, J., Yonemoto, R.T., Cao, Z.Y., Zheng, H.M., Xiao, J.Q., Jiao, F. & Chen, J.G. Highly porous non-precious bimetallic electrocatalysts for efficient hydrogen evolution. *Nat. Commun.* **6**, 6567 (2015).
59. Yu, Z.Y., Lang, C.C., Gao, M.R., Chen, Y., Fu, Q.Q., Duan, Y. & Yu, S.H. Ni-Mo-O nanorod-derived composite catalysts for efficient alkaline water-to-hydrogen conversion via urea electrolysis. *Energy Environ. Sci*. **11**, 1890 (2018).
60. Hua, B., Li, M., Pang, W.Y., Tang, W.Q., Zhao, S.L., Jin, Z.H., Zeng, Y.M., Amirkhiz, B.S. & Luo, J.L. Activating *p*-blocking centers in perovskite for efficient water splitting. *Chem* **4**, 2902 (2018).
61. Peng, S.J., Gong, F., Li, L.L., Yu, D.S., Ji, D.X., Zhang, T.R., Hu, Z., Zhang, Z.Q., Chou, S.L., Du, Y.H. & Ramakrishna S. Necklace-like multishelled hollow spinel oxides with oxygen vacancies for efficient water electrolysis. *J. Am. Chem. Soc.* **140**, 13644-13653 (2018).
62. Li, H.Y., Chen, S.M., Zhang, Y., Zhang, Q.H., Jia, X.F., Zhang, Q., Gu, L., Sun, X.M., Song, L. & Wang, X. Systematic design of superaerophobic nanotube-array electrode comprised of transition-metal sulfides for overall water splitting. *Nat. Commun.* **9**, 2452 (2018).
63. Pan, Y., Sun, K.A., Liu, S.J., Cao, X., Wu, K.L., Cheong, W.C., Chen, Z., Wang, Y., Li, Y., Liu, Y., Wang, D.S., Peng, Q., Chen, C. & Li, Y.D. Core-shell ZIF-8@ZIF-67-derived CoP nanoparticle-embedded N-doped carbon nanotube hollow polyhedron for efficient overall water splitting. *J. Am. Chem. Soc.* **140**, 2610-2618 (2018).
64. Menezes, P.W., Panda, C., Garai, S., Walter, C., Guiet, A. & Driess, M. Structurally ordered intermetallic cobalt stannide nanocrystals for high-performace electrocatalytic overall water-splitting. *Angew. Chem. Int. Ed.* **57**, 15237-15242 (2018).
65. Cao, B., Cheng, Y., Hu, M.H., Jing, P., Ma, Z.X., Liu, B.C., Gao, R. & Zhang, J. Efficient and durable 3D self-supported nitrogen-doped carbon-coupled

nickel/cobalt phosphide electrodes: stoichiometric ratio regulated phase- and morphology- dependent overall water splitting performance. *Adv. Funct. Mater.* **29**, 1906316 (2019).

66. Niu, S., Jiang, W.J., Tang, T., Yuan, L.P., Luo, H. & Hu, J.S. Autogenous growth of hierarchical $NiFe(OH)_x/FeS$ nanosheet-on-microsheet arrays for synergistically enhanced high-output water oxidation. *Adv. Funct. Mater.* **29**, 1902180 (2019).
67. Kang, Z., Guo, H.J., Wu, J., Sun, X., Zhang, Z., Liao, Q.L., Zhang, S.C., Si, H.N., Wu, P.W., Wang, L. & Zhang, Y. Engineering an earth-abundant element-based bifunctional electrocatalyst for highly efficient and durable overall water splitting. *Adv. Funct. Mater.* **29**, 1807031 (2019).
68. Yang, Y., Yao, H.Q., Yu, Z.H., Islam, S.M., He, H.Y., Yuan, M.W., Yue, Y.H., Xu, K., Hao, W.C., Sun, G.B., Li, H.F., Ma, S.L., Zapol, P. & Kanatzidis, M.G. Hierarchical nanoassembly of $MoS_2/Co_9S_8/Ni_3S_2/Ni$ as a highly efficient electrocatalyst for overall water splitting in a wide pH range. *J. Am. Chem. Soc.* **141**, 10417-10430 (2019).
69. Li, S., Xi, C., Jin, Y.Z., Wu, D.Y., Wang, J.Q., Liu, T., Wang, H.B., Dong, C.K., Liu, H., Kulinich, S.A. & Du, X.W. Ir-O-V catalytic group in Ir-doped $NiV(OH)_2$ for overall water splitting. *ACS Energy Lett.* **4**, 1823-1829 (2019).
70. Wei, S.T., Cui, X.Q., Xu, Y.C., Shang, B., Zhang, Q.H., Gu, L., Fan, X.F., Zheng, L.R., Hou, C.M., Huang, H.H., Wen, S.S. & Zheng, W.T. Irodium-triggered phase transition of MoS_2 nanosheets boosts overall water splitting in alkaline media. *ACS Energy Lett.* **4**, 368-374 (2019).
71. Sultan, S., Ha, M., Kim, D.Y., Tiwari, J.N., Myung, C.W., Meena, A., Shin, T.J., Chae, K.H. & Kim, K.S. Superb water splitting activity of the electrocatalyst $Fe_3Co(PO_4)_4$ designed with computation aid. *Nat. Commun.* **10**, 5195 (2019).
72. Wu, Y.Q., Tao, X., Qing, Y., Xu, H., Yang, F., Luo, S., Tian, C.H., Liu, M. & Lu, X.H. Cr-doped FeNi-P nanoparticles encapsulated into N-doped carbon nanotube as a robust bifunctional catalyst for efficient overall water splitting. *Adv. Mater.* **31**, 1900178 (2019).
73. Menezes, P.W., Indra, A., Zaharieva, I., Walter, C., Loos, S., Hoffmann, S., Schlögl, R., Dau, H. & Driess, M. Helical cobalt borophosphates to master durable overall water-splitting. *Energy Environ. Sci*. **12**, 988 (2019).
74. Zhou, G.Y., Li, M., Li, Y.L., Dong, H., Sun, D.M., Liu, X.E., Xu, L., Tian, Z.Q. & Tang, Y.W. Regulating the electronic structure of CoP nanosheets by O incorporation for high-efficiency electrochemical overall water splitting. *Adv. Funct. Mater*. **30**, 1905252 (2020).
75. Yang, L., Liu, R.M. & Jiao, L.F. Electronic redistribution: construction and modulation of interface engineering on CoP for enhancing overall water splitting. *Adv. Funct. Mater*. 1909618 (2020).

76. Hao, W.J., Wu, R.B., Huang, H., Ou, X., Wang, L.C., Sun, D.L., Ma, X.H. & Guo, Y.H. Fabrication of practical catalytic electrodes using insulating and eco-friendly substrates for overall water splitting. *Energy Environ. Sci*. **13**, 102-110 (2020).
