# Supplementary material for: Spontaneously separated intermetallic Co3Mo from nanoporous copper as versatile electrocatalysts for highly efficient water splitting
Source: Nat Commun. 2020 Jun 10;11:2940. doi: 10.1038/s41467-020-16769-6 (PMC7287083; doi:10.1038/s41467-020-16769-6)
Supplement: Supplementary file 2 — Peer Review File [file 41467_2020_16769_MOESM2_ESM.docx]

Peer Review Information

**Journal:** Nature Communications

**Manuscript title:** Spontaneously separated intermetallic Co3Mo from nanoporous copper as versatile electrocatalysts for highly efficient water splitting

**Corresponding author name(s):** Prof Xingyou Lang

**Editorial notes:**

**none**

**Reviewer comments & decisions:**

**Reviewer comments, first version:**

Reviewers' comments:

Reviewer #1 (Remarks to the Author):

My review of this manuscript is based on the view that Nature Communications is looking for studies that are technically sound and exhibit exceptionally high novelty, scientific insight, and practical impact. Based on these criteria, I feel it is not a good fit for this journal.

In general, the work appears to be technically sound and reports an interesting set of results, particularly regarding very high geometric activity toward the HER for the authors' dealloyed NP Cu/Co3Mo catalyst. The chemical characterization of the catalyst is clear and complete, and the electrochemical experiments are similarly thorough - although the lack of statistical data makes it difficult to assess the reproducibility of the work. My expertise is not in DFT calculations, but the results showing a significant effect of hydroxide adsorption on HBE are intriguing.

Despite the merits noted above, the overall novelty of this work is limited. The dealloying method that was used here to synthesize the Cu/Co3Mo catalyst, while interesting, has been demonstrated in prior work. An example is the publication by Lu et al (DOI: 10.1038/ncomms7567), which appeared in Nature Communications and included strikingly similar results in terms of catalyst morphology but has not been cited in this work. Similarly, alkaline HER catalysts comprising alloys of Ni, Fe, and Co along with Mo are now well known to exhibit excellent HER activity. Hence, although it is quite interesting to see this dealloying method applied to multi-component HER catalysts, the results are unsurprising in light of prior work.

This work also does not offer much in terms of new and valuable scientific insights. The authors have neither commented nor speculated as to the generality of the dealloying technique for producing other types of catalysts. The manuscript appears to argue alternately that the Co and Mo components are stable in the metallic state under HER conditions and also that hydroxlyated surfaces are responsible for the catalysis. These two ideas are at odds and critically important to understanding the composition/function of the catalyst. The DFT results are interesting in terms of the apparent effect of hydroxylation, but they have not been discussed in adequate detail, nor has any effort been made to connect this work to the now extensive recent work on understanding the influence of surface hydroxylation on Pt-based hydrogen electrocatalysis in base (see DOI:10.1126/science.1211934 and works citing it). Thus, it is hard to see how this study gainfully extends the boundary of what the research community already knows--which is that high surface-area M-Mo (where M is a Fe-group metal) catalysts are very active toward the HER.

The practical impact of this work is perhaps the strongest point, as the overall performance of the dealloyed catalyst toward the HER in particular is quite high. The OER performance is less remarkable, as NiFe oxides have been known for several years to exhibit comparable or lower onset overpotentials (see DOI: 10.1007/s12274-014-0591-z for a compact review up to 2015). In this context, the use of a single catalyst for HER and OER makes for an interesting narrative, but does not really contribute a practical advantage. The HER stability data also look promising, but I would have liked to see those experiments carried out for at least an order of magnitude longer time period, as practical electrolyzers need to operate continuously for thousands of hours. Another key practical weakness is that the authors have not attempted to quantify the number of active sites or even the total active mass of the catalyst, which makes it very difficult to compare the "intrinsic" activity of this material on an even basis with other catalysts. This is especially important given the unusual thickness (400 microns) of the nanoporous layer in this material, which is at least 10-fold greater than what is commonly used for thin-film catalysts on rotating-disk electrodes. This may partly account for the observed high activity--there is simply a very large loading of active catalyst per square cm.

To conclude, I see this study as technically sound and potentially of interest to the water electrolysis research community, but I cannot recommend acceptance for publication in Nature Communications.


Reviewer #2 (Remarks to the Author):

In their manuscript "Spontaneously separated intermetallic Co3Mo from nanoporous copper as versatile electrocatalysts for highly efficient water splitting" Q. Jiang et al. report Co/Mo/Cu electrocatalysts derived from alloys by leaching.
Introduction: For industrial application, nickel and/or stainless steel is used as electrodes – must be corrected.
4/64: “low-dimensional” is not correct since particles are still 3-D
4/75: “HBE” is used without introducing the abbreviation
5/98: “quasi-eutectic structure” not shown, subsequent argumentation hard to follow
6/112: the residual Al could be in the Cu-skeleton or the Co3Mo – in the latter, it would have a string impact on the discussion on the active phase. Any proof that the Al is in Cu?
6/113: any proof for the elemental Co? must be a quite large amount!
6/116 and Fig. 1c: what is visible are large Mo-containing areas where there is much less Co present than expected for Co3Mo – explanation?
7/131: ref. 35 is for sure not the first one reporting Co3Mo – correct reference needs to be used!
7/137 and Fig. 2: For the fitting of some of the XPS data different FWHM was used for the components of the same signal – is physically not correct!
7/140: Unusual to observe Mo in state 0, +4 and +6 simultaneously – has partial charging of the samples been taken into account? If the materials are oxidised by transfer to the XPS champer, it is very unlikely that they survive contact to the aqueous electrolyte …
8/146: DFT calculations are based on PBE, which is known to produce large errors in adsorption energies – not state of the art! How was the potential taken into consideration?
10/196: H2SO4 treatment would result in a smoother surface (since kinks and edges are dissolved quicker), thus less surface area which could also explain the loss of activity.
10/205: Durability test is not sufficient, since this would result in “stability” until the electrode is fully dissolved – ICP/MS measurement of electrolyte needed!
11/221: the “blue shift” seems to be a misalignment of the Raman spectrometer since also the CuO signals are “blue shifted”.
SI Fig. S2: Add insets from 25-50° 2-Theta to allow for phase assignment and add original references for structural data in caption

One major issue in the manuscript is that the electrolyte was not analysed by ICP/OES or better by ICP/MS to determine the concentration of metal ions after the catalysis. This is crucial to know if the materials are stable.

Another major issue concerns the material´s characterisation. It is not clear, where the residual Al is located and what the impact on the catalytic properties are. In addition, there seems to be Mo-rich areas after leaching and some of the experimental data is so light grey in the figures that it is hard to compare to the fits (Fig. 1, Fig. 2, Fig. S7, Fig. S12). Also, many claims by the authors lack experimental proof (e.g. 11/218: Mo-doped Co3O4 – no proof for doping given)

Concerning the statement of the authors, that their material is superior to known noble metal-free systems in HER, the following systems outperform theirs:
- MoNi/CoMoO3/Nickelfoam (18/10/35 – values as in Tables S1/S2), J. Mater. Chem. A, 2018, 6, 15558.
- NiCoMo-Nanowires/Nickelfoam (22/10/34), J. Mater. Chem. A, 2019, 7, 2156.

One minor issue: Naming of the materials has to be adopted to the common nomenclature, i.e. Co3Mo/Cu, since the Co3Mo particles are supported on the Cu and not vice versa.

Based on the above, I can not recommend to publish the manuscript.

**Author rebuttal, first version:**

***Reviewer #1 (Remarks to the Author):***

*My review of this manuscript is based on the view that Nature Communications is looking for studies that are technically sound and exhibit exceptionally high novelty, scientific insight, and practical impact. Based on these criteria, I feel it is not a good fit for this journal. In general, the work appears to be technically sound and reports an interesting set of results, particularly regarding very high geometric activity toward the HER for the authors' dealloyed NP Cu/Co3Mo catalyst. The chemical characterization of the catalyst is clear and complete, and the electrochemical experiments are similarly thorough - although the lack of statistical data makes it difficult to assess the reproducibility of the work. My expertise is not in DFT calculations, but the results showing a significant effect of hydroxide adsorption on HBE are intriguing.*

**Reply:** We thank the reviewer for finding interest of our work. We also appreciate the reviewer for the encouraging comments and constructive suggestions. Following these insightful comments and suggestions, we have carried out supplementary experiments, including STEM-EDS mapping of Co3Mo/Cu, preparation and electrochemical characterization of ten nanoporous Co3Mo/Cu electrodes, durability measurement of nanoporous Co3Mo/Cu electrode for 1000 h, evaluation of intrinsic activity of nanoporous Co3Mo/Cu electrode, as well as preparation and electrochemical characterization of nanoporous NiFe/Cu electrode for comparison. Based on these new results that demonstrate the outstanding performance of nanoporous Co3Mo/Cu, with exceptional stability, reproducibility and high intrinsic activity, we have completely revised the manuscript. The detailed corrections are listed below.

Developing low-cost and robust electrocatalysts is crucial for large-scale generation of hydrogen via electrochemical water splitting. Ideal electrode materials for highly efficient water electrolysis should comprise sufficient accessible surface with high intrinsic catalytic activity to lower their working overpotentials at high current densities, and have a rational and steady electrode structure to facilitate electron transfer/mass transport and withstand violent gas evolution. In addition, the electrode materials should be developed by a facile and scalable method. To satisfy these industrial requirements, in this work we have developed monolithic nonprecious electrode materials that are composed of intermetallic Co3Mo nanoparticles seamlessly integrated with hierarchical nanoporous Cu skeleton (Co3Mo/Cu) by a facile and scalable dealloying technology associated with a spontaneous phase separation. This method can be extended to develop various monolithic alloy/metal

1

hybrid electrodes with a nanoporous architecture by making use of their poor miscibility. In this work we demonstrate its feasibility and generality by taking nanoporous Co3Mo/Cu electrodes as model systems. In such unique monolithic electrodes, the constituent Co3Mo nanoparticles serve as electroactive sites with intrinsic catalytic activity of as high as ~0.48 mA cm^−2^ECSA and the interconnective Cu ligaments work as conductive materials to facilitate electron transfer. While for the hierarchical nanoporous architecture consisting of large channels and small nanopores, the former is conducive to mass transport and the latter enables more accessibility of electroactive Co3Mo nanoparticles. These features enlist nanoporous Co3Mo/Cu electrodes to exhibit a superior HER catalytic behavior in 1 M KOH solution, with ultralow onset overpotential and Tafel slope (~3 mV and ~44 mV dec−^1^). They reach a current density of 400 mA cm^−2^ at overpotentials of as low as 96 mV. Owing to sufficient steadiness of the Cu skeleton and the seamless Co3Mo/Cu interface, the nanoporous Co3Mo/Cu electrodes can endure the violent hydrogen evolution and maintain the initial electrode structure and catalysis in a long-term durability measurement for 1000 h. As one of the best nonprecious catalytic materials, it is distinguished from most nonprecious nanocatalysts of transition-metal-based compounds and alloys, which inevitably encounter supplementary and unstable interfaces linked to conductivity and durability issues because they usually have to be immobilized on a conductive substrate using insulative polymer binders. In addition, The structurally ordered intermetallic compounds enables a precise understanding of HER electrocatalysis of nonprecious alloy catalysts.

Regarding the reproducibility, we have prepared ten electrodes of nanoporous Co3Mo/Cu and characterized their HER catalytic properties. As shown in **Figure 3c** and supplementary **Figure 13a**, these ten Co3Mo/Cu electrodes exhibit quite high reproducibility, with almost the same HER properties.

After revision with these constructive comments and suggestions, we feel the paper becomes much more solid and stronger, as well as easier for readers to understand the novelty and significance of this work. In view of the novelty, excellent performances and reproducibility and scientific implications of the nanoporous intermetallic compound/metal electrodes for the HER and their electro-oxidized derivatives for the OER, we wish the reviewer could share our confidence and belief that the work reported in this paper deserves to be published in a high-impact journal, like *Nature Communications*.

*(1). Despite the merits noted above, the overall novelty of this work is limited. The dealloying method that was used here to synthesize the Cu/Co3Mo catalyst, while interesting, has been demonstrated in prior work. An example is the publication by Lu*

*2*

*et al (DOI: 10.1038/ncomms7567), which appeared in Nature Communications and included strikingly similar results in terms of catalyst morphology but has not been cited in this work. Similarly, alkaline HER catalysts comprising alloys of Ni, Fe, and Co along with Mo are now well known to exhibit excellent HER activity. Hence, although it is quite interesting to see this dealloying method applied to multi-component HER catalysts, the results are unsurprising in light of prior work.*

**Reply:** We agree with the reviewer that the dealloying method has been used to synthesize nanoporous electrocatalysts, including bimetallic Cu-Ti catalysts by Lu *et al* (DOI: 10.1038/ncomms7567), which has been added in the reference list. We also agree with the reviewer that alloys of Ni, Fe, and Co along with Mo exhibit excellent HER activity in alkaline. However, despite the extensive studies have conducted, the superior electrocatalytic activity of Ni-, Fe, Co-Mo alloys has not been fully exploited to meet the practical requirements in water electrolysis, i.e., high current densities at low overpotentials. Distinguished from those disordered alloy catalysts, we would like to address that structurally ordered intermetallic compounds are not only of scientific significance to demonstrate the electrocatalysis mechanism for designing novel and highly efficient HER catalysts but also of ultrahigh stability for practical applications for large-scale generation of hydrogen. Furthermore, the intermetallic Co3Mo nanoparticles that are seamless integrated with hierarchical nanoporous Cu skeleton exhibit exceptionally high intrinsic activity of 0.48 mA cm^−2^ECSA. This enables an outstanding HER performance of nanoporous Co3Mo/Cu, outperforming some of the best nonprecious catalysts based on Ni-Mo and Fe-Mo alloys and various compounds (supplementary **Table 4**). Although Cu-Ti bimetallic electrocatalyst has the similar nanoporous architecture, it has to take overpotential of 87 mV to reach current density of 30 mA cm−^2^ in 0.1 M KOH electrolyte because of the Tafel slope of as high as 110 mV dec^−1^, in sharp contrast with the corresponding values of nanoporous Co3Mo/Cu (62 mV and 50 mV dec^−1^) when measured at the same experimental conditions (supplementary **Table 4**). Despite the fact that the HER in alkaline media is much more sluggish than in acidic media, the nanoporous Co3Mo/Cu electrodes can reach the current density of 400 mA cm^−2^ at overpotential of as low as 96 mV in 1 M KOH electrolyte, comparable to commercially Pt/C catalyst in 1 M H2SO4 electrolyte. Besides, in this work we also propose a facile and novel strategy to develop monolithic alloy/metal hybrid electrodes with a nanoporous architecture, i.e., chemically dealloying associated with a spontaneous phase separation by making use of the poor miscibility of electroactive alloys and conductive metal substrates. This scalable method can be extended to develop various monolithic nanoporous alloy/metal hybrid electrocatalyst materials with high conductivity and steadiness.

3

*(2). This work also does not offer much in terms of new and valuable scientific insights. The authors have neither commented nor speculated as to the generality of the dealloying technique for producing other types of catalysts.*

**Reply:** We thank the reviewer for the constructive comments. Following the comment, we have highlighted the generality of our facile strategy of chemical dealloying associated with spontaneous phase separation to develop various monolithic alloy/metal hybrid electrode materials in the manuscript. At the same time, using our strategy we have demonstrated the feasibility and generality and developed similar electrode materials from precursor Cu8M3Mo1Al88 (M = Fe, Co and Ni) alloys. In all of these electrode materials, nanoporous Co3Mo/Cu electrodes exhibit the best electrocatalytic activities.

*(3) The manuscript appears to argue alternately that the Co and Mo components are stable in the metallic state under HER conditions and also that hydroxlyated surfaces are responsible for the catalysis. These two ideas are at odds and critically important to understanding the composition/function of the catalyst. The DFT results are interesting in terms of the apparent effect of hydroxylation, but they have not been discussed in adequate detail, nor has any effort been made to connect this work to the now extensive recent work on understanding the influence of surface hydroxylation on Pt-based hydrogen electrocatalysis in base (see DOI:10.1126/science.1211934 and works citing it). Thus, it is hard to see how this study gainfully extends the boundary of what the research community already knows--which is that high surface-area M-Mo (where M is a Fe-group metal) catalysts are very active toward the HER.*

**Reply:** We thank the reviewer for the insightful comment. Following the comment, we have corrected the presentation on the hydroxygenation of intermetallic Co3Mo surface. The internal Co and Mo atoms in the intermetallic Co3Mo nanoparticles present in metallic states and the surface Co and Mo atoms are in oxidized states of Co^2+^, Mo^4+^ and Mo^6+^, respectively. In the HER potential region, these oxidized Co and Mo atoms are electro-reduced to form partially hydroxygenated surfaces, which are responsible for the catalysis.

Considering that the practical catalyst surface is always in co-adsorption with HO^−^ ions in water electrolysis, we have re-performed DFT simulations based on the effect of the aqueous solvent environment, in which the electrolyte at the solid-liquid interface is treated as a polarizable continuum by placing point counter-charges via the linearized Poisson-Boltzmann equation using VASPsol code. Different from the model system of Pt-M(OH) (M = Fe, Co, Ni) hybrid for the hydrogen electrocatalysis in base, both Co and Mo components in our intermetallic Co3Mo are chemically

4

active and their surface atoms always present in oxidized states. In our DFT simulations, we have demonstrated that these oxidized surface atoms will be electro-reduced to generate the electroactive sites via the formation of partially hydroxygenated Co3Mo surface. As shown in supplementary **Figure 12**, the coverage of hydroxyl groups on the Co3Mo(002) surface decreases as the potential decreases from 0.6 to -0.6 V versus RHE. At the potential of 0 V versus RHE, DFT simulations demonstrate that the hydroxyl groups tend to be adsorbed on the Co-Co-Mo hollow sites with a coverage of 1/2 (supplementary **Figure 12b**). Owing to the adsorption of hydroxyl groups, it remarkably weakening the HBE (-0.36 eV) of their adjacent Co-Co-Mo hollow sites in comparison with the value of bare Co3Mo(002) surface (-0.77eV). These results demonstrate that it is important to consider the effect of hydroxyl coverage on nonprecious metal or alloy surface on the catalysis.

Owing to the proper HBE value of the hydroxygenated Co3Mo, the nanoporous Co3Mo/Cu electrode exhibits the best HER catalysis in alkaline media. As shown in below figure, the overpotential of nanoporous Co3Mo/Cu is ~72 mV lower than those of nanoporous catalysts that are prepared from Cu8M3Mo1Al88 (M = Fe and Ni) at current density of -400 mA cm^-2^. This is different from Pt-M(OH) hybrid electrocatalysts, in which the Pt-Ni(OH)2 is usually expected to be the best one.

*(4). The practical impact of this work is perhaps the strongest point, as the overall performance of the dealloyed catalyst toward the HER in particular is quite high. The OER performance is less remarkable, as NiFe oxides have been known for several years to exhibit comparable or lower onset overpotentials (see DOI: 10.1007/s12274-014-0591-z for a compact review up to 2015). In this context, the use*

5

*of a single catalyst for HER and OER makes for an interesting narrative, but does not really contribute a practical advantage.*

**Reply:** We appreciate the reviewer for approving our electrocatalysts with high overall HER performance for practical applications. We also agree with the reviewer that the NiFe oxide as one of the best OER catalysts usually exhibit lower overpotential. Following this comment, we have prepared nanoporous NiFe/Cu electrode and its electro-oxidized derivative (EO NiFe/Cu) by the same procedure (supplementary **Figure 20a,c**). Whereas the EO NiFe/Cu exhibits slightly higher OER activity than the EO Co3Mo/Cu, the HER activity of nanoporous NiFe/Cu electrode is much lower than that of nanoporous Co3Mo/Cu electrode (supplementary **Figure 20b,d**). This enables inferior electrochemical performance of alkaline electrolyzer based on nanoporous NiFe/Cu, which has to take the overpotential of ~530 mV to deliver the current density of 100 mA cm−^2^ for the water electrolysis. This is much higher than the one based on nanoporous Co3Mo/Cu (~390 mV) (**Figure 5b**). The sharp contrast demonstrates the practical advantage of nanoporous Co3Mo/Cu and EO Co3Mo/Cu as cathode and anode for highly efficient water electrolysis.

(5). *The HER stability data also look promising, but I would have liked to see those experiments carried out for at least an order of magnitude longer time period, as practical electrolyzers need to operate continuously for thousands of hours.*

**Reply:** We appreciate the reviewer for the constructive comment and suggestion. Following this suggestion, we have performed long-term durability measurements of nanoporous Co3Mo/Cu electrodes in 1 M KOH at overpotentials of 60 and 80 mV for more than 1000 and 500 hours, respectively. As shown in **Figure 3g**, they keep stable current densities during the stability measurements. Furthermore, after 1000 h, the nanoporous Co3Mo/Cu electrode displays the same microstructure and exhibits almost the same HER polarization curve as the initial one (inset of **Figure 3g**).

(6). *Another key practical weakness is that the authors have not attempted to quantify the number of active sites or even the total active mass of the catalyst, which makes it very difficult to compare the "intrinsic" activity of this material on an even basis with other catalysts. This is especially important given the unusual thickness (400 microns) of the nanoporous layer in this material, which is at least 10-fold greater than what is commonly used for thin-film catalysts on rotating-disk electrodes. This may partly account for the observed high activity--there is simply a very large loading of active catalyst per square cm.*

**Reply:** Following this constructive suggestion, we have evaluated the intrinsic activity of Co3Mo in view that it is expected to electroactive sites of nanoporous

6

Co3Mo/Cu electrode. With an assumption that the dramatic reduction in geometric current density results from the electrochemical surface area (ECSA) change due to the loss of both Co3Mo nanoparticles and Cu kinks or edges on ligament surface (supplementary **Figure 17**), the intrinsic activity of Co3Mo is evaluated to be 0.48 mA cm^−2^ECSA, ~830-fold higher than that of bare Cu (0.58 A cm^−2^ECSA) (supplementary **Figure 18a**). This is confirmed by the turnover frequency (TOF), the number of H2 molecule evolved per second per active site. As illustrated in supplementary **Figure 18b**, the TOF of Co3Mo is calculated to be 0.775 s^−1^ at the overpotential of 100 mV in 1 M KOH, much higher than some of the best nonprecious HER catalysts reported previously (supplementary **Table 1**).

*To conclude, I see this study as technically sound and potentially of interest to the water electrolysis research community, but I cannot recommend acceptance for publication in Nature Communications.*

**Reply:** Finally, we would like to appreciate the reviewer again for his/her insightful comments and constructive suggestions, according to which we have completely revised our manuscript. We feel the revision becomes much more solid and stronger, as well as easier for readers to understand the novelty and significance of this work. In view of the novelty in both preparation method of monolithic electrode materials and electroactive intermetallic compounds, the excellent performances and reproducibility, as well as the scientific implications of intermetallic compounds for the HER and their electro-oxidized derivatives for the OER, we wish the reviewer could share our confidence and belief that the work reported in this paper deserves to be published in a high-impact journal, like *Nature Communications*.

7

***Reviewer #2 (Remarks to the Author):***

*In their manuscript "Spontaneously separated intermetallic Co3Mo from nanoporous copper as versatile electrocatalysts for highly efficient water splitting" Q. Jiang et al. report Co/Mo/Cu electrocatalysts derived from alloys by leaching.*

**Reply:** We appreciate the reviewer for his/her constructive comments and suggestions. Following these comments and suggestion, we have carried out supplementary experiments, including TEM and SEM-EDS mapping characterizations of the Cu8Co3Mo1Al88 alloy precursor, SEM-EDS mapping and STEM-EDS mapping characterization of nanoporous Co3Mo/Cu, 1000-hour long-term durability measurement of nanoporous Co3Mo/Cu and ICP-OES analysis of electrolyte. At the same time, we have re-performed DFT simulations based on strongly constrained appropriately normed (SCAN). According to these supplementary results and the reviewer′s constructive suggestions, we have completely revised our manuscript. The detailed corrections and modifications are listed below.

(1). *Introduction: For industrial application, nickel and/or stainless steel is used as electrodes – must be corrected.*

**Reply:** According to this suggestion, we have corrected and mentioned that nickel and/or stainless steel as electrodes are used for industrial application in Introduction section.

(2). *4/64: “low-dimensional” is not correct since particles are still 3-D.* **Reply:** According to this comment, we have corrected the presentation.
(3). *4/75: “HBE” is used without introducing the abbreviation*

**Reply:** We have defined the abbreviation of "HBE" to be "hydrogen binding energy".

(4). *5/98: “quasi-eutectic structure” not shown, subsequent argumentation hard to follow.*

**Reply:** We appreciate the reviewer for the constructive comment. Following this comment, we have carried out supplementary microstructure characterizations on the precursor alloy of Cu8Co3Mo1Al88. The quasi-eutectic structure is shown in supplementary **Figure 3**.

(5). *6/112: the residual Al could be in the Cu-skeleton or the Co3Mo – in the latter, it would have a string impact on the discussion on the active phase. Any proof that the Al is in Cu?*

8

**Reply:** We thank the reviewer for the constructive comment, according to which we have performed supplementary STEM-EDS elemental mapping characterization and low-energy ion scattering (LEIS) spectrum measurement. The detailed results are shown in **Figure 1c** and supplementary **Figure 6**, wherein the residual Al is mainly in the interior of Cu skeleton, not in the Co3Mo nanoparticles.

(6). *6/113: any proof for the elemental Co? must be a quite large amount!*

**Reply:** Following this suggestion, we have carried out additional SEM-EDS mapping and EDS spectrum characterization on the as-prepared nanoporous Co3Mo/Cu electrode. The detailed results are shown in supplementary **Figure 5**, where the Co/Mo atomic ratio is ~25/3, in agreement with XPS and ICP-OES measurements.

(7). *6/116 and Fig. 1c: what is visible are large Mo-containing areas where there is much less Co present than expected for Co3Mo – explanation?*

**Reply:** We thank the reviewer for the constructive comment. According to this comment, we have re-performed supplementary STEM-EDS mapping characterization to demonstrate the distribution of Cu, Co and Mo in the nanoporous Co3Mo/Cu electrode. The detailed results are shown in **Figure 1c**. Therein, the nanoparticles grown on the protuberant Cu ligament are composed of both Co and Mo. The former is more than the latter, which implies the formation of Co3Mo nanoparticles. This is consistent with HAADF-STEM image of Co3Mo nanoparticles that are grown on Cu substrate (**Figure 1d**).

(8). *7/131: ref. 35 is for sure not the first one reporting Co3Mo – correct reference needs to be used!*

**Reply:** According to this suggestion, we have added a reference, in which intermetallic Co3Mo was firstly confirmed in Co-Mo phase diagram.

(9). *7/137 and Fig. 2: For the fitting of some of the XPS data different FWHM was used for the components of the same signal – is physically not correct!*

**Reply:** We appreciate the reviewer for the comment, according to which we have corrected the XPS fitting for the Mo^4+^ and Mo^6+^ signal with the same FWHM. While for the Mo^0^ signal, the 3d3/2 and 3d5/2 peaks are fitted with the FWHM ratio of 1.49/1 in term of the instructions. In addition, we also have corrected the Co^2+^ and Co^0^ fitting by controlling their FWHM. The detailed results are shown in **Figure 2a**.

(0). *7/140: Unusual to observe Mo in state 0, +4 and +6 simultaneously – has* 9

*partial charging of the samples been taken into account? If the materials are oxidised by transfer to the XPS champer, it is very unlikely that they survive contact to the aqueous electrolyte ...*

**Reply:** We thank the reviewer for the comment. To address this concern, we have double-checked the Mo 3d XPS spectrum and re-performed XPS characterization of the as-prepared nanoporous Co3Mo/Cu electrode. We make sure that the Mo component indeed presents in the chemical states of Mo^0^, Mo^4+^ and Mo^6+^. The Mo^0^ corresponds to the internal Mo atoms in the intermetallic Co3Mo nanoparticles. While for the Mo^4+^ and Mo^6+^, they are due to the surface oxidation of Mo atoms during the rinsing and desiccating treatment at room temperature for the XPS measurement of nanoporous Co3Mo/Cu electrode. Nevertheless, the oxidized surface Mo atoms will be electro-reduced to form partially hydroxygenated Co3Mo surface in the HER potential region at pH =14. This is verified by initial LSV polarization curves of nanoporous Co3Mo/Cu electrode in 1 M KOH (supplementary **Figure 11**). Such processes are also demonstrated by first-principles simulation based on the meta-generalized gradient approximation (meta-GGA) with strongly constrained appropriately normed (SCAN) functional. As shown in supplementary **Figure 12**, it is thermodynamically stable for the Co3Mo(002) surface with 1/2 monolayer *OH. With such atomic configuration, the free energy for atomic hydrogen adsorption (Δ*G*H*) becomes almost thermoneutral, suggesting that the hydroxygenated Co3Mo is more favorable for hydrogen adsorption/desorption (**Figure 2b**).

*(11). 8/146: DFT calculations are based on PBE, which is known to produce large errors in adsorption energies – not state of the art! How was the potential taken into consideration?*

**Reply:** We appreciate the reviewer for the constructive comment. Following this comment, we have re-carried out DFT simulations based on the meta-generalized gradient approximation (meta-GGA) with strongly constrained appropriately normed (SCAN) functional in view that in many successful cases, the geometries and energies of various materials and molecules can be more accurately predicted using SCAN functional than PBE. Furthermore, implicit solvation model was employed to perform all DFT calculations considering that the solid-liquid interfaces are the heart of electrocatalytic processes. The detailed results are shown in **Figure 2b,c** and supplementary **Figure 12**. As shown in the free energy diagrams of atomic hydrogen adsorption (**Figure 2b**) and hydroxide adsorption (supplementary **Figure 12**), the results based on SCAN exhibit similar trend observed in the ones based on PBE. It should be noted that using SCAN, the free energy for atomic hydrogen adsorption

10

(Δ*G*H*) on hydroxygenated Co3Mo(002) is -0.142 eV, in contrast with bare Co3Mo(002) (-0.549 eV).

(12). *10/196: H2SO4 treatment would result in a smoother surface (since kinks and edges are dissolved quicker), thus less surface area which could also explain the loss of activity.*

**Reply:** We appreciate the reviewer for the insightful comment. To demonstrate the change of surface area, we have carried out supplementary electrochemical double-layer capacitance (*C*dl) measurements of nanoporous bare Cu and nanoporous Co3Mo/Cu after the H2SO4 treatment. As shown in supplementary **Figure 17,** the *C*dl values of the H2SO4-treated nanoporous Cu and Co3Mo/Cu electrodes indeed decreases relative to their initial values. For the nanoporous bare Cu, there is only ~5.3% *C*dl change because of a smoother surface due to the dissolution of kinks and edges. While for the nanoporous Co3Mo/Cu electrode, the *C*dl,Co3Mo/Cu value decreases to 283 from 324 mF cm^-2^, with 12.6% of ECSA change, due to the loss of both Co3Mo nanoparticles and surface kinks and edges of Cu ligaments. In view of the ultralow intrinsic catalytic activity of bare Cu (0.58 A cm-2ECSA), the remarkable reduction in the current density of nanoporous Co3Mo/Cu should attributed to the loss of Co3Mo nanoparticles.

(13). *10/205: Durability test is not sufficient, since this would result in “stability” until the electrode is fully dissolved – ICP/MS measurement of electrolyte needed!*

**Reply:** We appreciate the reviewer for the suggestion, according to which we have analyzed the electrolyte after a long-term durability measurement, using ICP-OES. The detailed results are shown in supplementary **Table 2**. There are no Co and Mo ions to be detected by ICP-OES in the tested electrolyte except for ignorable Cu and Al. This implies the exceptional stability of nanoporous Co3Mo/Cu electrode in the alkaline media.

(14). *11/221: the “blue shift” seems to be a misalignment of the Raman spectrometer since also the CuO signals are “blue shifted”.*

**Reply:** We thank the reviewer for the comment. Following this comment, we have double-checked the Raman spectrum of electro-oxidized nanoporous Co3Mo/Cu, in which the characteristic Raman bands of Co3O4 at 183, 460, 502 and 658 cm^-1^ remarkably blue shift relative to the ones of pristine Co3O4 (189, 468, 511 and 671 cm^-1^). While the CuO signals in the electro-oxidized Co3Mo/Cu electrode keep the same Raman characteristics as the ones in the electro-oxidized bare Cu. The detailed results are shown in supplementary **Figure 19c**.

**11**

(15). *SI Fig. S2: Add insets from 25-50° 2-Theta to allow for phase assignment and add original references for structural data in caption.*

**Reply:** Following this suggestion, we have added insets from 25 to 50° 2 for phase assignment. At the same time, the original references have been added in the caption. Thanks.

(16). *One major issue in the manuscript is that the electrolyte was not analysed by ICP/OES or better by ICP/MS to determine the concentration of metal ions after the catalysis. This is crucial to know if the materials are stable.*

**Reply:** We thank the reviewer for the suggestion. Following this suggestion, we have analyzed the electrolyte by ICP-OES after the catalysis measurement. There are not any Cu, Co, Mo and Al ions to be detected in the electrolyte after electrocatalysis measurement.

(17). *Another major issue concerns the material´s characterization. It is not clear, where the residual Al is located and what the impact on the catalytic properties are. In addition, there seems to be Mo-rich areas after leaching and some of the experimental data is so light grey in the figures that it is hard to compare to the fits (Fig. 1, Fig. 2, Fig. S7, Fig. S12). Also, many claims by the authors lack experimental proof (e.g. 11/218: Mo-doped Co3O4 – no proof for doping given)*

**Reply:** We appreciate the reviewer for the constructive comment. According to the comment, we have carried out low-energy ion scattering (LEIS) characterization of nanoporous Co3Mo/Cu. As shown in supplementary **Figure 6d**, there is not Al atoms at surface to be detected by LEIS. As shown in high-resolution STEM-EDS mapping (supplementary **Figure 6b,c**), the Al is located at the internal of Cu ligament. This is in agreement with the general expectation in chemical dealloying, in which the less-noble Al on the surface will be selectively etched. In view that catalysis takes place on the ligament surface, internal Al does not essentially affect the catalytic properties of nanoporous Co3Mo/Cu. This is further verified by long-term durability measurement, in which the electrocatalytic activity of nanoporous Co3Mo/Cu does not change remarkably. At the same time, both SEM-EDS mapping (supplementary **Figure 5**) and STEM-EDS mapping (**Figure 1c**) demonstrate that the Co concentration is higher than the Mo.

In addition, we have changed the light grey lines and symbols in **Figure 1**, **Figure 2** and supplementary **Figure 10** and **Figure 16** to more clearly demonstrate the fit result.

12

Regarding the Mo-doped Co3O4, we have double-checked the Raman spectra of EO Co3Mo/Cu and EO Co/Cu electrodes. Distinguished from the CuO signals in the EO Co3Mo/Cu electrode, which keep the same Raman characteristics as the ones in the EO Cu, the characteristic Raman bands at 183, 460, 502 and 658 cm^−1^ remarkably blue shift relative to the ones of pristine Co3O4 (supplementary **Figure 19c**), implying the incorporation of Mo into the Co3O4 nanoflakes. This is further verified by XPS analysis. As shown in supplementary **Figure 19d**, the XPS characteristic peaks of the Co 2p in the EO Co3Mo/Cu electrode shift to lower binding energy compared with the ones in the EO Co/Cu, suggesting the strong electron interactions between Mo and Co3O4.

(18). *Concerning the statement of the authors, that their material is superior to known*

*noble metal-free systems in HER, the following systems outperform theirs:*

*- MoNi/CoMoO3/Nickelfoam (18/10/35 – values as in Tables S1/S2), J. Mater. Chem.*

*A, 2018, 6, 15558.*

*- NiCoMo-Nanowires/Nickelfoam (22/10/34), J. Mater. Chem. A, 2019, 7, 2156.*

**Reply:** We thank the reviewer for the comment. We have listed these values of MoNi/CoMo3/Ni foam and NiCoMo/Ni foam in supplementary **Table 3** to compare with our results. The Tafel slopes (35 and 34 mV dec^−1^) is slightly lower than our value (44 mV dec^−1^). Nevertheless, our nanoporous Co3Mo/Cu electrode exhibits much lower overpotentials (11, 65 and 76 mV) than MoNi/CoMo3/Ni foam (18, 80 and 120 mV) and NiCoMo/Ni foam (22, 120 and 175) to reach the current density of 10, 100 and 200 mA cm^−2^.

(19). *One minor issue: Naming of the materials has to be adopted to the common nomenclature, i.e. Co3Mo/Cu, since the Co3Mo particles are supported on the Cu and not vice versa.*

**Reply:** Following this suggestion, we have re-named the materials according to the common nomenclature, i.e., Co3Mo/Cu, in the whole manuscript.

*Based on the above, I cannot recommend to publish the manuscript.*

**Reply:** Finally, we would like to thank the reviewer again for his/her insightful and constructive comments/suggestions, which substantially promote the scientific significance and quality of this manuscript. Indeed, we feel the paper becomes much more solid and stronger after revision following these comments/suggestions. In view of the novelty, excellent performances and scientific implications of nanostructured intermetallic catalysts, we wish the reviewer could share our confidence and belief

**13**

that the work reported in this paper deserves to be published in the high-impact *Nature Communications*.

**Reviewer comments, second version:**

Reviewers' comments:

Reviewer #1 (Remarks to the Author):

I commend the authors for their considerable efforts to revise the manuscript in line with the reviewer recommendations. The following issues remain to be addressed before I see this work as suitable for publication:

1. The manuscript needs to be edited for grammar and correct terminology. Example: “hydroxygenated” is not valid terminology. I think the authors mean either “oxygenated” or “hydroxylated”

2. Onset potential of 3 mV is not meaningful. Better to say that this catalyst initiates the HER at negligible overpotential, which is characteristic of most metallic HER catalysts.

3. Related to point 2 above, the authors have purged their HER electrolyte with N2, making it impossible to generate a well-defined equilibrium potential for the HER. This distors measurements of absolute overpotential at low current densities, but it does not invalidate the comparison from one electrode to another.

4. Tafel slope measurements in this paper were taken over a narrow range of current densities (less than a factor of 10) at overpotentials less than 100 mV. In this regime, Tafel slope is not a meaningful construct because the Tafel equation assumes the rate of the forward reaction dominates. This assumption is only reasonable at overpotentials above about 100 mV. Unfortunately it is very common in the research literature to report Tafel slopes in this range (even for Pt), but it is incorrect nonetheless. Hence, mechanistic interpretations of the Tafel slope are also not convincing.

5. When reporting overpotentials at 400 mA/cm2 current density, it is important for the authors to note that this is after iR compensation (e.g., by saying “iR-free current density”). This is because at current densities in the hundreds of mA/cm2, iR drop is likely to become the dominant source of overpotential in a practical device.

6. Related to point 5 above, the experimental section needs to report more clearly how iR compensation and RHE calibration were done.

7. The authors’ argument that their catalyst uniquely survive the “violent” conditions of high current density HER is misleading. Numerous other catalysts synthesized by other routes have exhibited comparable geometric activity and considerably greater stability under even harsher conditions. Consider for example the report by Brown and Mahmood over 30 years ago showing nanostructured Ni-Mo alloys doing HER at 500 mA/cm2 and 60 mV overpotential in 30 wt% KOH at 70 deg. C for over 10,000 hours (DOI: 10.1016/0013-4686(84)85008-2). This oversight reflects a consistent pattern of overstatement without adequate discussion of prior work that needs to be addressed.

8. Related to point 7 above, the introduction states that immobilization of catalyst components using insulating binders “...inevitably leads to supplementary and unstable interfaces linked to conductivity and durability issues.” This is also misleading, as PEM electrolyzers with exactly this construction are now commercially available that generate multiple amps/cm2 of current density continuously for thousands of hours. The authors can consult publicly available reports from the US Dept of Energy EERE hydrogen program for some relevant details.

9. The mixture of KOH and NaCl that the authors refer to as “simulated alkaline seawater” is better referred to as “brine electrolyte,” as seawater is a much more complex mixture containing inorganic and organics materials beyond NaCl that may interfere with or foul an electrode. Brine electrolysis is still interesting, as it is used for chlor-alkali production.

10. Supplementary Figure 11 does not provide convincing evidence that the catalyst is significantly reduced under reaction conditions. In fact, the observation of negative current density in the tens of mA/cm2 positive of 0 V vs RHE suggests either some persistent, reversible interfacial redox process or a corrosion reaction occurring alongside the onset of the HER.

11. The comparison of the authors’ Co3Mo/Cu against a Pt control is not convincing since the catalyst loading was almost certainly different in each case, and the Pt/C catalyst film appears not to have been prepared with adequate care to maximize its performance. A cleaner comparison might be to simply use a smooth Pt rotating-disk electrode and then directly compare geometric current densities or TOF values based on ECSA analysis.

12. I don’t agree that the presence of Cu and Al in the electrolyte in Supplementary Table 2 is “ignorable.” This suggests that the skeleton on which the active Co3Mo catalyst is anchored may be dissolving over time, which could ultimately result in catastrophic failure.


Reviewer #3 (Remarks to the Author):

Lang and co-workers report the spontaneously separated intermetallic Co3Mo on the surface of hierarchical nanoporous Cu via a chemical dealloying process. This Co3Mo/Cu has been used as a bifunctional catalyst for high-performance OER/HER and alkaline water electrolysis. Although the results are very interesting in terms of catalytic activity, more clear insights on the surface/bulk structure of the catalysts are needed to understand the reason for the high activity of the catalysts. In addition, several intermetallic structures, including Co3MO have already been published for HER, even with high geometric current densities. Considering the high impact of Nature Communication, I can not recommend this manuscript for acceptance. Some of the reasons for this decision have been given below.

1. Figure 1c shows the inhomogeneous distribution of Co and Mo suggesting the possible formation of elemental Co or Mo particles, in addition to Co3Mo. Did the author carry out oxygen mapping?

2. In the Supplementary Figure S10, the authors have wrongly assigned O2- peak in the O 1s XPS spectra.
3. As the onset overpotential of Co3Mo/Cu was ~3, it would be important to provide the pH of the 1 M KOH solution used for electrocatalysis.

4. It has been shown in the literature that most of the alloy or intermetallic materials undergo a surface transformation in strongly alkaline HER conditions. However, in this report, the authors state that the structure of Co3Mo/Cu stays in the metallic state with hydroxylated surfaces and does not change even after the prolonged HER catalysis (even 1000 h) which they clarify only by providing the ICP and SEM images. This is insufficient. In order to gather insights on the surface structure, the author should conduct HR-TEM, mapping, and XPS.

5. Was there a particular reason to electrooxidize Co3Mo/Cu at 1.57 V? Does it deactivate under direct LSV condition?

6. The Mo-doping into the Co3O4 was only confirmed by the blue-shift in Raman spectra. Why haven’t the Mo 3d and O 1s XPS spectra (or EDX mapping) been shown to confirm the surface molybdenum (oxide) sites?

7. The characterization of EO Co3Mo/Cu after OER catalysis is completely missing in the manuscript.

8. Besides, a short discussion on the surface structure and nature of the active sites in OER/HER would be very helpful to illustrate how one could design new strategies to achieve bifunctional electrocatalysts starting from intermetallic compounds.

9. The authors state that the Pt/C-Ir/C catalysts are poor for alkaline electrolysis; however, their mass loading is very low compared to Co3Mo/Cu or EO Co3Mo/Cu.

**Author rebuttal, second version:**

***Reviewer #1 (Remarks to the Author):***

*I commend the authors for their considerable efforts to revise the manuscript in line with the reviewer recommendations. The following issues remain to be addressed before I see this work as suitable for publication:*

**Reply:** We thank the reviewer for his/her positive comments. We also appreciate the reviewer for his/her further comments and constructive suggestions. Following these valuable and insightful comments/suggestions, we have completely revised the manuscript. The detailed corrections are listed below.

(1) *The manuscript needs to be edited for grammar and correct terminology. Example: “hydroxygenated” is not valid terminology. I think the authors mean either “oxygenated” or “hydroxylated”.*

**Reply:** We appreciate the reviewer for the comment, following which we have corrected these invalid terminologies and grammar errors in the manuscript.

(2) *Onset potential of 3 mV is not meaningful. Better to say that this catalyst initiates the HER at negligible overpotential, which is characteristic of most metallic HER catalysts.*

**Reply:** Following this constructive suggestion, we have corrected the presentation on the onset overpotential.

(3) *Related to point 2 above, the authors have purged their HER electrolyte with N2, making it impossible to generate a well-defined equilibrium potential for the HER. This distors measurements of absolute overpotential at low current densities, but it does not invalidate the comparison from one electrode to another.*

**Reply:** We appreciate the reviewer for his/her valuable comment, according to which we have made correction on the onset overpotential.

(4) *Tafel slope measurements in this paper were taken over a narrow range of current densities (less than a factor of 10) at overpotentials less than 100 mV. In this regime, Tafel slope is not a meaningful construct because the Tafel equation assumes the rate of the forward reaction dominates. This assumption is only reasonable at overpotentials above about 100 mV. Unfortunately it is very common in the research literature to report Tafel slopes in this range (even for Pt), but it is incorrect*

1

*nonetheless. Hence, mechanistic interpretations of the Tafel slope are also not convincing.*

**Reply:** We appreciate the reviewer for his/her valuable comments and constructive suggestion. Following the insightful comment, we have extended the range of current densities in the Tafel plots and re-valuated the Tafel slopes (**Figure 3b**).

(5) *When reporting overpotentials at 400 mA/cm^2^ current density, it is important for the authors to note that this is after iR compensation (e.g., by saying “iR-free current density”). This is because at current densities in the hundreds of mA/cm^2^, iR drop is likely to become the dominant source of overpotential in a practical device.*

**Reply:** We thank the reviewer for his/her constructive suggestion, following which we have noted the iR correction when reporting overpotentials at current density of 400 mA cm^−2^.

(6) *Related to point 5 above, the experimental section needs to report more clearly how iR compensation and RHE calibration were done.*

**Reply:** We appreciate the reviewer for the constructive suggestion. Following the suggestion, we have presented the detailed RHE calibration and iR compensation in Method section. The detailed result has been shown in supplementary **Figure 13**, wherein the potential (1.016 V) at which the current crossed zero was taken to be the thermodynamic potential for the hydrogen electrode reaction. Therefore, the potentials were calibrated to RHE according to *E*RHE = *E*Ag/AgCl + 1.016 V. The iR compensation is carried out in terms of *E*RHE = *E*Ag/AgCl + 1.016 V – iR.

(7) *The authors’ argument that their catalyst uniquely survive the “violent” conditions of high current density HER is misleading. Numerous other catalysts synthesized by other routes have exhibited comparable geometric activity and considerably greater stability under even harsher conditions. Consider for example the report by Brown and Mahmood over 30 years ago showing nanostructured Ni-Mo alloys doing HER at 500 mA/cm^2^ and 60 mV overpotential in 30 wt% KOH at 70 deg. C for over 10,000 hours (DOI: 10.1016/0013-4686(84)85008-2). This oversight reflects a consistent pattern of overstatement without adequate discussion of prior work that needs to be addressed.*

**Reply:** We appreciate the reviewer for the valuable comment. According to this comment, we have corrected the description on the nanoporous Co3Mo/Cu electrode, which is performed at mild conditions, i.e., in 1 M KOH electrolyte at room temperature. As demonstrated by systemic characterizations, our nanoporous

2

Co3Mo/Cu electrode can exhibit impressive electrocatalytic performance comparable to Ni-Mo alloys under harsher conditions, i.e., in KOH electrolyte with higher concentrations or/and at higher temperatures, which are neither environmentally friendly nor cost-effective. This implies the potential application of nanoporous Co3Mo/Cu electrode in mild conditions.

(8) *Related to point 7 above, the introduction states that immobilization of catalyst components using insulating binders “...inevitably leads to supplementary and unstable interfaces linked to conductivity and durability issues.” This is also misleading, as PEM electrolyzers with exactly this construction are now commercially available that generate multiple amps/cm^2^ of current density continuously for thousands of hours. The authors can consult publicly available reports from the US Dept of Energy EERE hydrogen program for some relevant details.*

**Reply:** We thank the reviewer for his/her comments. After consulting the reports from the US DOE, we have made correction to avoid the misleading presentation on the stability issue of immobilized nanocatalysts.

(9) *The mixture of KOH and NaCl that the authors refer to as “simulated alkaline seawater” is better referred to as “brine electrolyte,” as seawater is a much more complex mixture containing inorganic and organics materials beyond NaCl that may interfere with or foul an electrode. Brine electrolysis is still interesting, as it is used for chlor-alkali production.*

**Reply:** We appreciate the reviewer for his/her valuable suggestion. According to this suggestion, we have corrected the presentation on the mixed electrolyte of KOH and NaCl in the manuscript.

(0) *Supplementary Figure 11 does not provide convincing evidence that the catalyst is significantly reduced under reaction conditions. In fact, the observation of negative current density in the tens of mA/cm^2^ positive of 0 V vs RHE suggests either some persistent, reversible interfacial redox process or a corrosion reaction occurring alongside the onset of the HER.*

**Reply:** We thank the reviewer for the comment. In the present work, our nanoporous Co3Mo/Cu electrode is fabricated by a facile and scalable dealloying technology associated with a spontaneous phase separation. In the 6 M KOH aqueous solution, it is reasonable that the as-prepared nanoporous Co3Mo/Cu electrode has surface Cu, Co and Mo atoms to be in the oxidized states. For example, as shown in **Figure 2a**, the surface Co atoms exhibit Co^2+^ state while the surface Mo atoms are in Mo^4+^ and Mo^6+^ states due to the adsorption of hydroxyl groups (supplementary **Figure 10**). These

3

oxidized Co and Mo surface atoms are electro-reduced to form partially hydroxylated Co3Mo surface even at the positive potential vs RHE, during which there reasonably observes the negative current density (supplementary **Figure 11**). This is further verified by DFT calculations based on meta-generalized gradient approximation with strongly constrained appropriately normed functional. As shown in supplementary **Figure 12**, the coverage of hydroxyl groups on the Co3Mo(002) surface decreases with the potential decreasing from 0.6 to 0 V vs RHE. As a consequence, the negative current density in the tens of mA cm^−2^ positive of 0 V vs RHE should be attributed to the partial electro-reduction of surface Cu, Co and Mo atoms.

*(11) The comparison of the authors’ Co3Mo/Cu against a Pt control is not convincing since the catalyst loading was almost certainly different in each case, and the Pt/C catalyst film appears not to have been prepared with adequate care to maximize its performance. A cleaner comparison might be to simply use a smooth Pt rotating-disk electrode and then directly compare geometric current densities or TOF values based on ECSA analysis.*

**Reply:** We thank the reviewer for the valuable comment and the constructive suggestion. Nevertheless, we cannot perform supplementary experimental measurements on a smooth Pt rotating-disk electrode because of the influence of COVID-19. Now that all laboratories in our university are closed and all students stay home, we appreciate the reviewer for his/her understanding of this situation. To demonstrate the reliability of our electrochemical measurements on Pt/C catalyst, we compare the overpotentials at various current densities for the Pt/C catalysts casted on glassy carbon electrode (Pt/C/GCE) reported in this work and in previous literature, respectively. At the same time, the related values on Pt foil or Pt metal are also included for comparison. As shown in below **Table I**, the overpotentials at the current densities of 10, 50, 100 and 200 for the Pt/C/GCE in our previous measurement is comparable to the corresponding values that are previously reported in literature. This implies the reliability of our electrochemical measurements. When Pt/C catalyst is casted on nanoporous Cu skeleton (Pt/C/Cu), the overpotentials at various current densities are slightly lower than the values of Pt/C/GCE and Pt foil and Pt metal electrodes. Furthermore, in view of the nanoporous Pt/C/Cu electrode having an electrode architecture similar to the nanoporous Co3Mo/Cu, it should be reasonable to compare their electrochemical performance to demonstrate the superior properties of the latter.

**Table I.** Comparisons of the HER catalytic activity of nanoporous Pt/C/Cu, Pt/C/GCE and Pt foil/metal electrodes in 1 M KOH electrolyte.

4

***ηϳ***

***ϳ***

**Electrocatalysts	Refs.**

**(mV) (mA cm−^2^)**

|  | 11 | 10 |  |
| --- | --- | --- | --- |
|  | 54 | 50 |  |
|  | 65 | 100 |  |
| Co3Mo/Cu |  |  | This work |
|  | 76 | 200 |  |
|  | 86 | 300 |  |
|  | 96 | 400 |  |

| 50 10  20 wt% Pt/C 147 50  This work  on nanoporous Cu 195 100  240 200 | | | |
| --- | --- | --- | --- |
| 20 wt% Pt/C  on glassy carbon electrode  (0.2 mg cm^-2^) | 61  145  208  319 | 10  50  100  200 | This work |
| 20 wt% Pt/C 45 10  *Nat. Commun.* **10**, 269  on glassy carbon electrode - 50  (0.2 mg cm^-2^) (2019)  250 100 | | | |
| Pt foil | 60  240  300 | 10  50  100 | *Nat. Commun.* **10**, 269  (2019) |
| 20 wt% Pt/C 40 10  *J. Am. Chem. Soc.* **140**,  on glassy carbon electrode 170 50  (0.12 mg cm^-2^) 5118-5126 (2018)  250 100 | | | |
| Pt metal | 100  180  250 | 10  50  100 | *J. Am. Chem. Soc.* **140**,  5118-5126 (2018) |
| 10 wt% Pt/C 50 10  *Nat. Commun.* **10**, 1743  on glassy carbon electrode 130 50  (2.04 mg cm^-2^) (2019)  200 100 | | | |
| 20 wt% Pt/C  on glassy carbon electrode  (1.02 mg cm^-2^) | 80  150  200  300 | 10  50  100  200 | *Nat. Commun.* **9**, 2533  (2018) |
| 20 wt% Pt/C 60 10 *Adv. Mater.* **32**, 1906972  on glassy carbon electrode 150 50 (2020) | | | |
| Pt plate | 50  100  140 | 10  50  100 | *Adv. Mater.* **31**, 1806769  (2019) |
| Pt metal 110 10 *Nat. Commun.* **11**, 1116  150 50 (2020) | | | |
| 20 wt% Pt/C  on glassy carbon electrode | 70  100 | 10  50 | *Adv. Mater.* **32**, 1905679  (2020) |

5

*(12) I don’t agree that the presence of Cu and Al in the electrolyte in Supplementary Table 2 is “ignorable.” This suggests that the skeleton on which the active Co3Mo catalyst is anchored may be dissolving over time, which could ultimately result in catastrophic failure.*

**Reply:** We thank the reviewer for the suggestion, following which we have corrected the presentation. Despite there is detectable Cu or/and Al in the tested electrolyte, the nanoporous Co3Mo/Cu electrode after the 1000-h stability measurements still keeps the original structure and exhibits almost the same LSV curve as the initial one (insets of **Figure 3g**). These results demonstrate the stability of nanoporous Co3Mo/Cu electrode for the HER.

6

***Reviewer #3 (Remarks to the Author):***

*Lang and co-workers report the spontaneously separated intermetallic Co3Mo on the surface of hierarchical nanoporous Cu via a chemical dealloying process. This Co3Mo/Cu has been used as a bifunctional catalyst for high-performance OER/HER and alkaline water electrolysis. Although the results are very interesting in terms of catalytic activity, more clear insights on the surface/bulk structure of the catalysts are needed to understand the reason for the high activity of the catalysts. In addition, several intermetallic structures, including Co3Mo have already been published for HER, even with high geometric current densities. Considering the high impact of Nature Communication, I cannot recommend this manuscript for acceptance. Some of the reasons for this decision have been given below.*

**Reply:** We thank the reviewer for finding interest of our work. We also appreciate the reviewer for the valuable comments and constructive suggestions. Following these comments/suggestions, we have presented additional results, including STEM-EDS mapping of oxygen on nanoporous Co3Mo/Cu electrode, Co 2p and Mo 3d XPS spectra of nanoporous Co3Mo/Cu electrode after durability measurement, SEM-EDS mapping of Cu, Co, Mo and O elements and their XPS spectra and Raman spectrum of EO Co3Mo/Cu electrode before and after durability measurement. Based on these results, we have completely revised the manuscript. The detailed corrections are listed below.

We agree with the reviewer that several nonprecious intermetallic compounds have been investigated for the electrocatalytic HER in alkaline environments because of their low cost, high electrocatalytic activities and good electrochemical durability. By virtue of intermetallic compounds being composed of dissimilar metals with ordered crystal structures, they are ideal platform materials to optimize catalytic activity by adjusting adsorption energy of reactant intermediates via ligand effect and strain effect. However, despite the initial strides have been made, the superior electrocatalytic performance of intermetallic compounds, particularly Co3Mo, have not been fully exploited for practical applications in large-scale production of hydrogen via electrochemical water splitting. It is well-known that in the water electrolysis, the HER/OER taking place at the cathode/anode are associated with mass transports of ions/molecules toward/from the electrochemical surface in the electrolyte, electron transfer from current collector to active sites in the solid electrode, and redox reactions at the active site/electrolyte interface. These indispensable processes require versatile electrode materials to comprise sufficient accessible surface with high intrinsic activity and to have a rational architecture with capability of electron transfer and mass transport for highly efficient HER/OER electrocatalysis.

7

In comparison with state-of-the-art electrode materials of intermetallic compounds, we would like to address that our monolithic nanoporous Co3Mo/Cu electrode are intrinsically different from all of them in both electrode architecture and physical/chemical properties, which result in the outstanding electrocatalytic performance. In the previous reports on intermetallic compound-based electrode materials, they are usually prepared by thermal reduction of nanostructured multi-metal oxides at high temperature. Therein, the electroactive intermetallic nanoparticles are supported by or embedded in metal oxide matrix/substrate. Such electrode architecture not only suffers from a poor electron transfer from conductive substrate to electroactive sites but limits the accessibility of electroactive sites. In contrast, in this work we propose a facile and scalable dealloying technology associated with a spontaneous phase separation to develop electrode materials for satisfying the versatile requirements in both electrode structure and active site levels. Based on designing Cu12*-x-y*Co*x*Mo*y*Al88 precursor alloys with a quasi-eutectic multiphase structure, monolithic nanoporous Co3Mo/Cu electrodes that are composed of intermetallic Co3Mo nanoparticles seamlessly integrated on a three-dimensional bicontinuous and bimodal nanoporous Cu skeleton are fabricated by making use of the spontaneous phase separation due to the poor miscibility of intermetallic Co3Mo with Cu matrix. Therein, the interconnective Cu ligaments and the interpenetrating large channels simultaneously facilitates the electron transfer and the mass transports of HO^-^/H2O, respectively, while the small nanopores raise the accessibility of intermetallic Co3Mo nanoparticles seamlessly integrated on Cu ligaments. As a result of hydroxylation of the intermetallic Co3Mo surface, which benefits for the subsequent adsorption/desorption of H* intermediates as a result of their optimum HBE and thermoneutral ΔGH*, the constituent Co3Mo nanoparticles are further demonstrated to serve as the electroactive sites. When triggered by electro-oxidation, the hydroxylated Co3Mo evolves into the Co3O4 nanoflakes with the in-situ incorporation of Mo, which improves the electronic conductivity of Co3O4 and adjusts the adsorption energy of HO*, remarkably boosting the OER electrocatalysis. These versatile properties of nanoporous Co3Mo/Cu electrode in turn demonstrate the feasibility of dealloying quasi-eutectic multiphase alloys as a facile strategy to develop high-performance intermetallic compound-based electrocatalysts for the HER/OER.

Considering the advantages of nanoporous Co3Mo/Cu electrode in both architecture and active site levels, we have presented a systematic discussion with more clear insights on the surface/bulk structure of the catalysts. After revision with these constructive comments and suggestions, we feel the paper becomes much more solid and stronger, as well as easier for readers to understand the novelty and significance

8

of this work. In view of the novelty, excellent performances and reproducibility and scientific implications of the nanoporous intermetallic compound/metal electrodes for the HER and their electro-oxidized derivatives for the OER, we wish the reviewer could share our confidence and belief that the work reported in this paper deserves to be published in a high-impact journal, like *Nature Communications*.

(1) *Figure 1c shows the inhomogeneous distribution of Co and Mo suggesting the possible formation of elemental Co or Mo particles, in addition to Co3Mo. Did the author carry out oxygen mapping?*

**Reply:** We thank the reviewer for the suggestion, according to which we have presented the oxygen mapping image in supplementary **Figure 6**.

(2) *In the Supplementary Figure S10, the authors have wrongly assigned O2- peak in the O 1s XPS spectra.*

**Reply:** According to the comment, we have re-performed analysis on the O 1s XPS spectrum in supplementary **Figure 10**. The O 1s spectrum can be deconvolated into three characteristic peaks of oxygen atoms in HO bond (531.2 eV, O1), surface-adsorbed oxygen (532.6 eV, O2) and adsorbed molecular water (533.4 eV, O3).

(3) *As the onset overpotential of Co3Mo/Cu was ~3, it would be important to provide the pH of the 1 M KOH solution used for electrocatalysis.*

**Reply:** We thank the reviewer for the suggestion. Following this suggestion, we have provided the pH value of 1 M KOH solution used for electrocatalysis in Method.

(4) *It has been shown in the literature that most of the alloy or intermetallic materials undergo a surface transformation in strongly alkaline HER conditions. However, in this report, the authors state that the structure of Co3Mo/Cu stays in the metallic state with hydroxylated surfaces and does not change even after the prolonged HER catalysis (even 1000 h) which they clarify only by providing the ICP and SEM images. This is insufficient. In order to gather insights on the surface structure, the author should conduct HR-TEM, mapping, and XPS.*

**Reply:** We appreciate the reviewer for the constructive suggestion. Unfortunately, we cannot perform supplementary characterizations on the nanoporous Co3Mo/Cu electrode after long-term durability measurement due to the influence of COVID-19. Now that all laboratories in our university are closed and all students stay home, we thank the reviewer for his/her understanding of this situation. According to the previous electrochemical measurements and structure characterization, our

*9*

nanoporous Co3Mo/Cu electrode indeed exhibits exceptional stability. As shown in **Figure 3g**, there is not evident fluctuation in the current densities of ~60 and ~210 mA cm−^2^ at the overpotentials of 60 and 80 mV. Even for more than 1000 h, the nanoporous Co3Mo/Cu electrode still keeps the original structure and exhibits almost the same HER polarization curve as the initial one (insets of **Figure 3g**). Furthermore, there are no Co and Mo to be detected by ICP-OES in the tested electrolyte. In view of the recent situation, here we present the Mo 3d and Co 2p XPS spectra of nanoporous Co3Mo/Cu electrode after stability measurement at the overpotentials of 60, 80 and 100 mV for each 10 h (**Figure Ia**), which were characterized at the end of last year. As shown in below **Figure Ib,c**, the Co and Mo atoms in the nanoporous Co3Mo/Cu electrode maintain almost the same chemical states as that before durability measurement (**Figure 2a**). This observation implies the stability of chemical states of Co and Mo in the nanoporous Co3Mo/Cu electrode during the alkaline HER electrocatalysis.

**Figure I**. **a**, Polarization curves before and after stability measurement of nanoporous Co3Mo/Cu. Inset: Stability measurement of nanoporous Co3Mo/Cu electrode at the overpotentials of 60, 80 and 100 mV for each 10 h, respectively. **b**,**c**, High-resolution XPS spectra of Mo 3d (b) and Co 2p (c) of nanoporous Co3Mo/Cu electrode after the stability measurement for 30 h.

10

(5) *Was there a particular reason to electrooxidize Co3Mo/Cu at 1.57 V? Does it deactivate under direct LSV condition?*

**Reply:** We thank the reviewer for the comment. Generally, the preparation of EO Cu3Mo/Cu electrode can be performed at voltages that can trigger the electro-oxidization of nanoporous Co3Mo/Cu. To produce effectively EO Cu3Mo/Cu electrode and judge synchronously its electrocatalytic activity towards the OER at overpotential of ~0.34 V, we perform the electro-oxidation of nanoporous Co3Mo/Cu at 1.57 V. Furthermore, it does not deactivate under direct LSV condition.

(6) *The Mo-doping into the Co3O4 was only confirmed by the blue-shift in Raman spectra. Why haven’t the Mo 3d and O 1s XPS spectra (or EDX mapping) been shown to confirm the surface molybdenum (oxide) sites?*

**Reply:** We thank the reviewer for his/her constructive suggestion. To confirm the surface Mo sites, we have performed EDS mapping characterization. The detailed elemental distribution of Co, Mo and O are shown in supplementary **Figure 21**. The presence of surface Mo atoms is also verified by Mo 3d XPS spectrum in spite of the weak characteristic peak (supplementary **Figure 20**). In addition, O 1s XPS spectrum is also shown in supplementary **Figure 20**.

(7) *The characterization of EO Co3Mo/Cu after OER catalysis is completely missing in the manuscript.*

**Reply:** We appreciate the reviewer for the comments, following which we have carried out supplementary EDS mapping characterization. As shown in supplementary **Figure 22**, the EO Co3Mo/Cu electrode after the 30 h OER stability test still keeps almost the same morphology and elemental distributions of Cu, Co, Mo and O as the initial one, implying the good stability during the OER. Furthermore, there are not evident changes in Raman spectra and Co 2p, Mo 3d and O 1s XPS spectra (supplementary **Fig. 23** and **24**), demonstrating the stability of surface oxides.

(8) *Besides, a short discussion on the surface structure and nature of the active sites in OER/HER would be very helpful to illustrate how one could design new strategies to achieve bifunctional electrocatalysts starting from intermetallic compounds.*

**Reply:** We appreciate the reviewer for the constructive suggestion. According to this suggestion, we have presented discussion on intermetallic compounds as promising bifunctional electrocatalysts towards OER/HER in Discussion section.

(9) *The authors state that the Pt/C-Ir/C catalysts are poor for alkaline electrolysis;*

**11**

*however, their mass loading is very low compared to Co3Mo/Cu or EO Co3Mo/Cu.*

**Reply:** We appreciate the reviewer for the comment. In our experiment, the Pt/C and Ir/C catalysts are loaded on nanoporous Cu skeleton (Pt/C/Cu and Ir/C/Cu) for all electrochemical measurements, including the overall water splitting in brine electrolyte. Considering the surface effect of catalysis, it is reasonable to compare the performance of Co3Mo/Cu-based electrolyzer with the one constructed with Pt/C/Cu and Ir/C/Cu. The poor performance of electrolyzer based on Pt/C/Cu and Ir/C/Cu is probably due to the polymer binder that covers some electroactive sites and blocks electron transfer from electroactive sites to current collector

**Reviewer comments, third version:**

REVIEWERS' COMMENTS:

Reviewer #3 (Remarks to the Author):

In this revised version, the authors have significantly enhanced the quality of their manuscript. Although the authors could not conduct some of the experiments quite understandably due to the ongoing COVID-19 crisis, they have satisfactorily addressed most of my previous comments as well as those of Reviewer 1 very well. Based on this, I feel that the present version of the manuscript has a good impact and is of interest to the broad readership of Nature Communications

**Author rebuttal, third version:**

*Reviewer #3 (Remarks to the Author): In this revised version, the authors have significantly enhanced the quality of their manuscript. Although the authors could not conduct some of the experiments quite understandably due to the ongoing COVID-19 crisis, they have satisfactorily addressed most of my previous comments as well as those of Reviewer 1 very well. Based on this, I feel that the present version of the manuscript has a good impact and is of interest to the broad readership of Nature Communications.*

*Reply: We appreciate the reviewer for his/her positive comments and recommendation for publication in Nature Communications*
